# Excited State Dynamics Govern Emission Properties of Unique Silsesquioxane-Salphen-Based Zinc Compounds

**DOI:** 10.1021/acs.jpclett.4c03406

**Published:** 2025-03-03

**Authors:** Joanna Szymkowiak, Tomasz Pędziński, Beata Dudziec

**Affiliations:** † Center for Advanced Technologies, Adam Mickiewicz University in Poznan, Uniwersytetu Poznanskiego 10, 61-614 Poznan, Poland; ‡ Faculty of Chemistry, Adam Mickiewicz University in Poznan, Uniwersytetu Poznanskiego 8, 61-614 Poznan, Poland

## Abstract

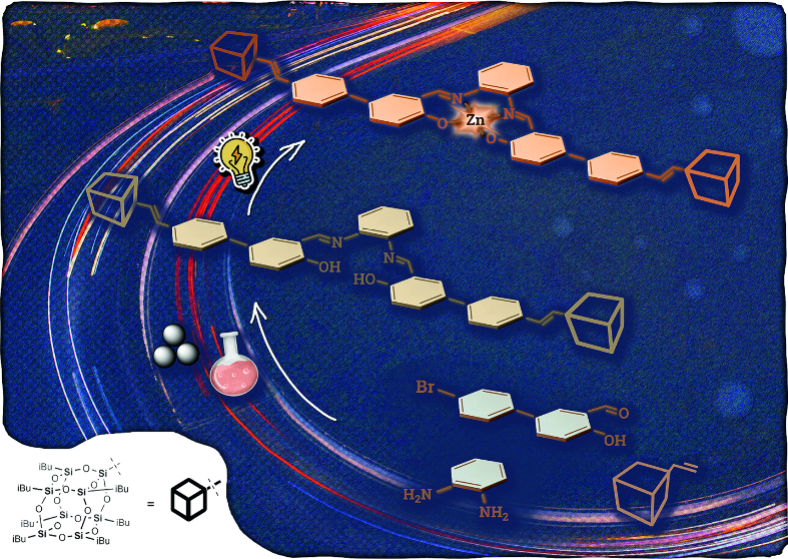

This study aims to develop a synthetic protocol for preparing salphen-based hybrid compounds with silsesquioxane T_8_ cages anchored at the molecule’s periphery. Three types of coordination compounds featuring κ^4^*-N*_*2*_*O*_*2*_-donating atoms were obtained via a sequence of reactions. These compounds differ in the arene linker between the salphen and silsesquioxane fragments. An individual synthetic pathway was developed for the preparation of aldehydes, followed by a tailored strategy for the synthesis of the final complexes employing both solution-based and mechanochemical methods in the solid state. The latter represents a novel technique in silsesquioxane chemistry. The newly designed ligands were used for the coordination of Zn^2+^ ions to evaluate their ligation properties and to determine the photophysical properties of the resulting complexes in comparison to their bare ligand molecules. Using absorption and emission spectroscopy, combined with advanced time-resolved spectroscopic methods, we demonstrated that the photochemical efficiency of these compounds is influenced by their tendency to aggregate in solution, which positively affects their photophysical properties and enhances their potential for photodynamic therapy (PDT). Additionally, we explored the ability of these complexes to generate singlet oxygen (^1^O_2_) depending on the architecture of the designed ligands. The results indicate that the excited state dynamics plays a crucial role in determining the emission properties of the studied compounds, which may have significant implications for their applications in medicine and materials science.

The design and synthesis of specialized materials are among the most important challenges in contemporary chemistry and materials engineering.^1^ There is a demand for materials that combine special functions i.e. supramolecular recognition or ion detection with enhanced thermal and mechanical properties. The first part of this combination can be attributed to an organic fragment with embedded functional groups, while the second part may be represented by a specific class of organosilicon compounds, i.e. silsesquioxanes (POSS,^2^ SQs). These systems are a class of well-defined hybrid organosilicons with inorganic Si–O–Si cores and organic coronae, constituting hybrid molecules of the general formula (RSiO_3/2_)_*n*_ where *n* is even up to 18 but never less than 6, and characterized by nanoscale size and diverse architectures, i.e. random, ladder, cage, and partially caged.^3,4^ Within this group, the cube-like T_8_ derivatives are most widely applied even though double-decker silsesquioxanes (DDSQ) have received significant attention.^5,6^ For their exclusive physicochemical properties silsesquioxane may be found applied in many branches of chemistry, in materials (as building blocks or polymer modifiers)^7−10^ optoelectronics (i.e., sensors, detectors and OLED devices),^11−16^ catalysis,^17−20^ or in medicine, e.g. in drug delivery systems or antibiotics removal, part of artificial tissues or dental appliance.^21−25^ In regards to conventional luminescent materials a photophysical phenomenon called aggregation-caused quenching (ACQ) may be noted, which is harmful for their possible use in optoelectronics.^26^ The use of silsesquioxanes impacts the pendant nature of the chromophores in these materials’ architectures and can result in enhanced emission. Additionally, the inorganic core of SQs is also reported in the electron transfer between the conjugated arenes that affects their fluorescence properties.^27,28^ Owing to the attractive characteristics of silsesquioxanes, combining the properties of a silica-based inorganic core with easily tunable organic groups attached to it, some attempts have been made to employ them as specialized ligands in transition metal (TM) coordination compounds.

These studies demonstrate the potential to anchor ligands with well-defined donor properties, particularly polydentate (chelating) ones, enabling the fine-tuning of metal coordination environments. Various ligand types, including diketones,^29^ carboxylic acids,^30^ 8-hydroxy-quionoline,^31,32^*N*,*O*-Schiff imines,^12,20^ and terpyridine derivatives,^33−35^ have been successfully integrated into POSS-based architectures. The silsesquioxane core itself offers diverse functionalization possibilities, ranging from mono- and difunctionalized to octafunctionalized systems, which significantly impact the structural organization and reactivity of the resulting complexes. The range of metals incorporated into these frameworks remains primarily limited to transition metals such as Ru, Fe, Cu, and Pt, alongside selected lanthanides such as Eu and Tb, which impart unique optical and electronic characteristics to the resulting materials. Depending on the coordination nature, these hybrid systems can adopt different structural motifs including discrete 0D molecules, macromolecular 1D coordination polymers, or more complex 3D coordination networks. Such architectures have demonstrated significant promise in applications such as luminescent materials, molecular electronics,^33,36−39^ and catalysis,^40,41^ with particular interest in their use for photo- and electroactive device fabrication. Studies have shown that POSS-based luminescent materials can exhibit tunable photophysical properties due to controlled metal–ligand interactions and supramolecular assembly behavior. Despite these advances, a notable gap exists regarding their potential application as photosensitizers for singlet oxygen generation, which is crucial for photodynamic therapy (PDT). Although related systems have been extensively investigated for their emission properties and catalytic activity, no reports have specifically addressed their role in PDT-active materials. Given the intrinsic photophysical characteristics of certain metal complexes and the stability of POSS-based architectures, further exploration of their potential as efficient singlet oxygen generators could open new pathways in biomedical applications.

Since Schiff’s achievements in the field of organic bases were published, imines have become one of the most popular supramolecular synthons in materials chemistry.^42,43^ Interestingly, the name of this tetradentate ligand is affected by the type of construction of the diamine and salicylaldehyde units. Among these ligands, salenes/salphenes are probably the most popular. There are reports of their efficient use as catalysts via examples of Mn^III^-salenes developed by Jacobsen (1991) and Katsuki (1994)^44,45^ for the enantioselective epoxidation of olefins and other catalysts based on transition metals, e.g. Fe^II^, Co^II^, Ni^II^, Zn^II^, Cu^II^ (used in Henry reaction, sulfoxidation, cycloadditions, etc.) that convey a whole group of catalysts described as M(salen)-based materials.^42,46^ The presence of *N*_2_*O*_2_-donor atoms in the molecular skeleton of Lewis bases, forming a unique salen pocket, influences not only their self-associative properties but also their ligating abilities and biomedical potential.^42,47^

Interestingly, these systems exhibit attractive photophysical and redox properties affecting the direction of their use.^48−50^ Embedding imine groups on the SQs core may result in novel coordination materials that can be used as host–guest systems, e.g. in catalysis.^20^ However, few reports combine salen moieties with silsesquioxanes, and a significant gap exists not only in the synthetic protocol leading to their formation but also in their properties, especially of interest for their potential in use.

This work aims to design salphen-based hybrid compounds with anchored silsesquioxane T_8_ cages at the periphery, varying in the construction of the linker between the salphen core (Figure 1) and their photophysical properties. A synthetic protocol to gain the target compounds was designed, and three types of coordination compounds with κ^4^*-N*_*2*_*O*_*2*_-donating atoms were obtained in a sequence of reactions. They differ in the aromatic linker between the salphen and silsesquioxane fragments. Obtained compounds were used for coordination of Zn^2+^ ions to verify their ligation properties and determine the photophysical properties of the resulting complexes in comparison to the bare ligands. Using absorption and emission spectroscopy, combined with advanced time-resolved spectroscopic methods, we demonstrate that the photochemical effectiveness of these compounds is influenced by their tendency to aggregate in solution, which positively impacts their photophysical properties and enhances their potential, e.g. in photodynamic therapy (PDT). Additionally, we explored the ability of these complexes to generate singlet oxygen (^1^O_2_), depending on the architecture of the designed ligands.

**Figure 1:**
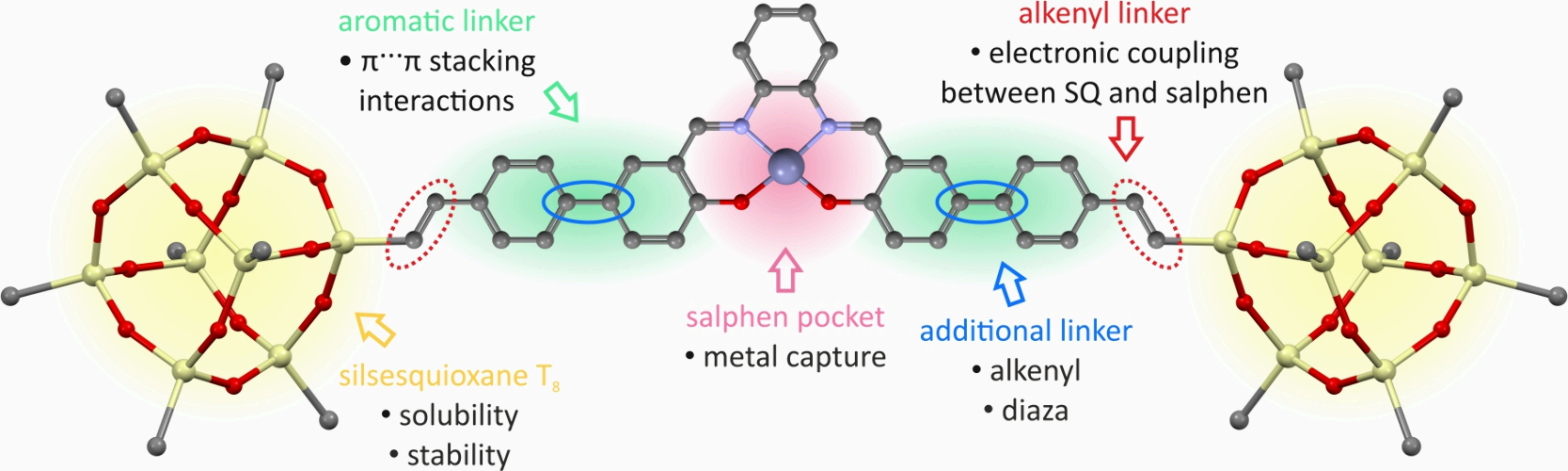
Illustration of the design concept for a hybrid coordination system architecture.

*Synthesis of silsesquioxane-salphen-based zinc compounds*. The silsesquioxane-salphen-based zinc complex architectures consist of two imine units attached to the phenyl ring in the *ortho*- position as well as two hydroxy-phenyl fragments. These derive from the nucleophilic addition of 1,2-phenylenediamine and 2-hydroxybenzaldehyde, named after Schiff.^51^ In our case, we modified the structure of a 2-hydroxybenzaldehyde derivative to contain silsesquioxane T_8_ fragments (**SQ-1**, **SQ-2**, **SQ-3**). The protocol leading to its formation is based on a sequence of reactions based on Pd-mediated coupling processes. A Suzuki-Miyaura coupling was performed between 5-iodosalicylaldehyde and 4-bromophenylboronic acid in the presence of [Pd(PPh_3_)_4_] to efficiently obtain 4′-bromo-4-hydroxy-[1,1′-biphenyl]-3-carbaldehyde (**ald-1**).^52^ The (*E*)-5-(4-bromostyryl)-2-hydroxy-benzaldehyde, with 1,2-ethene-bridge (**ald-2**), was obtained in a Heck coupling using 5-iodosalicylaldehyde and 4-bromostyrene with Pd[P(*t*-Bu)_3_]_2_ as catalyst.^53^ The compound containing the 1,2-diaza- (**ald-3**) linker was obtained via diazotisation reaction followed by oxidation using a modified synthetic path described by Pawar et al., based on nitrosation of 4-bromophenylamine, to form the diazonium salt that acts as electrophile in coupling 2-hydroxybenzaldehyde.^54^

The obtained aldehydes (**ald-1**, **ald-2**, **ald-3**) possess a bromo-substituent at the 4′- position of the phenyl ring that was susceptible to Heck coupling, i.e. the use of a vinyl(heptaisobutyl)octasilsesquioxane (**SQ-Vi**). The coupling reaction was carried out using Pd[P(*t*-Bu)_3_]_2_ and *N*,*N*-dicyclohexylmethylamine,^28^ giving the respective aldehydes with T_8_ cages attached via ethenyl moieties (**SQ-1**), resulting in direct phenyl–phenyl rings connection (**SQ-1**) and those with 1,2-ethene- (**SQ-2**) and 1,2-diaza- (**SQ-3**) linkages that structures exhibited highly conjugated π-system (Scheme 1).

**Scheme 1:**
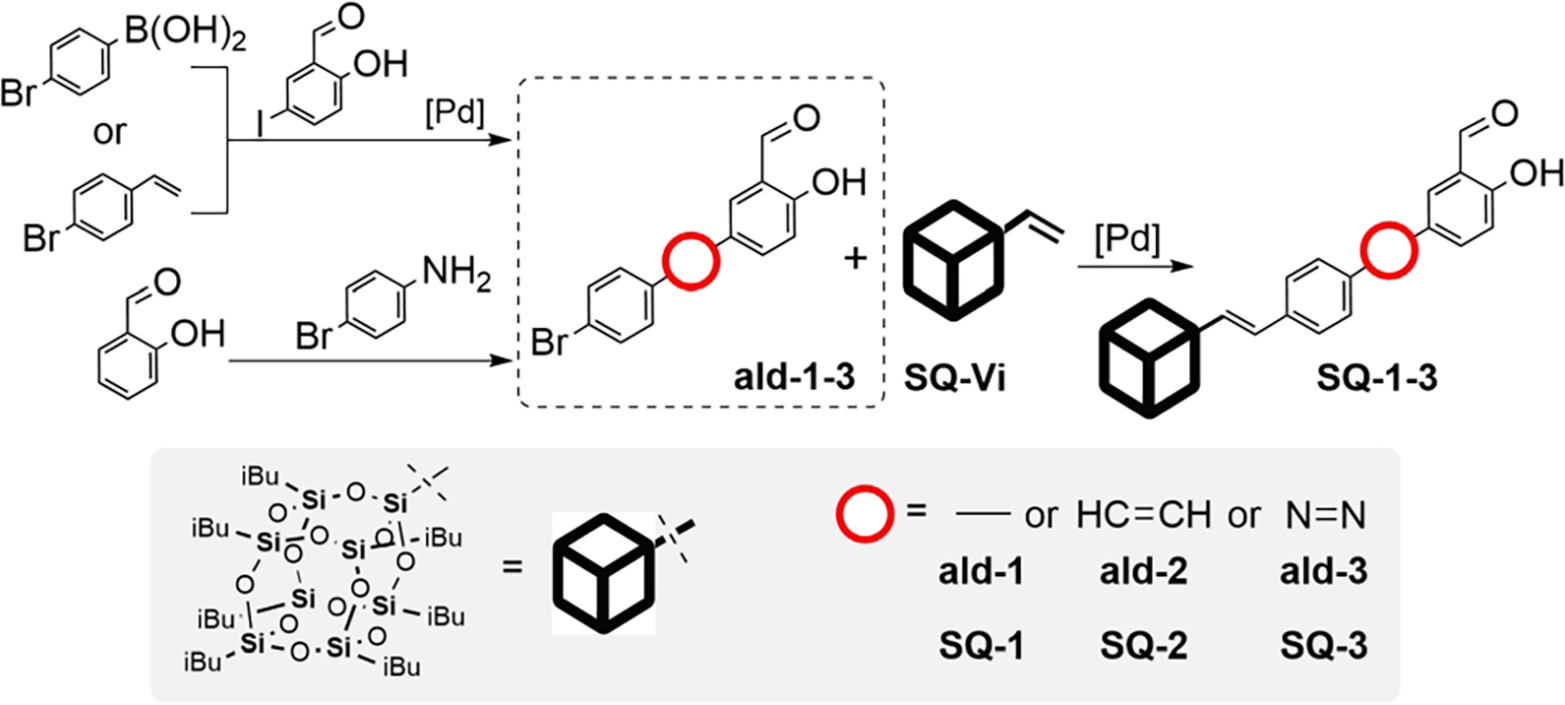
Reaction Synthetic Path to Gain Silsesquioxane-Aldehyde Derivatives (**SQ-1**, **SQ-2**, **SQ-3**)

All compounds obtained at each step were isolated and characterized via spectroscopic and spectrometric techniques, proving their structures. It should be emphasized that the Heck coupling was regioselective toward −HC=CH– formation which may be manifested by the presence of two doublets in the ^1^H NMR spectra of respective aldehydes combined with the T_8_ cage (**SQ-1**, **SQ-2**, **SQ-3**), i.e. 7.24–7.19 ppm and 6.27–6.16 ppm with *J*_HH_ = 19.1 Hz, revealing the (*E*) geometry of −HC=CH– double bond (Figures SI-7, SI-10 and SI-13 in the Supporting Information). The synthesis of silsesquioxane-salphen-based ligands was based on the Schiff reaction between 1,2-phenylenediamine with corresponding salicylaldehyde-silsesquioxane derivatives (**SQ-1**, **SQ-2**, **SQ-3**) in a molar ratio 1:2, to gain respective products abbreviated as **H**_**2**_**Sal-bisSQ-1**, **H**_**2**_**Sal-bisSQ-2**, **H**_**2**_**Sal-bisSQ-3**.^55^ The reactions were performed in ethanol at 80 °C for 24 h due to the low solubility of the aldehydes attached to the silsesquioxane. Elevated temperature and a longer reaction time enabled the complete conversion of both substrates. A series of three ligands were isolated and characterized via spectroscopic and spectrometric techniques and also subjected to photophysical measurements, which will be described below. The obtained silsesquioxane-salphen-based ligands were found to coordinate the Zn^2+^ ions. The syntheses of these complexes were performed in three different manners. This concept is presented in Scheme 2.

**Scheme 2:**
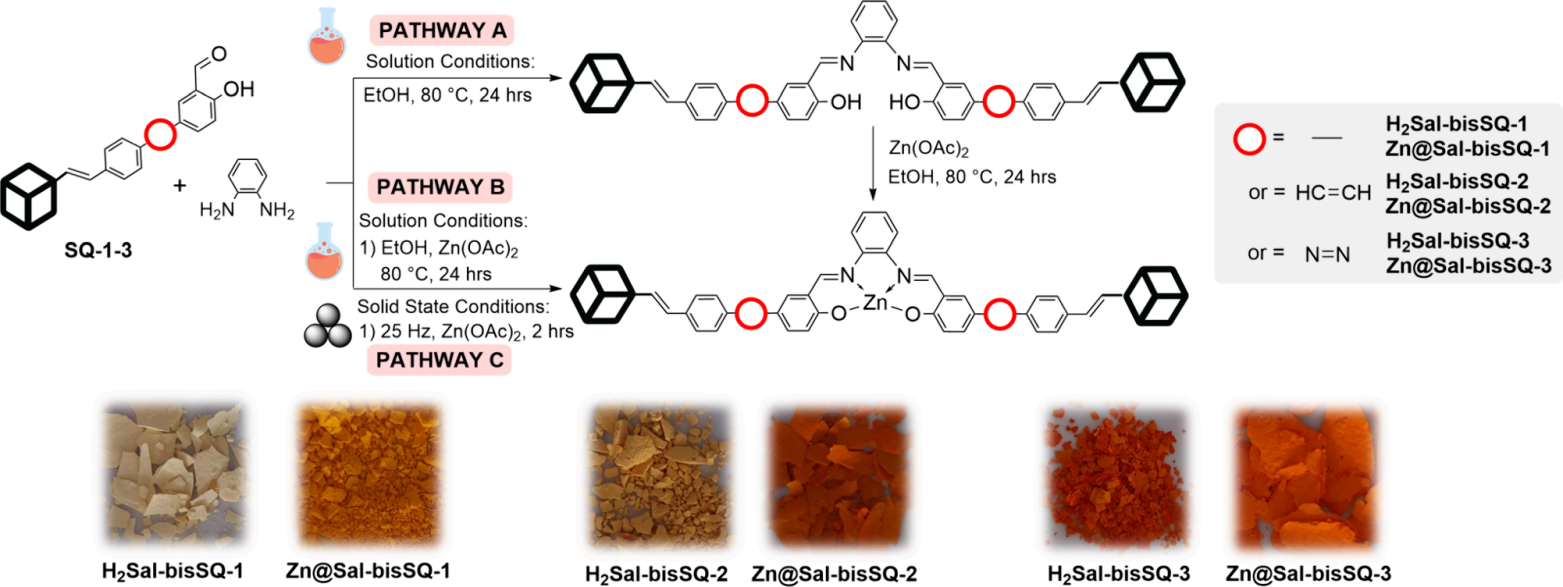
General Reaction Synthetic Path to Gain Silsesquioxane-Salphen-Based Ligands:**H**_**2**_**Sal-bisSQ-1**, **H**_**2**_**Sal-bisSQ-2**, **H**_**2**_**Sal-bisSQ-3**, and Respective Coordination Systems with Zn^2+^: **Zn@Sal-bisSQ-1**, **Zn@Sal-bisSQ-2**, **Zn@Sal-bisSQ-3**

*PATHWAY A*. This route aimed to obtain bare ligands for use as references in a further photophysical analysis. The compounds **H**_**2**_**Sal-bisSQ-1**, **H**_**2**_**Sal-bisSQ-2** or **H**_**2**_**Sal-bisSQ-3** were synthesized *via* a standard Schiff reaction between 1,2-phenylenediamine and the corresponding silsesquioxane-aldehyde derivatives (**SQ-1**, **SQ-2**, **SQ-3**). This resulted in the formation of two imine units per ligand molecule **H**_**2**_**Sal-bisSQ-1**, **H**_**2**_**Sal-bisSQ-2** or **H**_**2**_**Sal-bisSQ-3** (yields: 47–70%) differing in the arene linker. Salen complexes are typically synthesized either by preforming the salen ligand before metal complexation or using an *in situ* approach with a flexible ligand.^42^ The latter was tested for **Zn@Sal-bisSQ-1** but yielded only about 25%, prompting the use of an alternative method. Recently, template-based synthesis, where the metal directs the Schiff reaction, has gained significant interest.^56,57^

*PATHWAY B*. The template approach was verified, and respectively synthesized silsesquioxane-aldehyde derivatives (**SQ-1** or **SQ-2** or **SQ-3**) were used with 1,2-phenylenediamine and Zn(OAc)_2_ in a 2:1:1 ratio, proceeding the reaction in ethanol under an Ar atmosphere at 80 °C for 24 h. The resulting precipitates of products **Zn@Sal-bisSQ-1–3** were washed with EtOH and analyzed and resulted in 76–86% yields. The template protocol may be susceptible to additional effects different than salen composition, like in the case of selected lanthanide^III^ ions with the possibility for intramolecular hydrogen transfer to occur.^58^ It was not observed here.

*PATHWAY C*. Mechanochemistry has gained significant attention for its economy and its accordance with green chemistry procedures. So far, there are a limited amount of reports on the effective route for the sulfa-Michael addition of mercapto-substituted silsesquioxane and α,β-unsaturated aldehydes^59^ and also for Al- and In-salen complexes formation.^60^ This possibility was also verified here, in terms of template complex formation using respective **SQ-1**−**SQ-3**, 1,2-phenylenediamine and Zn(OAc)_2_ in a 2:1:1 ratio. The reaction protocol used the ball-milling handling Liquid-Assisted Grinding (LAG) for 2 h, with frequency 25 Hz.^61^ The formed precipitate was washed with EtOH, and dried under a vacuum. Resulting products: **Zn@Sal-bisSQ-1**, **Zn@Sal-bisSQ-2** and **Zn@Sal-bisSQ-3** were obtained in 90–94% yield.

All products were isolated and characterized by spectroscopic and spectrometric analysis, proving their structures Data are available in the Supporting Information. ^1^H NMR analyses of, e.g. **H**_**2**_**Sal-bisSQ-1** and **Zn@Sal-bisSQ-1** in CDCl_3_ or CD_2_Cl_2_ for their comparison with the literature reports of respectful organic analogues was not possible, especially for the complexes as they were barely soluble in the noncoordinating solvents, e.g. DCM. A significant peak broadening in CD_2_Cl_2_ may be noted that does not allow its quantitative evaluation (Figure 2a). However, it was interesting to find that a small addition of THF, ca. 10% enabled obtaining a clear ^1^H NMR spectrum (Figure 2b). The ^1^H NMR spectra in THF-*d*_8_ exhibited sharp resonance lines with the expected multiplicity, supporting the structure of obtained complex **Zn@Sal-bisSQ-1**. The stacked ^1^H NMR spectra of bare ligand **H**_**2**_**Sal-bisSQ-1** and **Zn@Sal-bisSQ-1** performed in THF-*d*_8_ reveal the absence of −OH protons at 12.94 ppm along with a peak at 8.90 ppm deriving from imine hydrogen −N=CH–. This signal was insignificantly shifted to 9.07 ppm for **Zn@Sal-bisSQ-1** (Figure 2c and 2d), similarly as for the respective analogous complex [*N*,*N*′-bis(salicylidene)-1,2-phenylenediamine]zinc(II) in DMSO (downfield shift of −N=CH– from δ = 8.95 ppm derived from ligand to δ = 9.00 ppm for complex).^46^ This may suggest that there is a minor electronic impact of the -T_8_ moieties along with the ethenylphenylene linker on the placement of the −N=CH– peaks. Also, it is worth noting that there are no reports on the NMR in THF-*d*_8_ to be compared.

**Figure 2:**
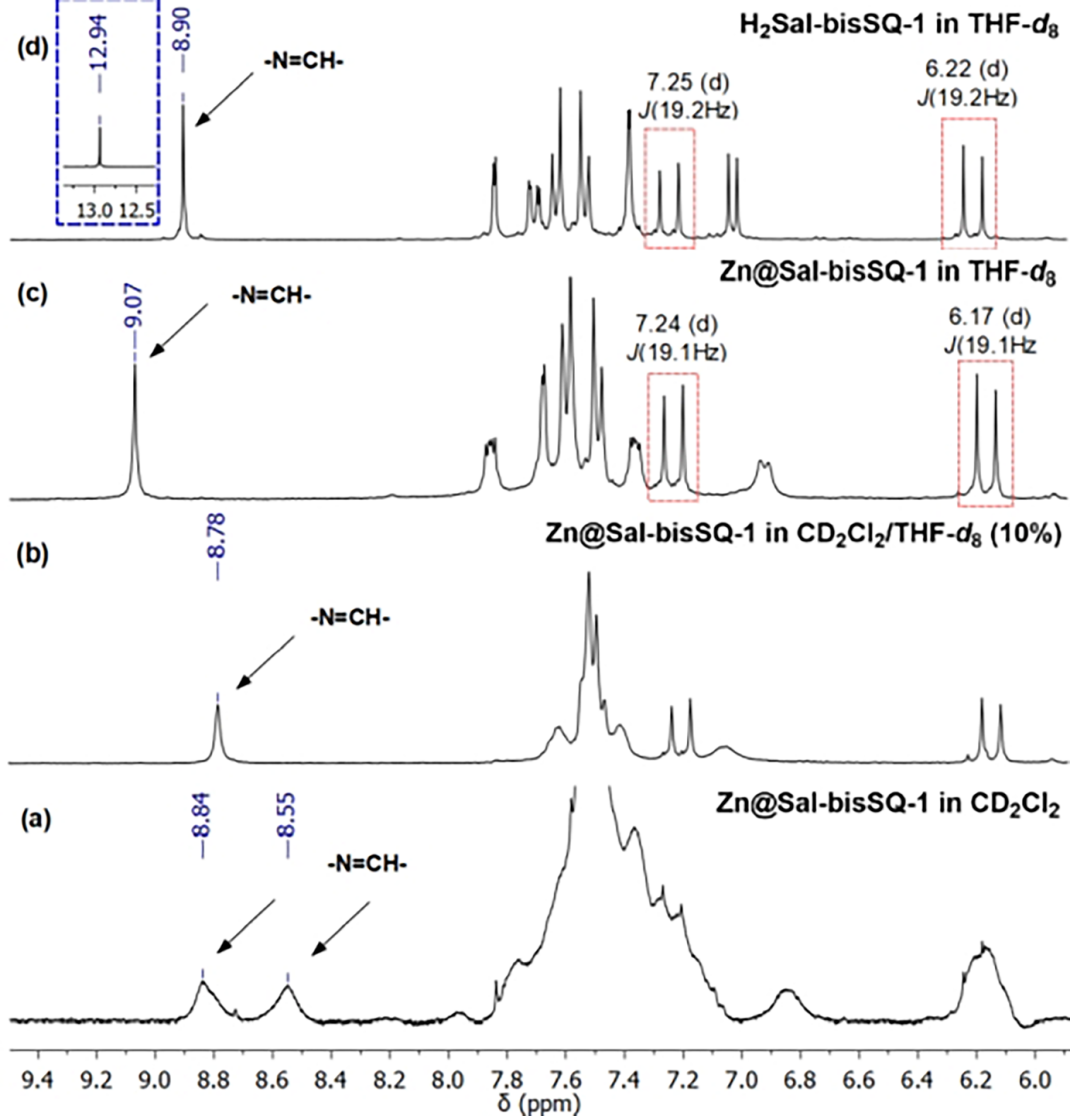
Stacked ^1^H NMR spectra of complex **Zn@Sal-bisSQ-1**: in CD_2_Cl_2_ (a), in CD_2_Cl_2_/THF-*d*_8_ mixture (b), and inTHF-*d*_8_ (c); and ligand **H**_**2**_**Sal-bisSQ-1** in THF-*d*_8_ (d).

Despite the poor solubility of **Zn@Sal-bisSQ-1** in CD_2_Cl_2_ and the resulting broadening of the resonance lines, a comparison of the ^1^H NMR analysis reveals an upfield shift of the imine hydrogen −N=CH– signal (from 9.07 ppm in THF-*d*_8_ to 8.84 and 8.55 ppm in CD_2_Cl_2_), along with a corresponding shift of the aromatic C–H protons. These observations along with the broadening of the signals may suggest the possible formation of dimers (or aggregates) in a staggered constitution with both Zn atoms interacting via the Zn···O axial coordination and as a result achieving the change in the coordination sphere of each Zn^II^ ions to gain square pyramid (Scheme 3). This is in accordance with the respective structures of *N*_2_*O*_2_–Zn complexes.^62^

**Scheme 3:**
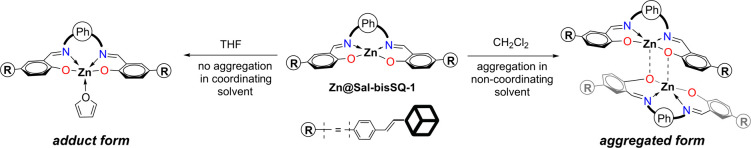
Depiction of Possible Behavior of the Silsesquioxane-Salphen-Based Zinc Compounds in the Presence and Absence of Coordinating Solvent–Formation Monomeric Structure (**Adduct Form**) and Aggregate (**Aggregated Form**)

The strong Lewis acidity of Zn^II^ ions in monomeric **Zn@Sal-bisSQ-1** drives its dimerization for thermodynamic stability.^48^ In the presence of the coordinating solvent THF, acting as a Lewis base, an axial coordination to Zn^II^ occurs, leading to adduct formation and disaggregation of the adduct (Scheme 3, **adduct form**). This aligns with Di Bella et al.’s findings for *N*_2_*O*_2_-ligated Zn complexes.^62^ Notably, dimer/aggregate formation was observed even at very low DCM concentrations (1 × 10^–5^ M), suggesting a strong tendency for aggregation (Scheme 3, **aggregated form**). In the ^1^H NMR spectrum of **Zn@Sal-bisSQ-1** in CD_2_Cl_2_, the doubled imine (−N=CH−) peaks at 8.84 and 8.55 ppm indicate aggregation-induced symmetry changes (Scheme 3). Surprisingly, despite bulky silsesquioxane (T_8_) units causing steric hindrance, aggregation persisted in noncoordinating solvents, reducing solubility.^63^ This contrasts with the typically enhanced solubility of silsesquioxane-containing molecules. Additionally, the **Zn@Sal-bisSQ-1–3** complexes exhibited greater aggregation and lower NMR solubility in noncoordinating solvents than their non-SQ analogues, suggesting that silsesquioxane did not prevent but rather reinforced aggregation. Finally, it was found that the presence of the two -T_8_ units did not affect significantly the placement of the resonance lines of respected ^1^H NMR signals for both, bare ligands as well as the complexes when compared with respective analogues without the silsesquioxanes.

*Photophysical Properties of Silsesquioxane-Salphen-Based Ligands: **H_2_Sal-bisSQ-1**, **H_2_Sal-bisSQ-2**, and **H_2_Sal-bisSQ-3**, and Respective Coordination with Zn^2+^: **Zn@Sal-bisSQ-1**, **Zn@Sal-bisSQ-2**, **Zn@Sal-bisSQ-3***. The absorption and fluorescence spectra of the **Zn@Sal-bisSQ-1** complex in DCM are presented in Figure 3 (spectra for all other compounds are available in SI). The spectra reveal the formation of a new band at longer wavelengths, indicating the presence of aggregates at higher concentrations. The significant spectral shift enables selective excitation of the monomer at 329 nm and the aggregate at 408 nm.^64^ Even when the monomer is exclusively excited at 329 nm, the observed fluorescence predominantly originates from the aggregated form (λ_max_ = 530 nm), with only a much weaker emission detected from the monomer (λ_max_ = 375 nm). This suggests that the aggregate has a significantly higher fluorescence quantum yield. Notably, selective excitation of the aggregate at 408 nm results in a single fluorescence band, peaking at around 530 nm.

**Figure 3:**
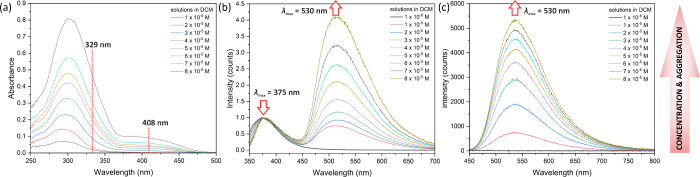
Absorption (a) and emission spectra (with λ_exc_ = 329 nm (b) and with λ_exc_ = 408 nm (c)) of **Zn@Sal-bisSQ-1** in DCM.

These observations were further validated using time-resolved fluorescence spectroscopy (TCSPC technique; details can be found in the SI). As shown in Figure SI-P11 (in the SI), excitation at 329 nm results in two distinct fluorescence lifetimes, fitted to τ_1_ = 0.77 ns and τ_2_ = 4.10 ns, corresponding to the monomer and aggregate of the **Zn@Sal-bisSQ-1** complex, respectively. A similar experiment conducted with λ_exc_ = 408 nm (selective excitation of the aggregate) yielded a single lifetime of 4.10 ns. Additionally, the fluorescence quantum yields for all the ligands and complexes are summarized in Table 1 (based on reference method^65^). Interestingly, no fluorescence was detected for ligand **H**_**2**_**Sal-bisSQ-3** or its complex **Zn@Sal-bisSQ-3**. Furthermore, no spectral evidence of aggregation was observed in the fluorescence spectra of these compounds. The rationale behind this behavior will be discussed later in this section.

**Table 1:** Comparison of the Photophysical Properties of the Complex Aggregates and Their Respective Ligands (in DCM)

Compound	λ_abs_ (nm)	λ_Flu_ (nm)	τ_s_ (ns)	Φ_Flu_	*Φ*_*Δ*_
**H**_**2**_**Sal-bisSQ-1**	275, 315	-	1.53, 3.89^a^	0.005	-
**Zn@Sal-bisSQ-1**	310, 410	375, 530	0.77, 4.10^a^	0.063	0.17
**H**_**2**_**Sal-bisSQ-2**	338	-	0.75	0.010	-
**Zn@Sal-bisSQ-2**	348, 430	400, 585	0.69, 4.08^a^	0.020	0.04
**H**_**2**_**Sal-bisSQ-3**	375, 530	-	0.08	<0.002	-
**Zn@Sal-bisSQ-3**	405	-	0.01	<0.002	0

a See text for a detailed explanation of a double exponential decay.

Given the moderately low fluorescence yields for all of the compounds, we decided to investigate other energy dissipation channels, such as the formation of excited triplet states. Figures 4a and 4b show results obtained using the Laser Flash Photolysis technique with 410 nm excitation of the **Zn@Sal-bisSQ-1** complex in argon-saturated DCM. In the absence of oxygen, a distinct triplet–triplet absorption spectrum is observed with a triplet lifetime of approximately 15 μs. The kinetic trace observed at 470 nm is efficiently quenched by O_2_, confirming the triplet nature of the excited state. Additionally, the **Zn@Sal-bisSQ-1** aggregated complex was found to generate the triplet state more efficiently than any other compound studied, making it a promising candidate for singlet oxygen sensitization in potential therapeutic applications (e.g., in PDT). As shown in Figure 4c, the complex in air-equilibrated DCM solution emits in the near-infrared region, peaking at around 1270 nm. This emission is characteristic of singlet oxygen phosphorescence (^1^O_2_), an assignment further supported by the observed lifetime of approximately 90 μs (Figure 4d), which is consistent with the previously reported singlet oxygen lifetime of 88–91 μs.^66,67^

**Figure 4:**
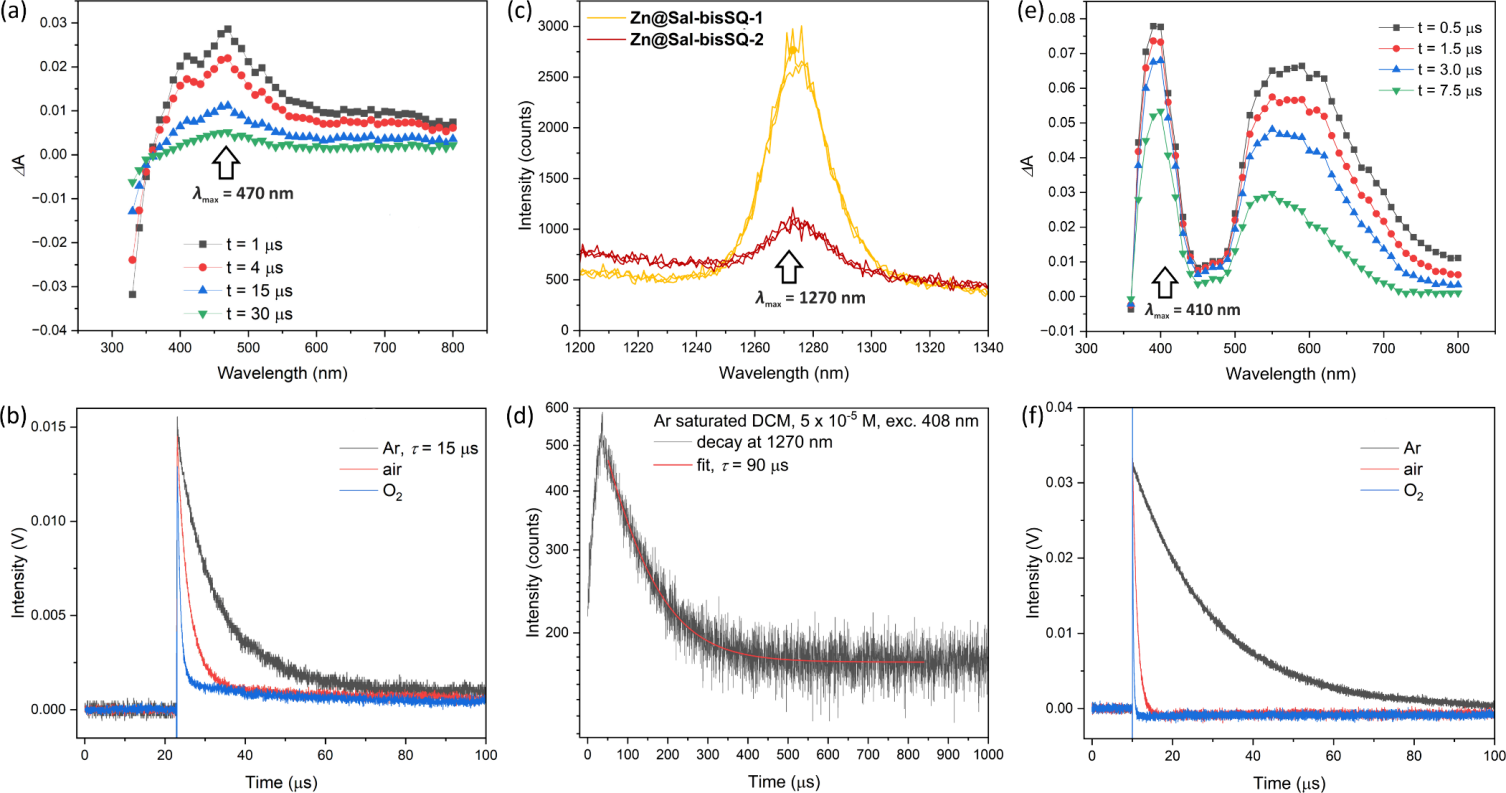
Nanosecond laser flash photolysis (λ_exc_ = 410 nm) of **Zn@Sal-bisSQ-1** in DCM: (a) transient absorption spectra after different time delays following the 410 nm laser pulse; (b) kinetic traces collected at 470 nm in the absence and presence of air and oxygen. Sample concentration: 5 × 10^–5^ M. (c) Singlet oxygen phosphorescence spectra obtained for air-equilibrated DCM solutions of **Zn@Sal-bisSQ-1** and **Zn@Sal-bisSQ-2**; and (d) decay trace of **Zn@Sal-bisSQ-1** collected at λ_max_ = 1270 nm (λ_exc_ = 408) in DCM. Samples concentration: 5 × 10^–5^ M. (e) Nanosecond laser flash photolysis (λ_exc_ = 435 nm) of **Zn@Sal-bisSQ-1** in THF: transient absorption spectra after different time delays following the 435 nm laser pulse; (f) kinetic traces collected at 410 nm in the absence and presence of air and oxygen. Sample concentration: 3 × 10^–5^ M.

The singlet oxygen quantum yields for the **Zn@Sal-bisSQ-1** and **Zn@Sal-bisSQ-2** complexes were calculated using Eosin Y as a reference (with a ^1^O_2_ QY of 0.64),^66^ yielding values of 0.17 and 0.04, respectively. These results are in good agreement with the triplet yields obtained from nanosecond transient absorption measurements, indicating a much higher yield of singlet oxygen formation for the **Zn@Sal-bisSQ-1** complex.

To investigate the solvent dependence of the photophysical properties of **Zn@Sal-bisSQ-1**, analogous measurements were conducted in a THF solution. The comparison of the results obtained in DCM and THF is depicted in the stacked absorbance/emission spectra in Figure SI-P8–P9 (in the SI). Interestingly, in THF, the absorbance spectrum consistently displayed a band in the 400–500 nm range, regardless of concentration. This suggests that dimer or aggregate formation is not readily apparent in the absorption spectra (see Figure SI-P7 in the Supporting Information). Additionally, the fluorescence spectra showed a linear increase in fluorescence intensity with increasing concentration of the complex in THF in the entire spectral region (Figure SI-P10 in the Supporting Information), with only minor signs of aggregation observed in the 400–500 nm region. This trend was also reflected in time-resolved fluorescence measurements, where a single lifetime, corresponding to the monomeric form of the complex ( = 0.80 ns), was observed (Table 2).

**Table 2:** Time-Resolved Fluorescence Measurement Data for **Zn@Sal-bisSQ-1** in DCM and THF

**Zn@Sal-bis-SQ-1**	λ_exc_ (nm)	τ_s_ (ns)	Contribution (%)
in DCM	329	0.77	81
		4.30	19
	408	0.70	<1
		4.20	>99
in THF	329	3.20	<1
		0.80	>99
	440	2.90	1.4
		0.80	98.6

Conversely, despite the lack of aggregation of **Zn@Sal-bisSQ-1** in THF, the monomeric complex demonstrates efficient triplet formation, as shown in Figure 4d. The triplet nature of this spectral feature was further confirmed by its efficient quenching in the presence of air and oxygen (Figure 4e) and by its ability to generate singlet oxygen in THF. This was validated by the presence of an emission peak at 1270 nm and an experimentally measured phosphorescence lifetime of 21 μs (see Figure SI-P13 in the Supporting Information).

The minor contribution of the longer lifetime (*ca*. 1% in the range of apparatus precision) could be attributed to the fluorescence of the aggregate. However, this contribution is minimal, especially when compared to the behavior in DCM solutions described earlier, and can therefore be considered negligible. The experimentally measured fluorescence lifetime of the monomeric form in THF (τ = 0.80 ns) is in good agreement with the corresponding lifetime in DCM (τ = 0.77 ns) (seeFigures SI-P11 and SI-P12 in the Supporting Information).

A comparison of the results from both solvents, along with NMR and photophysical measurements, leads to similar conclusions: **Zn@Sal-bisSQ-1** shows a strong tendency for dimerization and aggregation in DCM, a noncoordinating solvent, whereas in THF, the measurements support the preservation of its monomeric structure. Interestingly, for **Zn@Sal-bisSQ-3** (as well as the corresponding **H**_**2**_**Sal-bisSQ-3** ligand), neither triplet formation nor singlet oxygen sensitization was observed. The absence of fluorescence and triplet formation in these compounds suggests the presence of alternative energy dissipation channels. To investigate the excited-state dynamics of these compounds, we applied femtosecond transient absorption spectroscopy. In general, the aggregates of the studied complexes exhibit significantly higher fluorescence and singlet oxygen yields compared with their monomeric counterparts. This is likely since aggregation reduces the possibility of rotational motion within the ligands’ structures, thereby stabilizing the structure and blocking rotational energy dissipation. Such geometrically more rigid structures are known to enhance the fluorescence yields. The lack of aggregation in **Zn@Sal-bisSQ-3**, as seen in the absorption and fluorescence spectra (see SI), is probably due to its molecular structure, which is less prone to stacking compared to the other two complexes.

As a result, this ligand/complex deactivates through rotational motions and/or *cis*-*trans* isomerization with these fast intramolecular processes effectively competing with fluorescence. The excited singlet state lifetime of **Zn@Sal-bisSQ-3** was determined to be approximately 10 ps corresponding to a rate constant of 10^11^ s^–1^ (see Figure 5), an ultrafast kinetic process that is 2 orders of magnitude faster than the typical fluorescence rate of approximately 10^9^ s^–1^.

**Figure 5:**
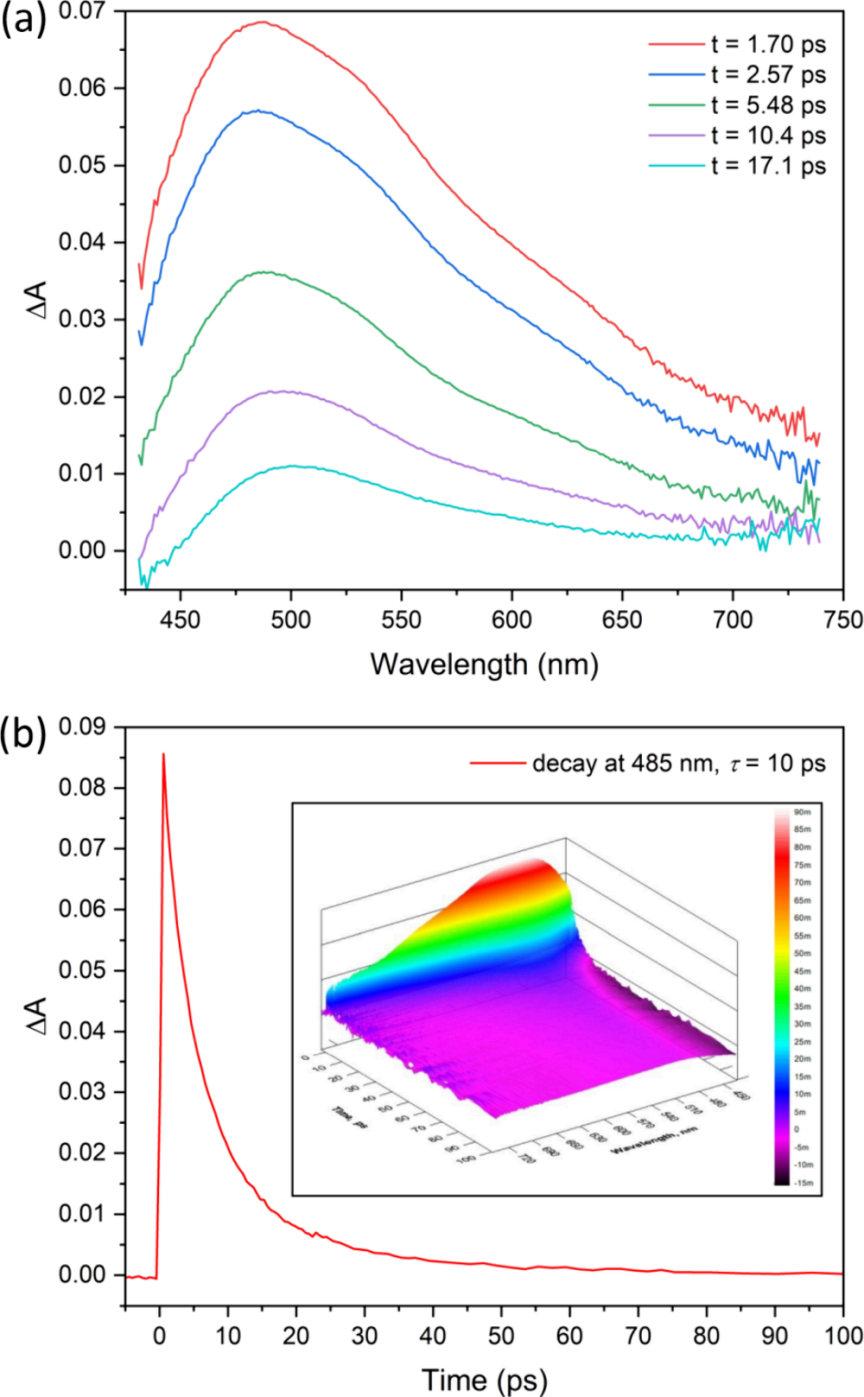
(a) Femtosecond transient absorption spectra registered at time delays from 1.7 to 17.1 ps for DCM solutions of **Zn@Sal-bisSQ-3**; (b) kinetic time profile collected at 485 nm (the fitted lifetime is 10 ps). Sample concentration: 5 × 10^–5^ M.

In contrast, the lack of aggregation in **Zn@Sal-bisSQ-3**, likely due to its complex molecular geometry, results in an extremely short excited-state lifetime (10 ps, as shown in Figure 5). These insights were made possible only through the use of advanced, time-resolved spectroscopic methods applied in this study.

In summary, we have designed an efficient synthetic pathway (in solution combined with a mechanochemical approach) to obtain Zn-salphen-based molecules with SQ-T_8_ units at their peripheries, resulting in the formation of dumbbell-like structures. Each of these compounds features the presence of κ^4^*-N*_*2*_*O*_*2*_*-* donating atoms to ligate the Zn^II^ ion. A strong dimerization of these architectures was observed in noncoordinating DCM facilitated by Zn···O axial coordination from two molecules, forming a square pyramid. On the other hand, THF enables disaggregation of these dimers, leading to the formation of an adduct–as a Lewis base. A series of spectroscopic techniques, including UV–vis absorption, fluorescence spectroscopy, nanosecond and femtosecond transient absorption, time-correlated single-photon counting, and singlet oxygen measurements, were used to investigate the photophysical properties of Zn complexes. These studies also explored their aggregated forms in DCM solutions. Both steady-state and time-resolved experimental techniques were employed to carry out these photophysical studies. Absorption and fluorescence spectra reveal the formation of aggregates in DCM solutions for **Zn@Sal-bisSQ-1** and **Zn@Sal-bisSQ-2**, while no such behavior was observed for **Zn@Sal-bisSQ-3**. The research highlights the significant influence of the ligand structure on the photophysical properties of the complex aggregates. Flatter structures exhibit moderately high fluorescence yields (6.3% for **Zn@Sal-bisSQ-1**) and singlet oxygen generation (17% for **Zn@Sal-bisSQ-1**). In contrast, the **Zn@Sal-bisSQ-3** complex decays on an extremely short time scale (*k* = 10^11^ s^–1^) due to its ability to dissipate energy through fast intramolecular motions. Similar experimental studies carried out in THF showed no or negligible aggregation and therefore lower fluorescence quantum yields. Low triplet yields result in consequently inefficient singlet oxygen generation. This contrasting behavior can be rationalized by the contribution of THF in the complexation resulting in steric hindrance preventing the aggregation process.

This study underscores the importance of understanding these effects to develop more effective fluorescence probes, delivery systems, and photodynamic therapy (PDT) drugs. Future investigations should focus on designing structures with higher fluorescence quantum yields and longer triplet state lifetimes, which would enhance the complex’s ability to generate singlet oxygen more efficiently. The formation of aggregates, as observed for **Zn@Sal-bisSQ-1** and **Zn@Sal-bisSQ-2** partially restricts rotational motion within the molecule, thereby enabling alternative deactivation channels such as fluorescence and intersystem crossing, which ultimately lead to efficient singlet oxygen generation. The ability to reliably produce these hybrid compounds opens new possibilities for tailoring the chemical and physical properties of these materials for various applications, including coordination chemistry and photodynamic therapy. The developed methodology provides a solid foundation for future work aimed at exploring and optimizing the ligation properties and photophysical characteristics of similar coordination compounds.

## Supporting Information Available

The authors have cited additional references within the Supporting Information.^61,66,68,69^ The Supporting Information contains all information describing materials, synthetic procedures, measurements, and spectroscopic analysis of all isolated products (Figures SI-1–SI-33) along with the photochemistry data (Figures SI-P1–SI-P22) (PDF)
