# Supplementary material for: Excited State Dynamics Govern Emission Properties of Unique Silsesquioxane-Salphen-Based Zinc Compounds
Source: J Phys Chem Lett. 2025 Mar 3;16(10):2571–80. doi: 10.1021/acs.jpclett.4c03406 (PMC11912527; doi:10.1021/acs.jpclett.4c03406)
Supplement: Supplementary file 1 — jz4c03406_si_001.pdf [file jz4c03406_si_001.pdf]

Supporting Information

for

Excited state dynamics govern emission properties of unique silsesquioxane-salphen-based zinc compounds

Joanna Szymkowiak,[1] Tomasz Pędziński,[1,2] Beata Dudziec*[,1,2]

[1]*Center for Advanced Technologies, Adam Mickiewicz University in Poznan, Uniwersytetu Poznanskiego 10, 61-614 Poznan, Poland*

[2]*Faculty of Chemistry, Adam Mickiewicz University in Poznan, Uniwersytetu Poznanskiego 8, 61-614 Poznan, Poland*

*Corresponding author: beata.dudziec@gmail.com

Table of Contents:

1. General remarks………………………………………………………………………...….… S-2
 1.1. Measurements……………………………………………………….………..……..... S-2
2. Synthesis details………………………………………….……………….……….….…...… S-5
 2.1. Procedures for the synthesis of aldehydes **ald-1–3**…………………………………………..….. S-5
 2.2. General procedure for the synthesis of aldehydes **SQ-1–3** *via* Heck coupling reaction……..….. S-7
 2.3. General procedure for the synthesis of salphenes **H$_2$Sal-bisSQ-1–3** (Path A – in solution)…..... S-9
 2.4. General procedure for the synthesis of complexes **Zn@Sal-bisSQ-1–3** (Path B – in solution)... S-11
 2.5. General procedure for the synthesis of complexes **Zn@Sal-bisSQ-1–3** (Path C – in ball mill)... S-11
3. Copies of NMR spectra……………………………………………………………..…………… S-13
4. Photochemistry data ………………………………………………………………...…... S-30

1. General remarks

All manipulations, except work-up and purification of final products, were conducted under argon atmosphere using standard Schlenk techniques. Tetrahydrofuran was purified by distillation from sodium with benzophenone as indicator; toluene was distilled from calcium hydride; *N*,*N*-dicyclohexylmethylamine was distilled from calcium hydride using distillation under reduced pressure technique. Other solvents and reagents were used as received from commercial suppliers. Column chromatography was performed on Merck Kieselgel type 60 (250 – 400 mesh). Merck Kieselgel type 60F254 analytical plates were employed for TLC. All known compounds were identified by spectroscopic comparison with authentic samples.

Solid-state syntheses were carried out by using ball milling method and the Liquid-Assisted Grinding (LAG) protocol[61] in Retsch MM400 mixer mill (stainless steel milling jar with a volume 1.5 mL, and with one stainless steel milling ball with a diameter 5 mm).

1.1. Measurements

Nuclear Magnetic Resonance (NMR) spectra of synthesized compounds were recorded on Bruker Avance 600 MHz operating at 600 MHz for ^{1}H NMR, at 151 MHz for ^{13}C{^{1}H} NMR and at 119 MHz for ^{29}Si{^{1}H} NMR; or Bruker Ascend™ 400 MHz operating at 400 MHz for ^{1}H NMR, at 101 MHz for ^{13}C{^{1}H} NMR, at 79 MHz for ^{29}Si{^{1}H} NMR; or Bruker Ascend™ 300 MHz operating at 300 MHz for ^{1}H NMR, at 75 MHz for ^{13}C{^{1}H} NMR. Chemical shifts (δ) are reported in ppm and coupling constants (J) are given in Hz. Reference values for residual solvents were taken as δ = 7.26 ($CDCl_3$) for ^{1}H NMR; δ = 77.16 ($CDCl_3$) for ^{13}C{^{1}H} NMR; δ = 3.58, 1.73 (THF-d_8) for ^{1}H NMR; δ = 67.57, 25.37 (THF-d_8) for ^{13}C{^{1}H} NMR; δ = 150.35, 135.91, 123.87 (pyridine-d_5) for ^{13}C{^{1}H} NMR. Spectra were measured at 298 K (samples in $CDCl_3$ and THF-d_8); or at 328 K (sample in pyridine-d_5).

Fourier transform infrared (FT-IR) spectra were recorded on a Nicolet iS5 Thermo Scientific spectrophotometer equipped with a diamond ATR unit and reported as wave numbers $\tilde{\nu}$ in cm^{-1}. In all cases, 16 scans at resolution of 2 cm^{-1} were collected, to record spectra in a range of 4000 – 650 cm^{-1}.

High-resolution mass spectrometry (HRMS) measurements were performed using Synapt G2-Si mass spectrometer (Waters Corp., Milford, MA, USA) equipped with an ESI source and quadrupole-Time-of-Flight mass analyzer. The mass spectrometer was operated in the negative ion detection mode. The optimized source parameters were: capillary voltage 3.0 kV, cone voltage 30 V, source temperature 120 °C, desolvation gas (nitrogen) flow rate 600 L/h with the temperature 350 °C, nebulizer gas pressure 6.5 bar. To ensure accurate mass measurements, data were collected in centroid mode, and mass was corrected during acquisition using leucine encephalin solution as an external reference, Lock-SprayTM, (Waters). The results of the measurements were processed using the MassLynx 4.1 software (Waters).

MALDI-TOF mass spectra were measured on an on a Axima Performance (Shimadzu, Japan) instrument equipped with nitrogen laser (337 nm). The pulsed extraction ion source accelerated the ions to a kinetic energy of 20 keV. All data have been obtained in a positive-ion reflection mode. The energy of the laser beam was set slightly above threshold level. The matrix (dithranol – DT) was dissolved at a concentration of 20 mg/mL in tetrahydrofurane (THF). The matrix solution was spotted onto the target and dried in air. In the next step sample solution (2 mg/mL) was deposited onto the matrix spot and dried in air. Mass spectra were accumulated from at least 200 laser shots and processed by Biotech Launchpad ver. 2.9.1 program (Shimadzu).

UV-vis and **Fluorescence spectra** were collected using HORIBA Duetta Scientific, a 2-in-1 fluorescence and absorbance spectrophotometer at room temperature in solutions and with the use of a quartz cell of optical lengths 0.1 cm (for absorbance measurements) or 1 cm (for fluorescence measurements). The concentration of analytes ranged from 1.0×10^{-6} to 1.0×10^{-4} mol L^{-1} .

Absorbance spectra were measured in 250 – 700 nm of spectra range with using: pure solvent blank correction, 2 nm band pass, 2 nm step increment, 0.2 s integration time.

Fluorescence spectra were measured with using: 3 nm excitation band pass, 3 nm emission band pass, 0.2 s integration time, 10 detector accumulations, 0.5 nm emission increment, emission range 340 – 750 nm for excitation wavelength 329 nm, in emission range 420 – 800 nm for excitation wavelength 408 nm, and emission range 450 – 800 nm for excitation wavelength 440 nm.

Fluorescence quantum yield (ϕ_f^x) was determined using a reference standard method. Acridinium cation has been used as standard in experiments to determine fluorescence quantum yield.[68] The following equation have been applied to determine the quantum yield of fluorescence (Eq.1):

$$\boldsymbol{\phi_f^x = \frac{S_x A_{st}^{\lambda} n_x^2}{S_{st} A_x^{\lambda} n_{st}^2} \phi_f^{st}} \qquad \text{(Eq.1)}$$

Where: ϕ_f^x – fluorescence quantum yield of a sample x; S_x, S_{st} – integrated fluorescence intensity (area under the spectrum) for sample x and standard sample, respectively; $A_x^{\lambda}, A_{st}^{\lambda}$ – absorbance at the excitation wavelength (λ) for sample x and standard sample, respectively; ϕ_f^{st} – fluorescence quantum yield of the standard sample (ϕ_f^{st} = 0.86 in water); n_x, n_{st} – refractive index for sample x solvent's and standard sample solvent's, respectively.

Fluorescence lifetimes were measured using a Fluorescence Lifetime Spectrometer (FluoTime 300, PicoQuant) equipped with a time-correlated single-photon counting (TCSPC) detection system. Emission decay lifetimes were acquired using 408 nm and 440 nm diode lasers as excitation sources. The instrument response function (IRF) was obtained using a Ludox solution (colloidal silica).

Quantum yields of singlet oxygen production (Φ_Δ) generated by the complex aggregates were calculated based on steady-state measurements. These measurements were recorded on the PicoQuant FluoTime 300 fluorescence lifetime spectrometer, using an H10330B-45 NIR-PMT module, which is sensitive in the 950 to 1400 nm NIR range. Excitation was performed with a picosecond laser diode (LDH-440 nm, PicoQuant) at 440 nm, using Eosin Y (EY) as the standard (Φ_Δ = 0.64, λ_{exc} = 440 nm).[66] Air-equilibrated solutions of the complexes were optically matched to a standard reference solution at the excitation wavelength (440 nm). The total area under the emission spectrum was calculated separately for each solution. The quantum yield of singlet oxygen (Φ_Δ) was then determined by comparing the total area under the emission spectra of the complex and the standard.

For **time-resolved measurements**, samples were excited using a high repetition rate 40 MHz picosecond laser diode (LDH-440 nm, PicoQuant). Decay traces at λ = 1270 nm were collected using a "burst mode," where the sample is first excited with multiple laser pulses to build up the population of singlet oxygen, followed by monitoring the decay over a 60 μs time window.

Femtosecond transient absorption measurements **(fsTA)** were conducted using a Solstice Ti:Sapphire regenerative amplifier (Spectra Physics) paired with an optical detection system provided by Ultrafast Systems (Helios). The source for both pump and probe pulses was the primary emission at 800 nm. This primary emission was split into two beams: 95% for the pump and 5% for the probe. The pump beam was directed through the TOPAS-Prime automatic optical parametric amplifier (Spectra Physics) to achieve the desired excitation wavelength within the 290 – 2600 nm range. The probe beam was routed to a fsTA pump-probe Helios spectrometer (Ultrafast Systems LLC), equipped with an optical delay line that allowed delays of up to 3 ns between the pump and probe. A white-light continuum, generated from 5% of the primary beam by passing it through a sapphire or calcium fluoride crystal, was used for transient detection.

The setup for **nanosecond Laser Flash Photolysis (LFP)** experiments and the data acquisition system have been previously described in details.[69] LFP experiments employed an Nd:YAG laser (355 nm, 5 mJ, 7 – 9 ns) for excitation. Transient kinetics were recorded at individual wavelengths using a step-scan method, with a step distance of 10 nm over the 300 – 700 nm range, averaging 8 – 10 pulses per measurement. Solutions for LFP were deoxygenated with high-purity argon for 15 minutes prior to measurements.

2. Synthesis details

2.1. Procedures for the synthesis of aldehydes ald-1–3

ald-1: 4'-bromo-4-hydroxy-[1,1'-biphenyl]-3-carbaldehyde

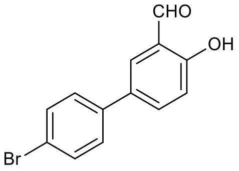

5-Iodosalicylaldehyde (1.33 equiv, 0.744 g, 3 mmol), 4-bromophenylboronic acid (1 equiv, 0.452 g, 2.25 mmol), K_2CO_3 (4 equiv, 1.244 g, 9 mmol), and $Pd(PPh_3)_4$ (0.150 g, 0.13 mmol) were stirred in toluene (15 mL) under an argon atmosphere for 15 min at room temperature. Next, a mixture of degassed $EtOH/H_2O$ (15 mL, 1:1 v/v) was added in one portion *via* syringe; and the reaction mixture was stirred for 72 hrs at 75 °C. After cooling to room temperature, the mixture was quenched by the addition of 10% aqueous solution of HCl (30 mL), followed by extraction with DCM (3 × 20 mL). The organic extracts were combined, washed with brine, and dried over anhydrous Na_2SO_4. The solvents were evaporated under reduced pressure and the obtained crude product was purified by column chromatography on silica gel using hexane/DCM mixture (2:1 v/v) as eluent. After workup and purification, the desired product was obtained as a yellow solid, 0.512 g, with 82% isolated yield.

^{1}H NMR (300 MHz, $CDCl_3$) δ 11.02 (s, 1H), 9.95 (s, 1H), 7.76 – 7.66 (m, 2H), 7.56 (d, J = 8.4 Hz, 2H), 7.40 (d, J = 8.4 Hz, 2H), 7.06 (d, J = 9.3 Hz, 1H).

^{13}C{^{1}H} NMR (75 MHz, $CDCl_3$) δ 196.58, 161.28, 138.31, 135.49, 132.17, 132.10, 131.74, 128.23, 121.70, 120.81, 118.43.

FT-IR (ATR) $\tilde{\nu}$ 3058, 2856, 1682, 1649, 1590, 1472, 1391, 1370, 1346, 1322, 1294, 1267, 1225, 1174, 1070, 1005, 906, 829, 813, 771, 756, 741, 710, 688, 631, 613, 584, 513, 464.

ald-2: (E)-5-(4-bromostyryl)-2-hydroxybenzaldehyde

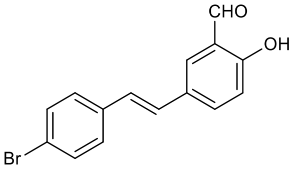

4-Bromostyrene (1 equiv, 0.10 g, 0.07 mL, $5.46 \cdot 10^{-4}$ mol), 5-iodosalicylaldehyde (1 equiv, 0.1354 g, $5.46 \cdot 10^{-4}$ mol), and Pd[P(*t*-Bu)$_3$]$_2$ (0.06 equiv, 16.7 mg, $3.276 \cdot 10^{-5}$ mol) were dissolved in toluene (15 mL), and then *N*,*N*-dicyclohexylmethylamine (4 equiv, 0.43 g, 0.47 mL, $2.184 \cdot 10^{-3}$ mol) was added dropwise *via* syringe. The reaction mixture was stirred for 72 hrs at 75 °C. After cooling to room temperature, the mixture was quenched by the addition of 10% aqueous solution of HCl (10 mL), followed by extraction with DCM (3 × 10 mL). The organic extracts were combined, washed with brine, and dried over anhydrous Na_2SO_4. The solvents were evaporated under reduced pressure and the obtained crude product was purified by column chromatography on silica gel using hexane/DCM mixture (3:1 v/v) as eluent. After workup and purification, the desired product was obtained as a yellow solid, 0.10 g, with 60% isolated yield.

^{1}H NMR (300 MHz, $CDCl_3$) δ 11.03 (s, 1H), 9.94 (s, 1H), 7.71 (dd, J = 8.7, 2.3 Hz, 1H), 7.66 (d, J = 2.3 Hz, 1H), 7.53 – 7.45 (m, 2H), 7.42 – 7.31 (m, 2H), 7.10 – 6.90 (m, 3H).

^{13}C{^{1}H} NMR (101 MHz, $CDCl_3$) δ 196.64, 161.42, 136.08, 134.76, 132.02, 131.79, 129.50, 127.99, 127.46, 126.98, 121.61, 120.75, 118.37.

FT-IR (ATR) $\tilde{\nu}$ 3199, 3061, 3046, 3025, 2980, 2970, 2898, 2869, 1661, 1578, 1487, 1400, 1378, 1291, 1261, 1193, 1151, 1119, 1072, 1008, 970, 949, 937, 906, 831, 810, 770, 738, 699, 652, 571, 499, 482, 449.

ald-3: (E)-5-((4-bromophenyl)diazenyl)-2-hydroxybenzaldehyde

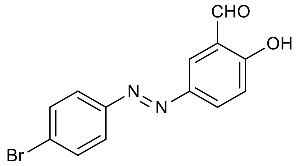

Salicylaldehyde (6.10 g, 0.05 mol) was added to the previously prepared solution of NaOH (2.0 g, 0.05 mol) in water (150 mL). The mixture was cooled to 5 °C using ice bath. [solution A]
4-Bromoaniline (8.60 g, 0.05 mol) was dissolved in a mixture of 35% hydrochloric acid (10 mL) and water (25 mL), and cooled to 0–5 °C. Sodium nitrite (3.79 g, 0.055 mol) was dissolved in water (25 mL) and added to the cold aniline hydrochloride solution. [solution B]
Solution B was added dropwise to solution A; the mixture was allowed to stand in an ice bath for 5 hrs; and then neutralized using 10% HCl aqueous solution. The dark-red precipitate was filtered off, washed with water and dried. The obtained crude product was purified by column chromatography on silica gel using hexane/DCM mixture (1:1 v/v) as eluent. After workup and purification, the desired product was obtained as an orange solid, 1.45 g, with 10% isolated yield.
^{1}H NMR (300 MHz, $CDCl_3$) δ 11.35 (s, 1H), 10.04 (s, 1H), 8.25 – 8.12 (m, 2H), 7.84 – 7.73 (m, 2H), 7.71 – 7.57 (m, 2H), 7.13 (d, J = 8.8 Hz, 1H).
^{13}C{^{1}H} NMR (101 MHz, $CDCl_3$) δ 196.63, 164.15, 151.22, 145.91, 132.56, 130.78, 129.75, 125.57, 124.40, 120.47, 118.85.
FT-IR (ATR) $\tilde{\nu}$ 3186, 2872, 1657, 1618, 1568, 1474, 1393, 1380, 1358, 1278, 1242, 1192, 1153, 1099, 1061, 1008, 952, 904, 844, 828, 768, 714, 659, 579, 505, 447.

2.2. General procedure for the synthesis of aldehydes SQ-1–3 *via* Heck coupling reaction

SQ-Vi (1 equiv, 0.20 g, 2.36·10^{-4} mol), aldehyde **ald-1** or **ald-2** or **ald-3** (1 equiv, 2.36·10^{-4} mol), and Pd[P(*t*-Bu)$_3$]$_2$ (0.06 equiv, 7.2 mg, 1.42·10^{-5} mol) were dissolved in toluene (10 mL), and then *N*,*N*-dicyclohexylmethylamine (4 equiv, 0.18 g, 0.20 mL, 9.44·10^{-4} mol) was added dropwise *via* syringe. The reaction mixture was stirred for 72 hrs at 120 °C. After cooling to room temperature, the mixture was quenched by the addition of 10% aqueous solution of HCl (10 mL), followed by extraction with DCM (3 × 10 mL). The organic extracts were combined, washed with brine, and dried over anhydrous Na_2SO_4. The solvents were evaporated under reduced pressure and the obtained crude product was purified by column chromatography on silica gel using hexane/DCM mixture (2:1 v/v) as eluent. After workup and purification, the desired product was obtained as a solid.

SQ-1

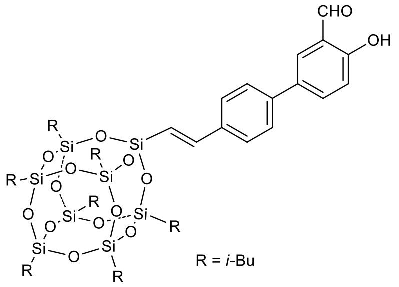

Light yellow solid, 224 mg, 91% isolated yield.

^{1}H NMR (300 MHz, $CDCl_3$) δ 11.03 (s, 1H), 9.99 (s, 1H), 7.83 – 7.74 (m, 2H), 7.62 – 7.50 (m, 4H), 7.24 (d, *J* = 19.1 Hz, 1H), 7.09 (d, *J* = 9.5 Hz, 1H), 6.21 (d, *J* = 19.1 Hz, 1H), 1.92 (dh, *J* = 13.5, 6.8 Hz, 7H), 1.00 (dd, *J* = 6.6, 3.8 Hz, 42H), 0.67 (dd, *J* = 12.8, 7.0 Hz, 14H).

^{13}C{^{1}H} NMR (75 MHz, $CDCl_3$) δ 196.73, 161.27, 147.41, 139.60, 136.97, 135.67, 132.86, 131.85, 127.58, 126.81, 120.90, 119.23, 118.38, 25.89, 24.06, 24.03, 22.69, 22.64.

^{29}Si{^{1}H} NMR (79 MHz, $CDCl_3$) δ -67.36, -67.79, -67.81, -79.96.

FT-IR (ATR) $\tilde{\nu}$ 2952, 2925, 2906, 2868, 1682, 1659, 1607, 1591, 1482, 1464, 1401, 1382, 1365, 1331, 1299, 1265, 1228, 1168, 1081, 989, 954, 908, 837, 740, 557, 472, 429.

HR TOF MS ES$^-$ *m/z* calcd for [M-H]$^-$ $C_{43}H_{73}O_{14}Si_8$ 1037.3154, found 1037.3169.

SQ-2

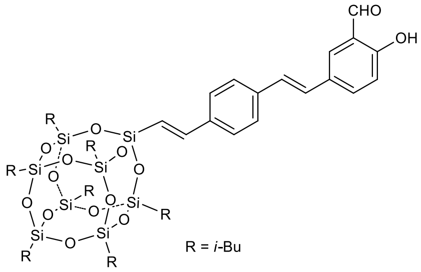

Yellow solid, 185 mg, 74% isolated yield.

^{1}H NMR (300 MHz, $CDCl_3$) δ 11.03 (s, 1H), 9.95 (s, 1H), 7.73 (dd, *J* = 8.7, 2.1 Hz, 1H), 7.67 (d, *J* = 2.1 Hz, 1H), 7.55 – 7.39 (m, 4H), 7.19 (d, *J* = 19.1 Hz, 1H), 7.12 – 6.97 (m, 3H), 6.16 (d, *J* = 19.1 Hz, 1H), 1.90 (dh, *J* = 13.7, 6.9 Hz, 7H), 0.99 (dd, *J* = 6.6, 3.4 Hz, 42H), 0.65 (dd, *J* = 12.1, 7.0 Hz, 14H).

^{13}C{^{1}H} NMR (101 MHz, $CDCl_3$) δ 196.68, 161.32, 147.56, 137.50, 137.24, 134.79, 131.74, 129.79, 127.76, 127.38, 127.05, 126.71, 120.76, 118.81, 118.31, 25.88, 24.05, 24.02, 22.68, 22.63.

^{29}Si{^{1}H} NMR (79 MHz, $CDCl_3$) δ -67.40, -67.81, -67.83, -79.88.

FT-IR (ATR) $\tilde{\nu}$ 2952, 2925, 2905, 2868, 1660, 1602, 1589, 1509, 1485, 1464, 1400, 1382, 1366, 1331, 1286, 1228, 1168, 1080, 989, 955, 838, 737, 693, 571, 510, 472, 430.

HR TOF MS ES$^-$ *m/z* calcd for [M-H]$^-$ $C_{45}H_{75}O_{14}Si_8$ 1063.3311, found 1063.3323.

SQ-3

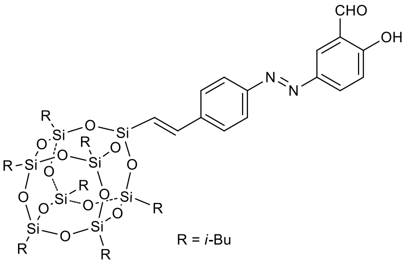

Orange solid, 206 mg, 82% isolated yield.

^{1}H NMR (300 MHz, $CDCl_3$) δ 11.33 (s, 1H), 10.04 (s, 1H), 8.29 – 8.08 (m, 2H), 7.89 (d, *J* = 8.4 Hz, 2H), 7.59 (d, *J* = 8.5 Hz, 2H), 7.24 (d, *J* = 19.1 Hz, 1H), 7.13 (d, *J* = 8.7 Hz, 1H), 6.27 (d, *J* = 19.1 Hz, 1H), 1.89 (tq, *J* = 13.2, 6.6 Hz, 7H), 0.98 (dd, *J* = 6.6, 4.4 Hz, 42H), 0.65 (dd, *J* = 13.3, 7.0 Hz, 14H).

^{13}C{^{1}H} NMR (101 MHz, $CDCl_3$) δ 196.68, 163.96, 152.43, 147.09, 146.16, 140.33, 130.86, 129.53, 127.74, 123.28, 121.16, 120.47, 118.76, 25.89, 24.06, 24.03, 22.68, 22.62.

^{29}Si{^{1}H} NMR (79 MHz, $CDCl_3$) δ -67.34, -67.79, -67.81, -80.33.

FT-IR (ATR) $\tilde{\nu}$ 2953, 2925, 2906, 2869, 1660, 1618, 1464, 1401, 1382, 1366, 1331, 1285, 1228, 1196, 1168, 1081, 989, 953, 837, 799, 738, 578, 472, 430.

HR TOF MS ES^{-} *m/z* calcd for $[M-H]^{-}$ $C_{43}H_{73}N_2O_{14}Si_8$ 1065.3216, found 1065.3223.

2.3. General procedure for the synthesis of salphenes H_2Sal-bisSQ-1–3 (Pathway A – in solution)

Aldehyde **SQ-1** or **SQ-2** or **SQ-3** (2 equiv, $9.76 \cdot 10^{-5}$ mol), and 1,2-diaminophenyl (1 equiv, $4.88 \cdot 10^{-5}$ mol) were stirred in EtOH (10 mL) under an argon atmosphere at 80 °C for 24 hrs. Formed precipitate was filtered, washed with ethanol, and dried under vacuum.

H_2Sal-bisSQ-1

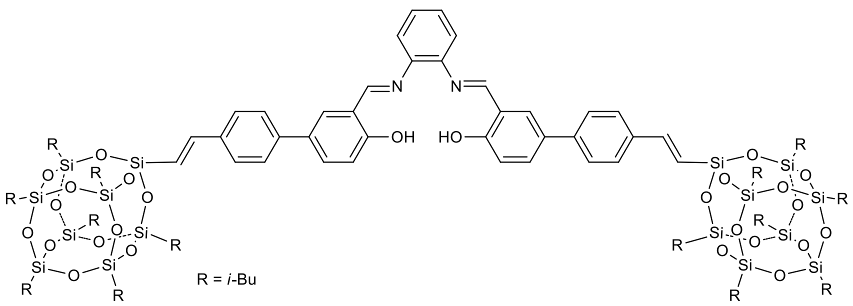

Yellow solid, 75 mg, 71% isolated yield.
^{1}H NMR (300 MHz, THF-d_8) δ 12.94 (s, 1H), 8.90 (s, 1H), 7.84 (d, J = 2.3 Hz, 1H), 7.71 (dd, J = 8.7, 2.2 Hz, 1H), 7.63 (d, J = 8.4 Hz, 2H), 7.54 (d, J = 8.4 Hz, 2H), 7.39 (d, J = 1.7 Hz, 2H), 7.25 (d, J = 19.1 Hz, 1H), 7.03 (d, J = 8.6 Hz, 1H), 6.22 (d, J = 19.2 Hz, 1H), 1.93 (tq, J = 13.4, 6.7 Hz, 7H), 1.00 (dd, J = 6.6, 2.7 Hz, 42H), 0.68 (dd, J = 10.5, 7.0 Hz, 14H).
^{13}C{^{1}H} NMR (151 MHz, THF-d_8) δ 165.64, 162.43, 149.11, 144.06, 141.77, 137.09, 132.66, 132.24, 131.65, 128.68, 128.31, 127.40, 120.88, 120.82, 118.66, 118.43, 26.34, 26.32, 25.06, 25.04, 23.50, 23.48, 23.41.
^{29}Si{^{1}H} NMR (119 MHz, THF-d_8) δ -67.40, -67.78, -67.79, -79.40.
FT-IR (ATR) $\tilde{\nu}$ 2952, 2925, 2905, 2868, 1611, 1575, 1484, 1464, 1401, 1382, 1365, 1331, 1297, 1281, 1228, 1205, 1168, 1083, 990, 953, 837, 795, 740, 563, 473, 429.
MALDI TOF^{+} MS *m/z* calcd for $[M+H]^{+}$ $C_{92}H_{153}N_2O_{26}Si_{16}$ 2150.7020, found 2150.7.

H_2Sal-bisSQ-2

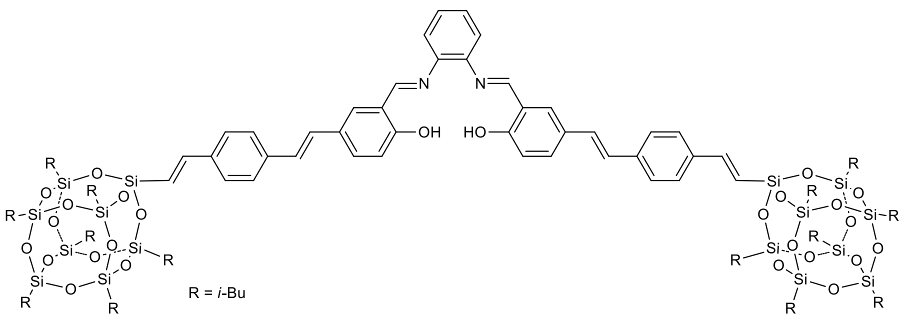

Yellow solid, 70 mg, 65% isolated yield.
^{1}H NMR (300 MHz, THF-d_8) δ 12.96 (s, 1H), 8.83 (s, 1H), 7.73 – 7.63 (m, 2H), 7.52 (d, J = 8.4 Hz, 2H), 7.45 (d, J = 8.4 Hz, 2H), 7.38 (s, 2H), 7.25 – 7.05 (m, 3H), 6.96 (d, J = 8.6 Hz, 1H), 6.18 (d, J = 19.1 Hz, 1H), 1.99 – 1.85 (m, 7H), 1.00 (dd, J = 6.6, 2.5 Hz, 42H), 0.67 (dd, J = 9.8, 7.0 Hz, 14H).
^{13}C{^{1}H} NMR (151 MHz, THF-d_8) δ 165.50, 162.58, 149.16, 144.00, 139.66, 137.45, 132.19, 131.93, 129.75, 129.25, 128.69, 128.12, 127.45, 127.03, 120.86, 120.66, 118.58, 118.18, 26.33, 26.31, 25.06, 25.04, 23.50, 23.48, 23.41.
^{29}Si{^{1}H} NMR (119 MHz, THF-d_8) δ -67.42, -67.79, -67.80, -79.36.
FT-IR (ATR) $\tilde{\nu}$ 2953, 2925, 2906, 2869, 1617, 1573, 1509, 1488, 1464, 1400, 1382, 1365, 1332, 1287, 1228, 1088, 988, 958, 837, 738, 565, 475, 431.
MALDI TOF^{+} MS *m/z* calcd for $[M+H]^{+}$ $C_{96}H_{157}N_2O_{26}Si_{16}$ 2201.7288, found 2201.7.

H_2Sal-bisSQ-3

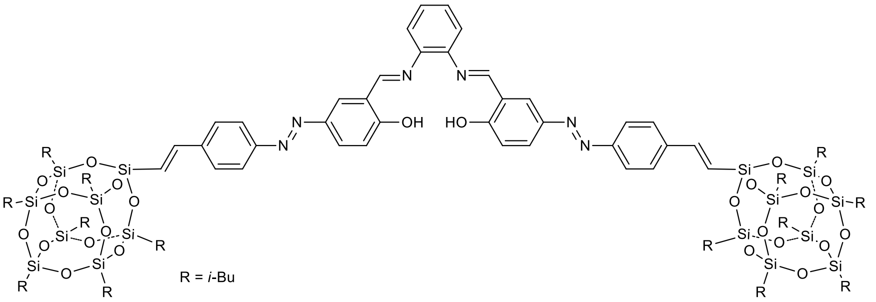

Orange solid, 52 mg, 48% isolated yield.

^{1}H NMR (300 MHz, THF-d_8) δ 13.59 (s, 1H), 9.01 (s, 1H), 8.21 (d, J = 2.2 Hz, 1H), 8.04 (dd, J = 8.9, 2.3 Hz, 1H), 7.89 (d, J = 8.4 Hz, 2H), 7.64 (d, J = 8.5 Hz, 2H), 7.52 – 7.40 (m, 2H), 7.29 (d, J = 19.1 Hz, 1H), 7.11 (d, J = 8.9 Hz, 1H), 6.33 (d, J = 19.2 Hz, 1H), 1.93 (dh, J = 13.0, 6.6 Hz, 7H), 1.00 (dd, J = 6.5, 3.4 Hz, 42H), 0.68 (dd, J = 11.3, 7.0 Hz, 14H).

^{13}C{^{1}H} NMR (151 MHz, THF-d_8) δ 165.71, 165.48, 153.86, 148.58, 146.72, 143.59, 140.60, 129.89, 129.09, 128.59, 128.08, 123.93, 120.89, 120.77, 120.51, 119.11, 26.34, 26.32, 25.06, 25.04, 23.49, 23.47, 23.39.

^{29}Si{^{1}H} NMR (119 MHz, THF-d_8) δ -67.34, -67.76, -67.77, -79.82.

FT-IR (ATR) $\tilde{\nu}$ 2953, 2926, 2906, 2893, 2869, 1615, 1485, 1463, 1401, 1382, 1365, 1332, 1285, 1227, 1168, 1080, 989, 952, 836, 739, 576, 472, 430.

MALDI TOF$^+$ MS *m/z* calcd for $[M+H]^+$ $C_{92}H_{153}N_6O_{26}Si_{16}$ 2206.7176, found 2206.7.

2.4. General procedure for the synthesis of complexes Zn@Sal-bisSQ-1–3 (Pathway B – in solution)

Aldehyde **SQ-1** or **SQ-2** or **SQ-3** (2 equiv, $9.76{\cdot}10^{-5}$ mol), 1,2-diaminophenyl (1 equiv, $4.88{\cdot}10^{-5}$ mol), and $Zn(OAc)_2$ (1 equiv, $4.88{\cdot}10^{-5}$ mol) were stirred in EtOH (10 mL) under an argon atmosphere at 80 °C for 24 hrs. Formed precipitate was filtered, washed with EtOH, and dried under vacuum.

2.5. General procedure for the synthesis of complexes Zn@Sal-bisSQ-1–3 (Pathway C – in ball mill)

Aldehyde **SQ-1** or **SQ-2** or **SQ-3** (2 equiv, $9.76{\cdot}10^{-5}$ mol), 1,2-diaminophenyl (1 equiv, $4.88{\cdot}10^{-5}$ mol), and $Zn(OAc)_2$ (1 equiv, $4.88{\cdot}10^{-5}$ mol) and EtOH (20 µL) were placed in ball mill chamber. Substrates were mixing for 2 hrs, with frequency 25 Hz. Formed precipitate was washed with EtOH (1 mL), and dried under vacuum.

Zn@Sal-bisSQ-1

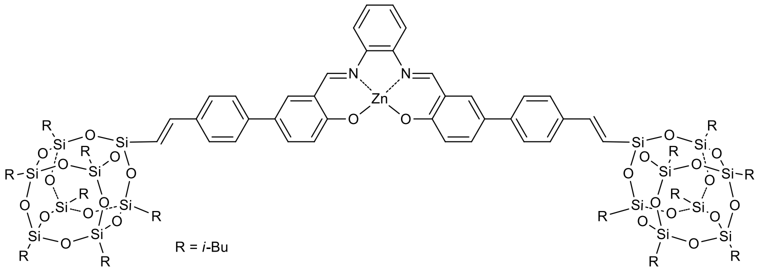

Orange solid; 93 mg, 86% isolated yield (Pathway B); 97 mg, 90% isolated yield (Pathway C).
^{1}H NMR (300 MHz, THF-d_8) δ 9.07 (s, 1H), 7.93 – 7.81 (m, 1H), 7.68 (d, J = 2.4 Hz, 2H), 7.60 (d, J = 8.4 Hz, 2H), 7.49 (d, J = 8.3 Hz, 2H), 7.42 – 7.32 (m, 1H), 7.24 (d, J = 19.1 Hz, 1H), 6.92 (d, J = 8.0 Hz, 1H), 6.17 (d, J = 19.1 Hz, 1H), 1.92 (tt, J = 13.1, 6.6 Hz, 7H), 1.01 (dd, J = 6.6, 3.5 Hz, 42H), 0.68 (dd, J = 10.9, 7.0 Hz, 14H).
^{13}C{^{1}H} NMR (151 MHz, pyridine-d_5) δ 174.65, 164.07, 149.57, 142.46, 141.08, 134.92, 134.22, 128.39, 128.36, 128.34, 126.72, 125.95, 125.66, 120.72, 118.13, 117.33, 26.33, 26.31, 24.79, 23.48, 23.39.
^{29}Si{^{1}H} NMR (119 MHz, THF-d_8) δ -67.43, -67.79, -67.80, -79.22.
FT-IR (ATR) $\tilde{\nu}$ 2952, 2925, 2906, 2869, 1616, 1582, 1527, 1509, 1464, 1401, 1382, 1366, 1331, 1228, 1168, 1084, 835, 798, 740, 544, 474, 430.
MALDI TOF^{+} MS *m/z* calcd for $[M+H]^{+}$ $C_{92}H_{151}N_2O_{26}Si_{16}Zn$ 2214.6157, found 2214.4.

Zn@Sal-bisSQ-2

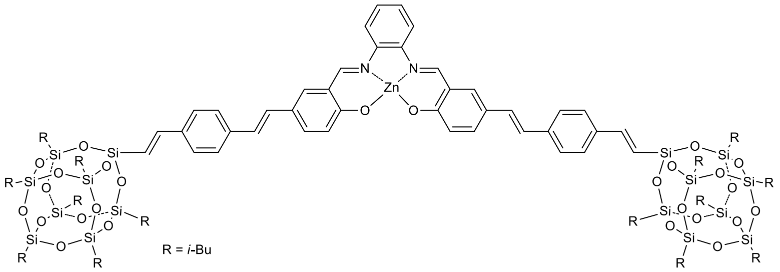

Orange solid; 89 mg, 81% isolated yield (Pathway B); 99 mg, 90% isolated yield (Pathway C).
^{1}H NMR (300 MHz, THF-d_8) δ 8.97 (s, 1H), 7.90 – 7.76 (m, 1H), 7.64 – 7.31 (m, 7H), 7.20 (d, J = 19.1 Hz, 1H), 7.12 (d, J = 16.2 Hz, 1H), 6.89 (d, J = 16.1 Hz, 2H), 6.15 (d, J = 19.1 Hz, 1H), 2.01 – 1.83 (m, 7H), 1.00 (dd, J = 6.6, 2.6 Hz, 42H), 0.67 (dd, J = 9.9, 7.0 Hz, 14H).
^{13}C{^{1}H} NMR (151 MHz, THF-d_8) δ 175.09, 163.05, 149.32, 140.94, 140.47, 136.69, 136.01, 133.01, 130.31, 128.08, 128.09, 126.98, 125.53, 123.94, 123.72, 120.23, 117.47, 116.92, 26.34, 26.32, 25.06, 25.04, 23.50, 23.48, 23.41.
^{29}Si{^{1}H} NMR (119 MHz, THF-d_8) δ -67.43, -67.79, -67.81, -79.22.

FT-IR (ATR) $\tilde{\nu}$ 2952, 2925, 2906, 2893, 2869, 1612, 1582, 1524, 1464, 1382, 1366, 1331, 1227, 1088, 954, 836, 740, 577, 509, 475, 430.
MALDI TOF$^+$ MS *m/z* calcd for $[M+H]^+$ $C_{96}H_{155}N_2O_{26}Si_{16}Zn$ 2266.6470, found 2266.7.

Zn@Sal-bisSQ-3

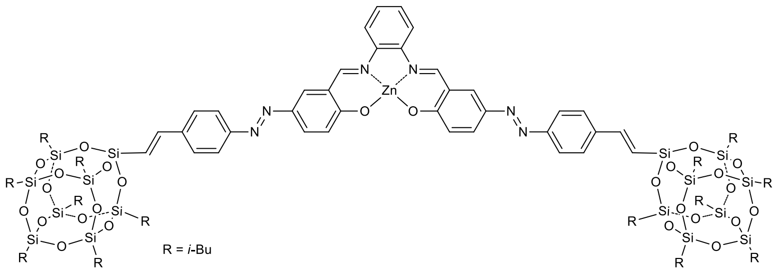

Orange solid; 84 mg, 76% isolated yield (Pathway B); 103 mg, 94% isolated yield (Pathway C).
^{1}H NMR (300 MHz, THF-d_8) δ 9.14 (s, 1H), 8.08 (d, J = 2.6 Hz, 1H), 8.01 – 7.88 (m, 2H), 7.82 (d, J = 8.4 Hz, 2H), 7.60 (d, J = 8.6 Hz, 2H), 7.47 – 7.37 (m, 1H), 7.28 (d, J = 19.1 Hz, 1H), 6.91 (d, J = 9.3 Hz, 1H), 6.28 (d, J = 19.1 Hz, 1H), 1.93 (dq, J = 13.3, 6.7 Hz, 7H), 1.01 (dd, J = 6.6, 4.0 Hz, 42H), 0.68 (dd, J = 11.7, 7.0 Hz, 14H).
^{13}C{^{1}H} NMR (151 MHz, THF-d_8) δ 177.83, 163.38, 154.50, 148.89, 143.03, 140.80, 139.30, 137.49, 128.64, 128.47, 127.29, 125.91, 123.37, 120.06, 119.63, 117.31, 26.33, 26.31, 25.07, 25.04, 23.48, 23.40.
^{29}Si{^{1}H} NMR (119 MHz, THF-d_8) δ -67.37, -67.77, -67.79, -79.61.
FT-IR (ATR) $\tilde{\nu}$ 2952, 2925, 2906, 2869, 1609, 1583, 1525, 1465, 1379, 1331, 1228, 1194, 1093, 836, 802, 741, 478, 431.
MALDI TOF$^+$ MS *m/z* calcd for $[M+H]^+$ $C_{92}H_{151}N_2O_{26}Si_{16}Zn$ 2270.6280, found 2270.6.

3. Copies of NMR spectra

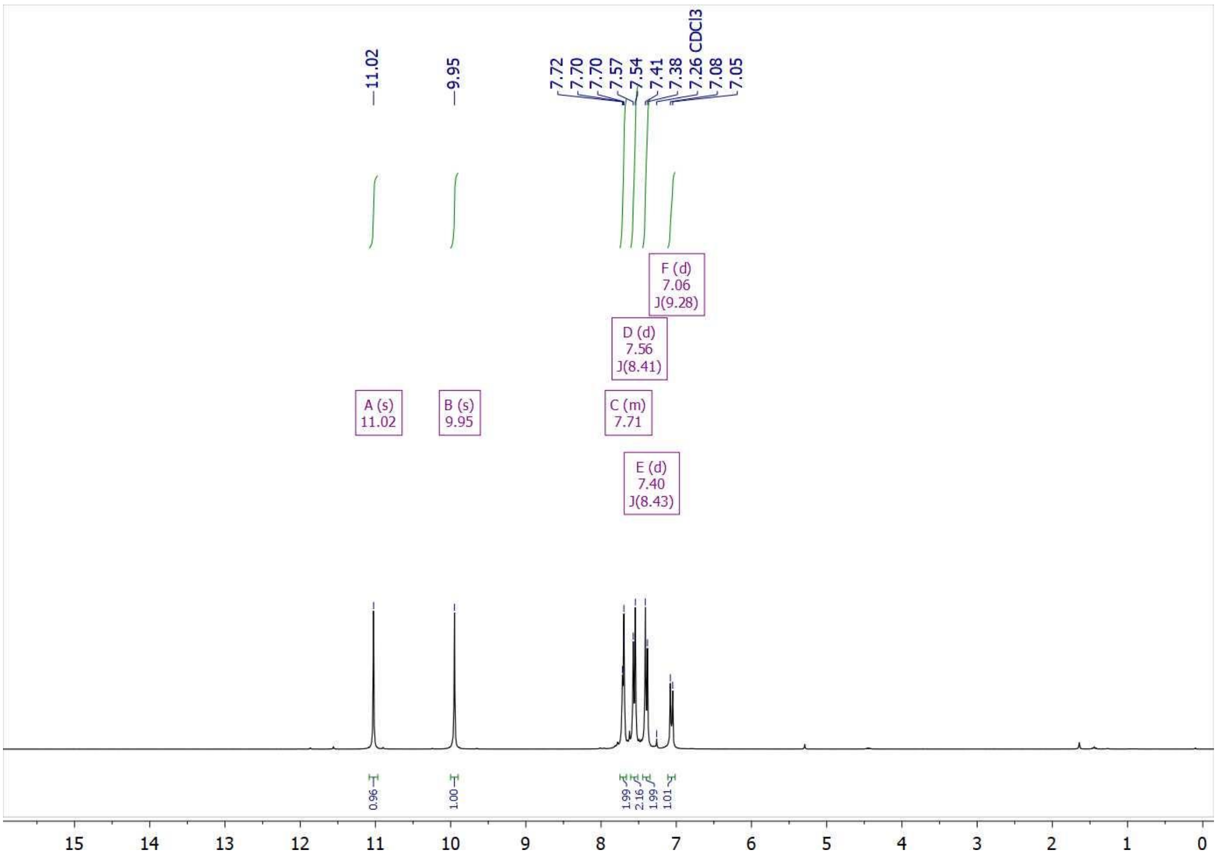

Figure SI-1. ^{1}H NMR spectrum of compound **ald-1**.

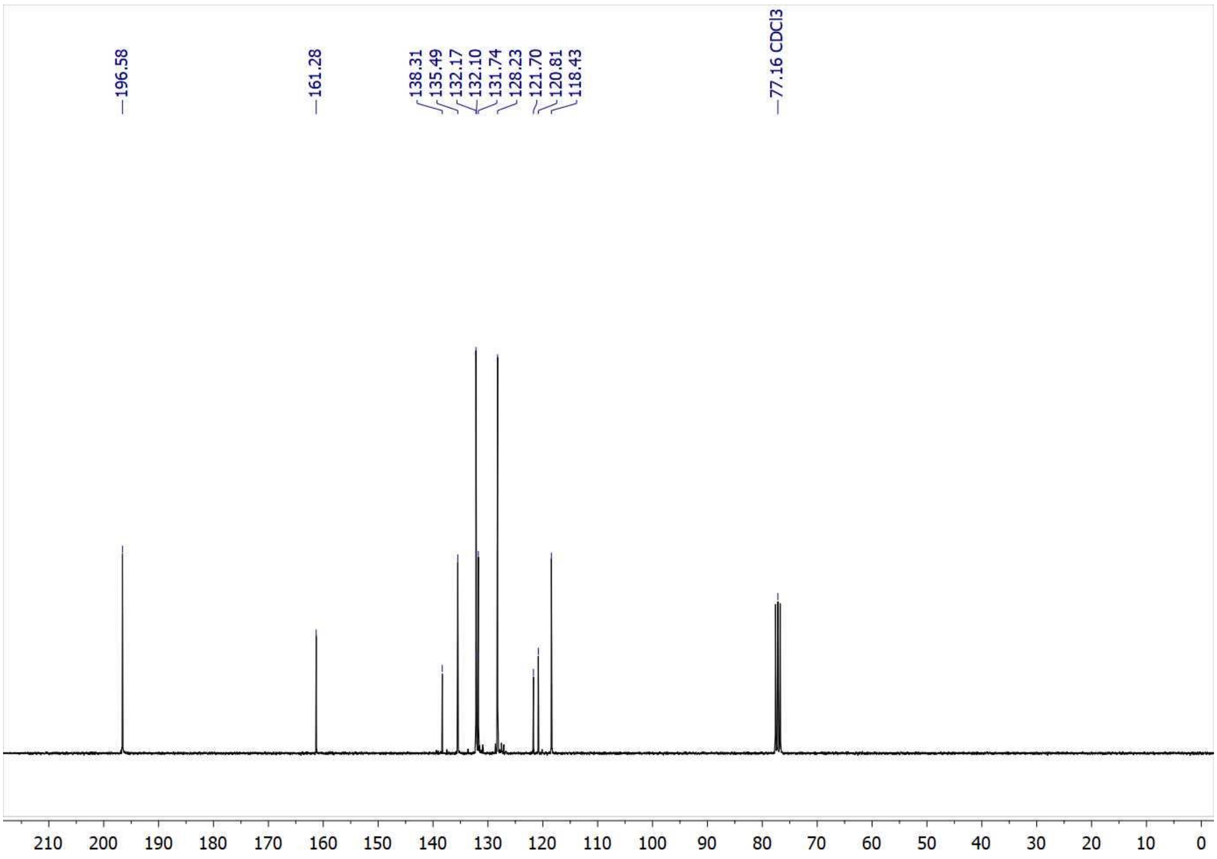

Figure SI-2. $^{13}C\{^{1}H\}$ NMR spectrum of compound **ald-1**.

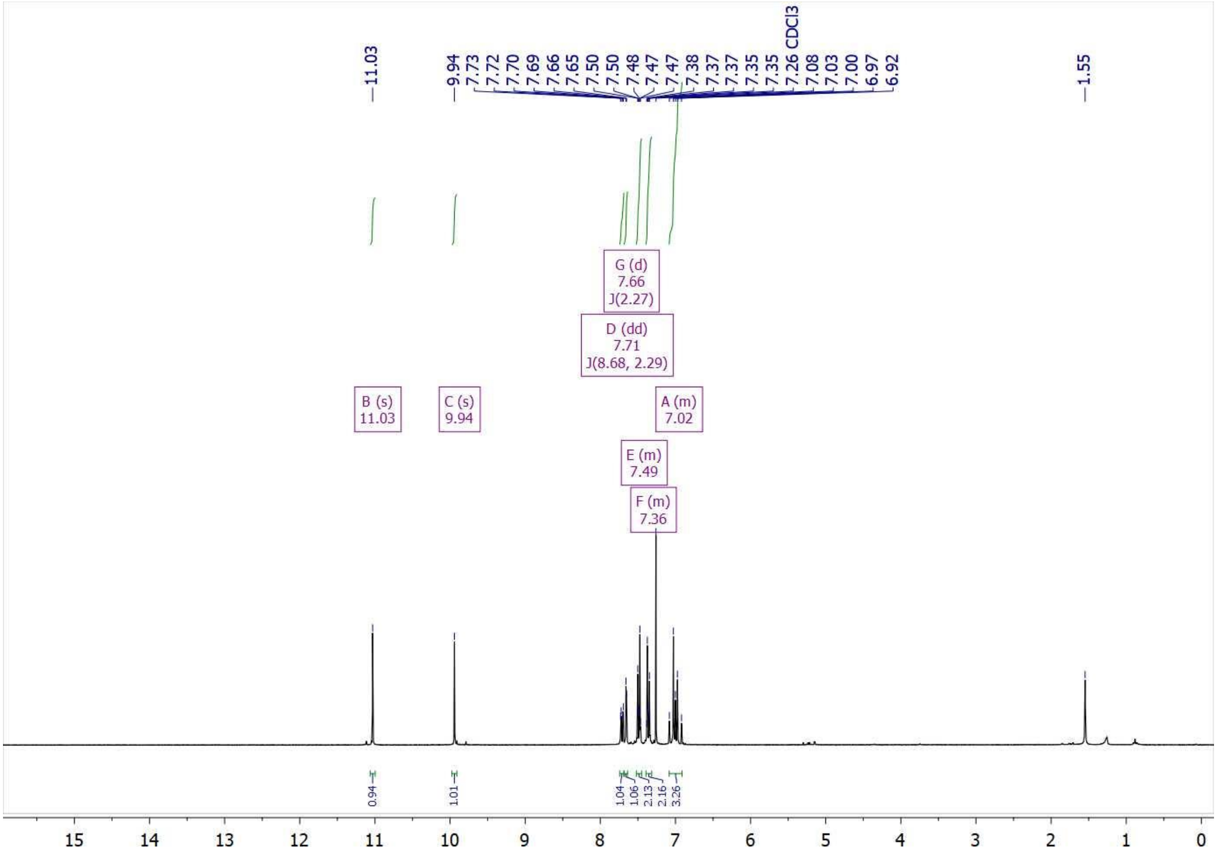

Figure SI-3. ^{1}H NMR spectrum of compound **ald-2**.

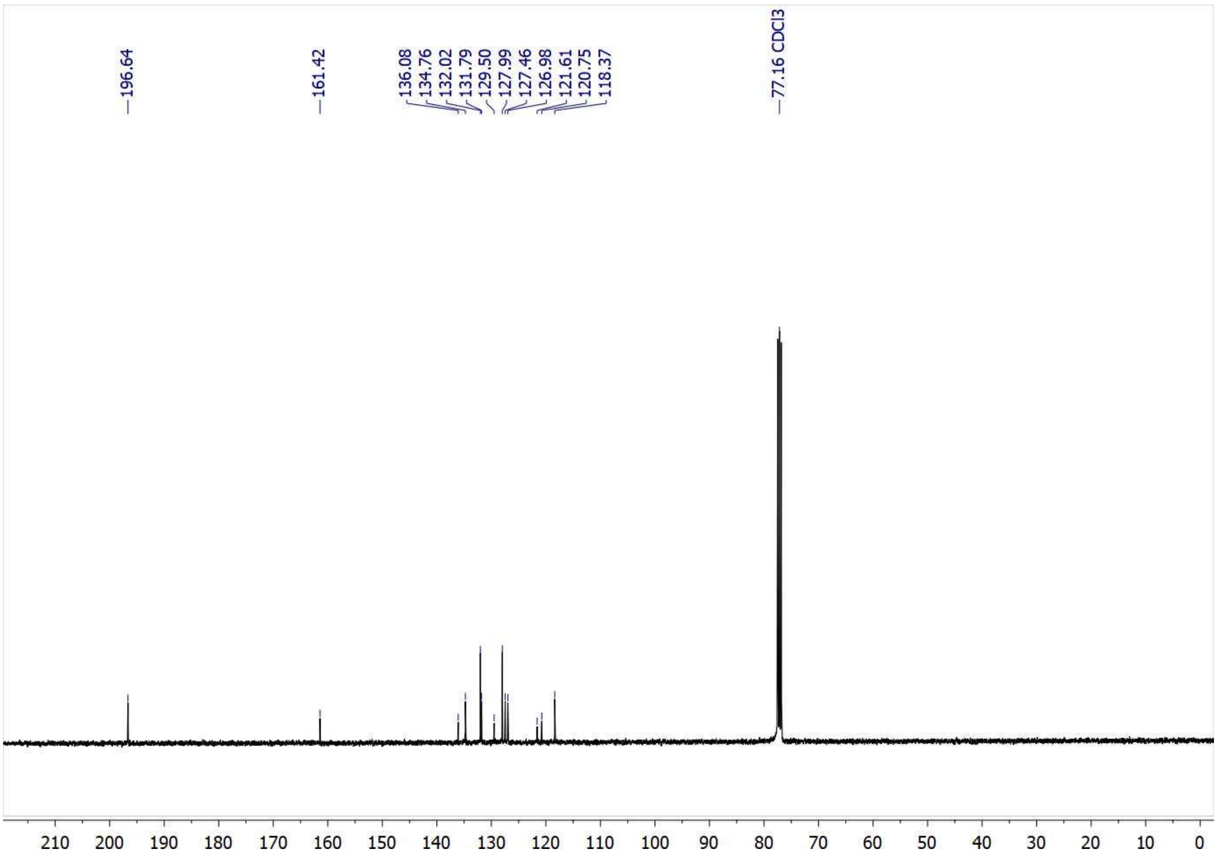

Figure SI-4. $^{13}C\{^{1}H\}$ NMR spectrum of compound **ald-2**.

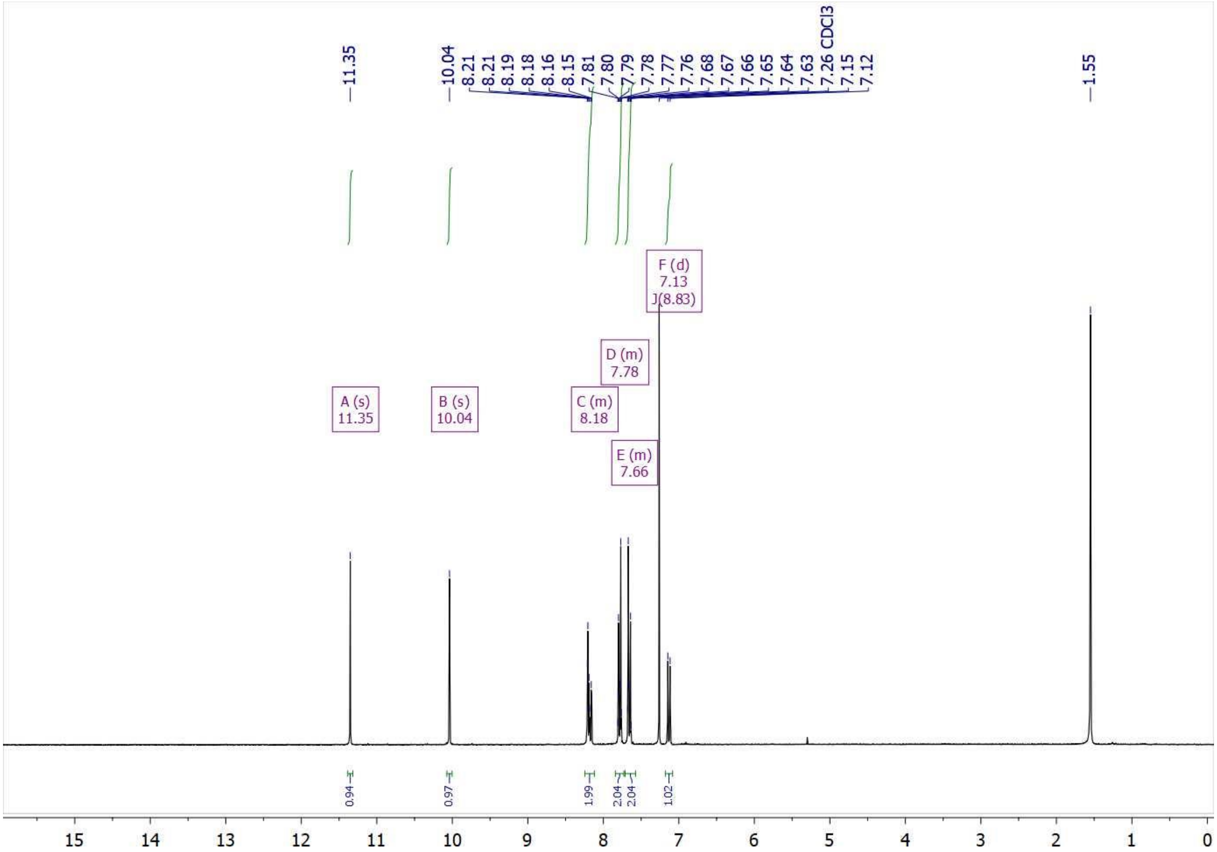

Figure SI-5. ^{1}H NMR spectrum of compound **ald-3**.

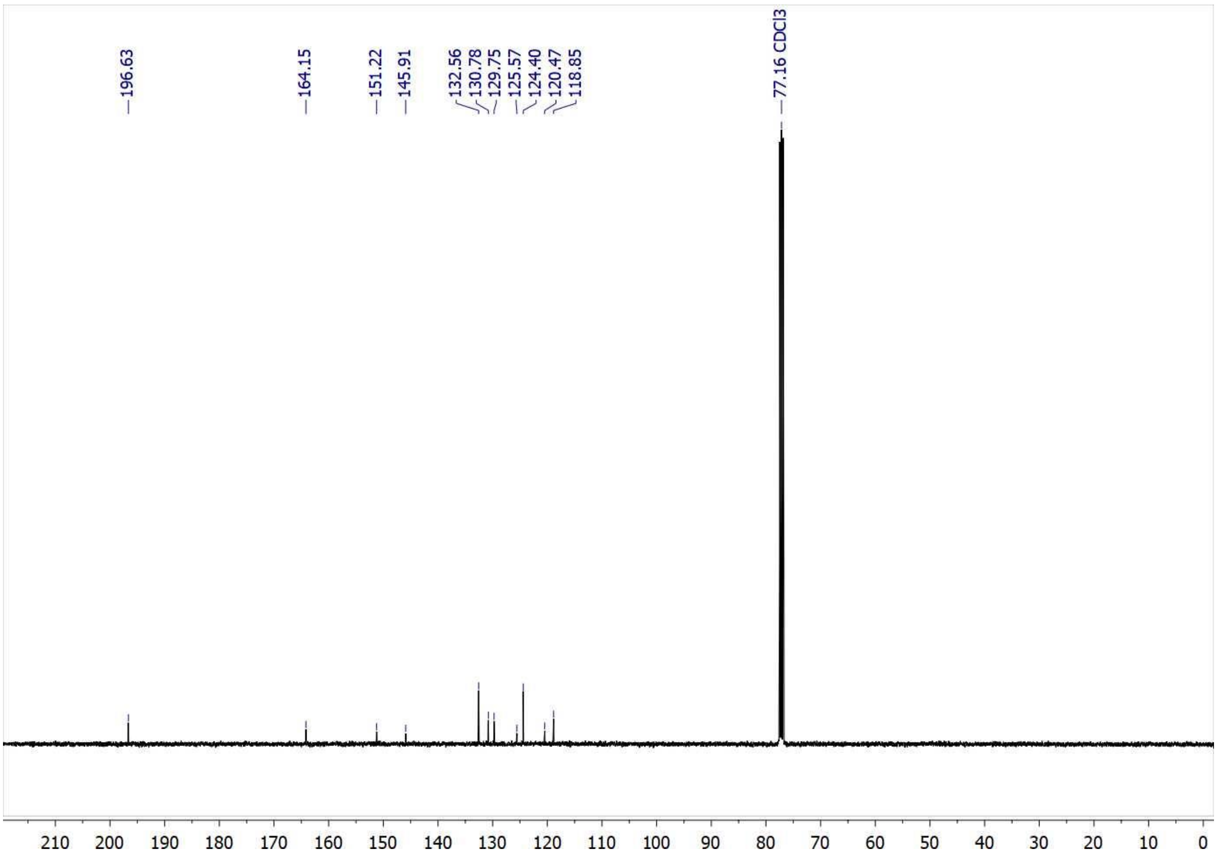

Figure SI-6. $^{13}C\{^{1}H\}$ NMR spectrum of compound **ald-3**.

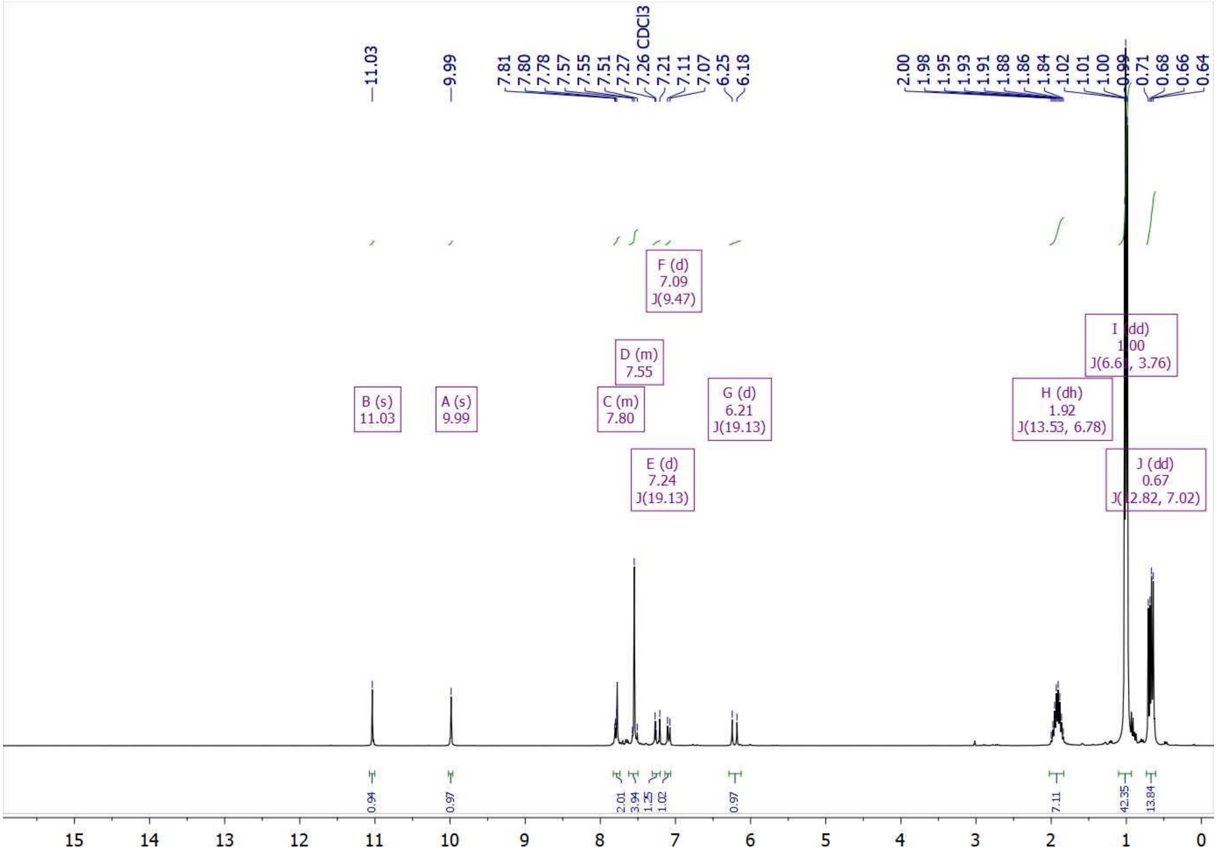

Figure SI-7. ^{1}H NMR spectrum of compound **SQ-1**.

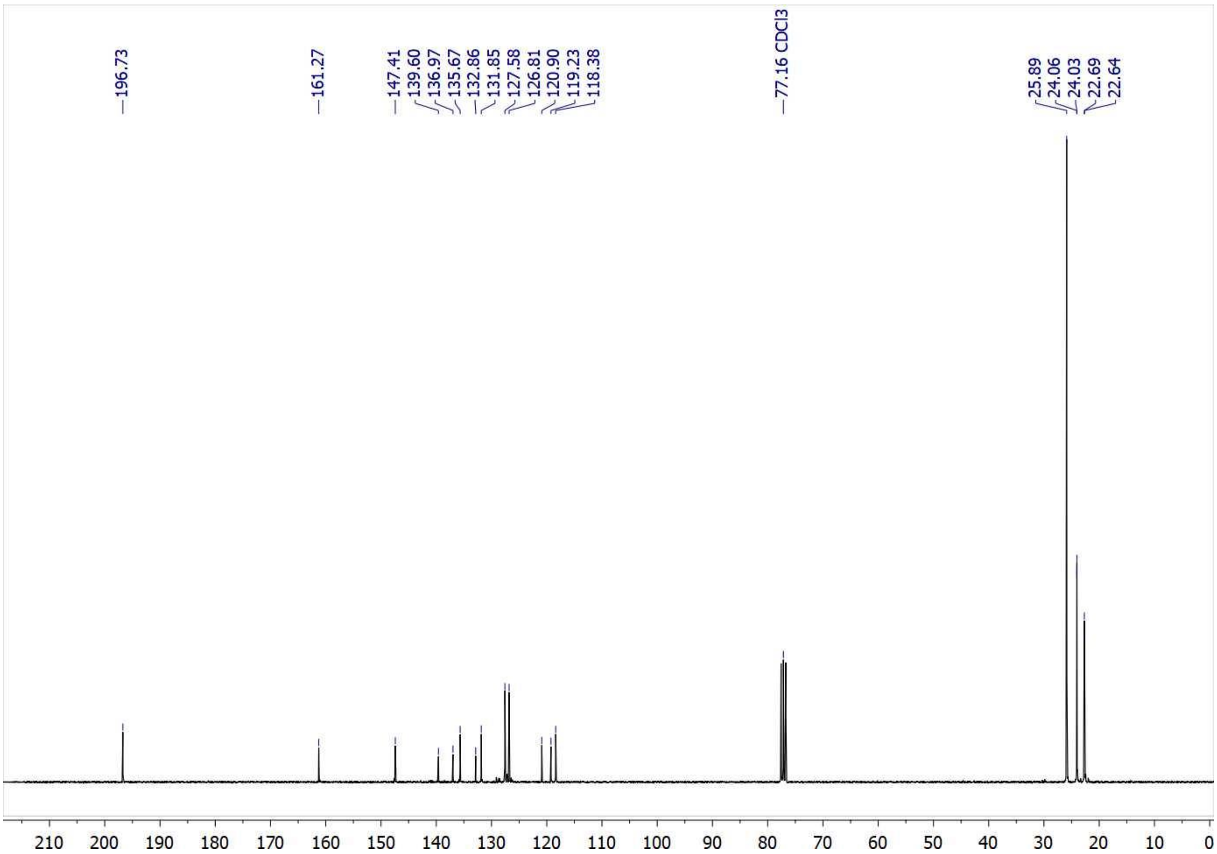

Figure SI-8. ^{13}C{^{1}H} NMR spectrum of compound **SQ-1**.

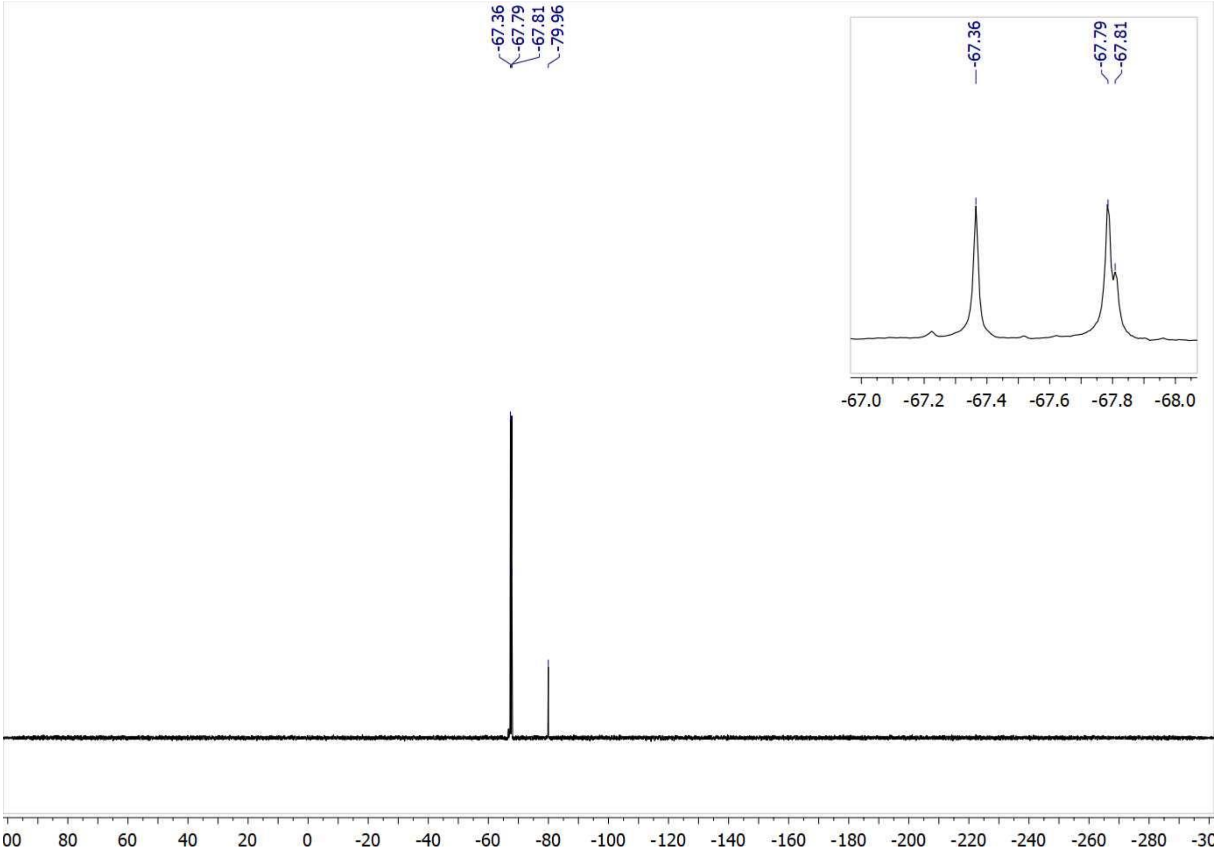

Figure SI-9. $^{29}Si\{^1H\}$ NMR spectrum of compound **SQ-1**.

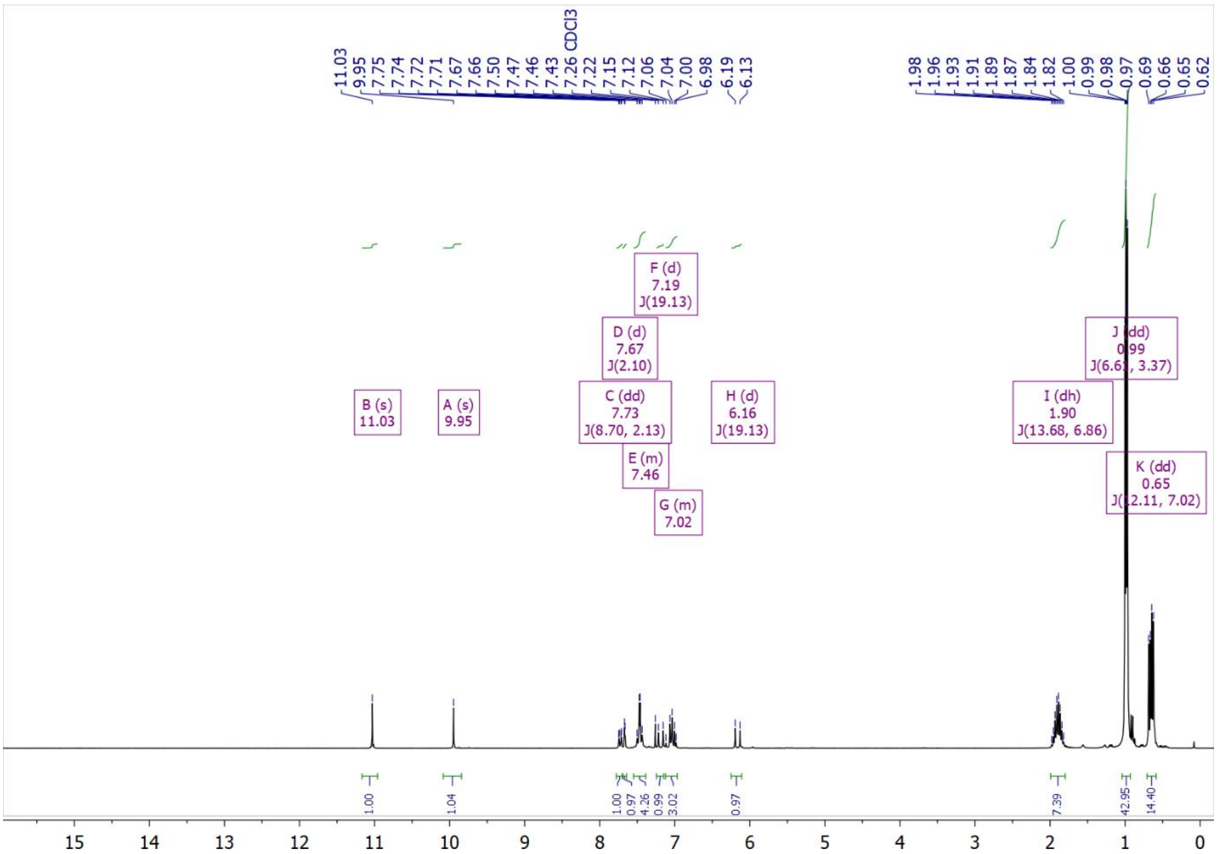

Figure SI-10. ^1H NMR spectrum of compound **SQ-2**.

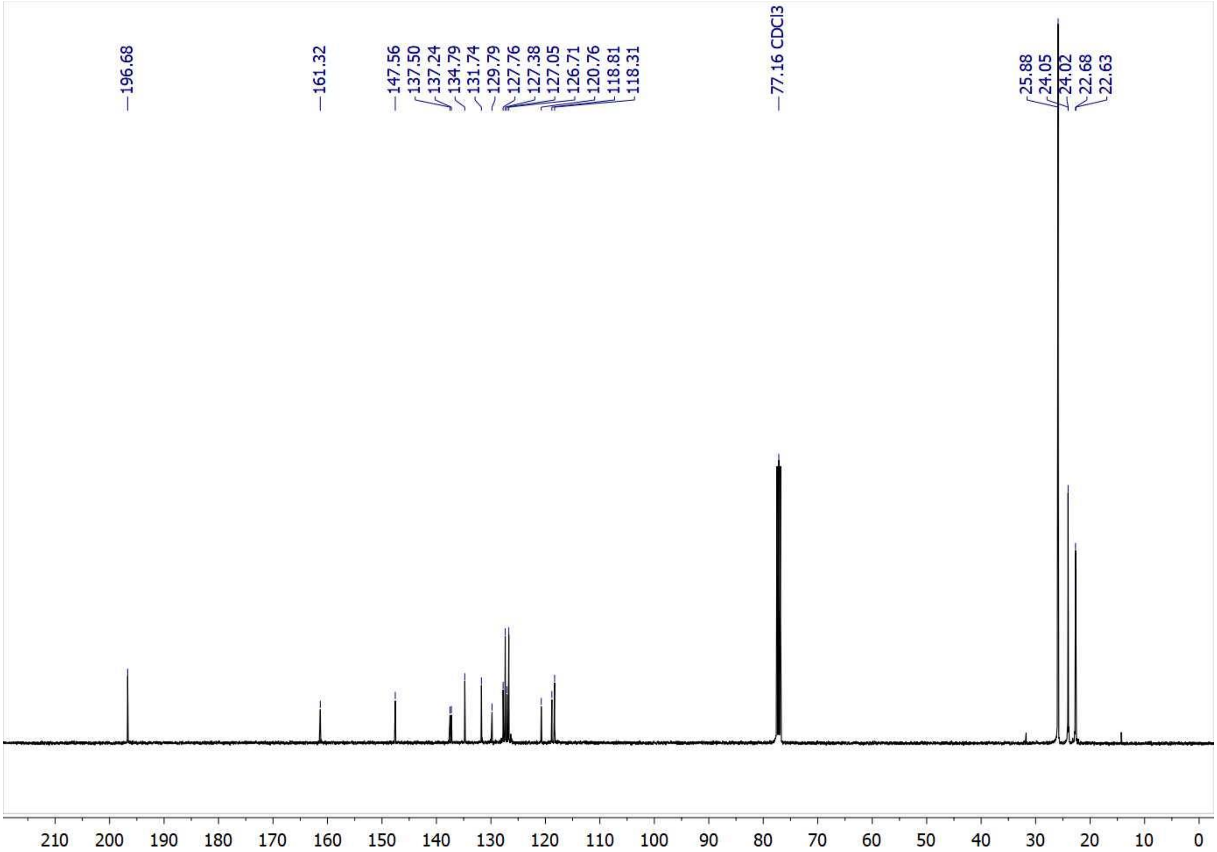

Figure SI-11. $^{13}C\{^{1}H\}$ NMR spectrum of compound **SQ-2**.

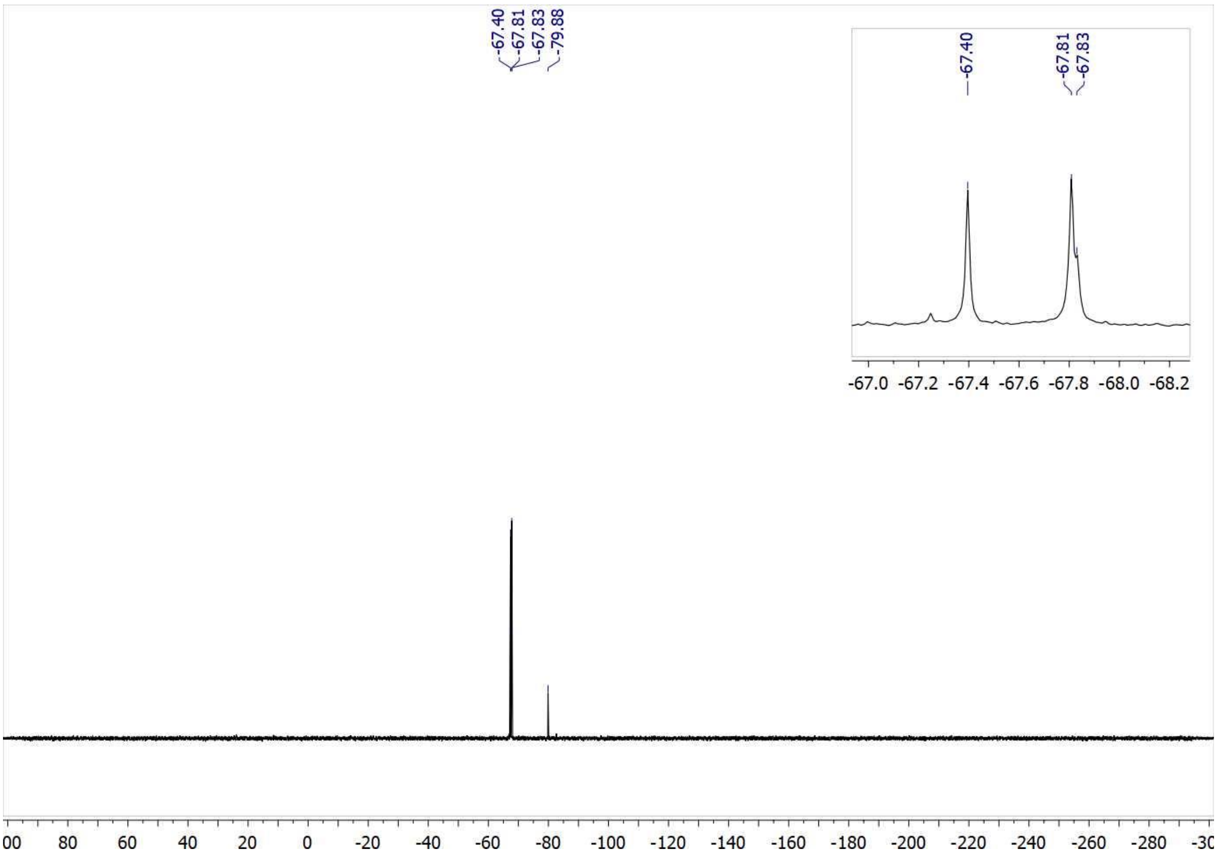

Figure SI-12. $^{29}Si\{^{1}H\}$ NMR spectrum of compound **SQ-2.**

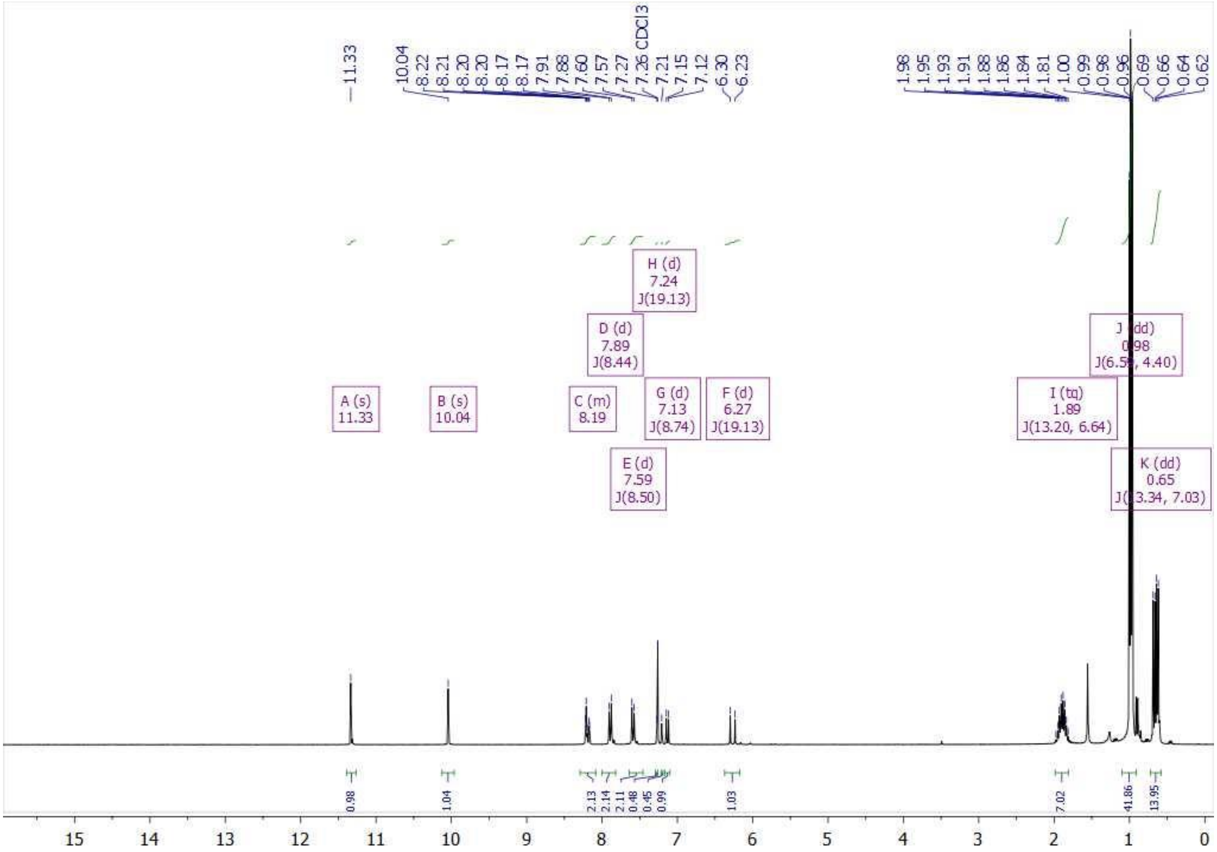

Figure SI-13. ^{1}H NMR spectrum of compound **SQ-3**.

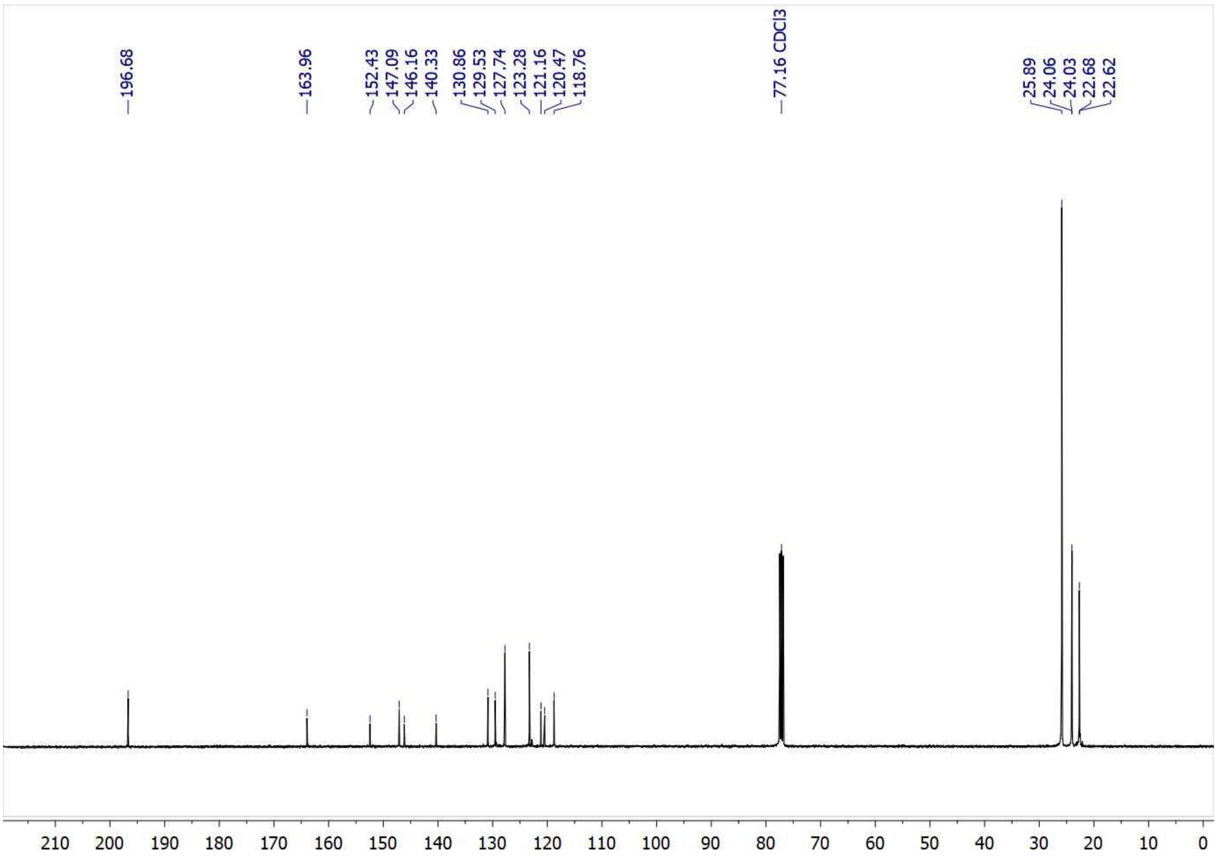

Figure SI-14. ^{13}C{^{1}H} NMR spectrum of compound **SQ-3**.

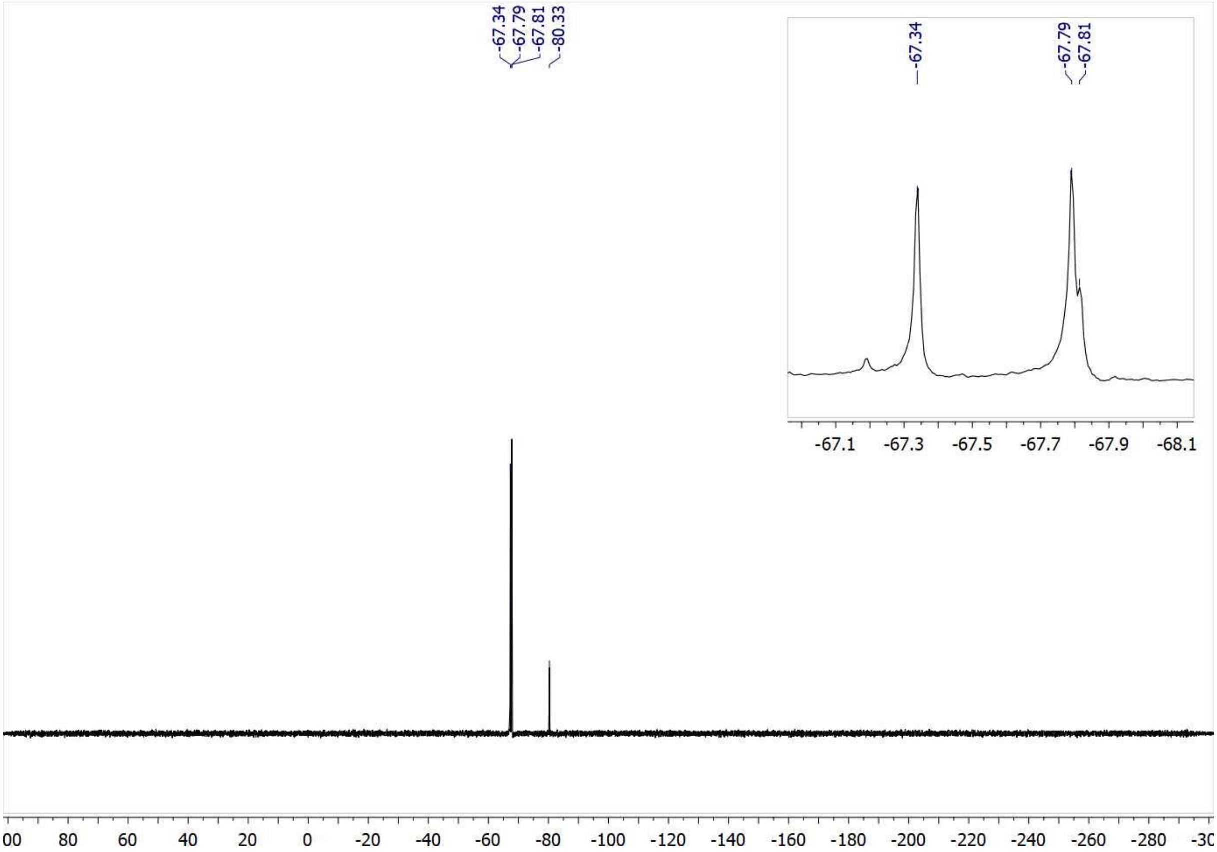

Figure SI-15. $^{29}Si\{^{1}H\}$ NMR spectrum of compound **SQ-3**.

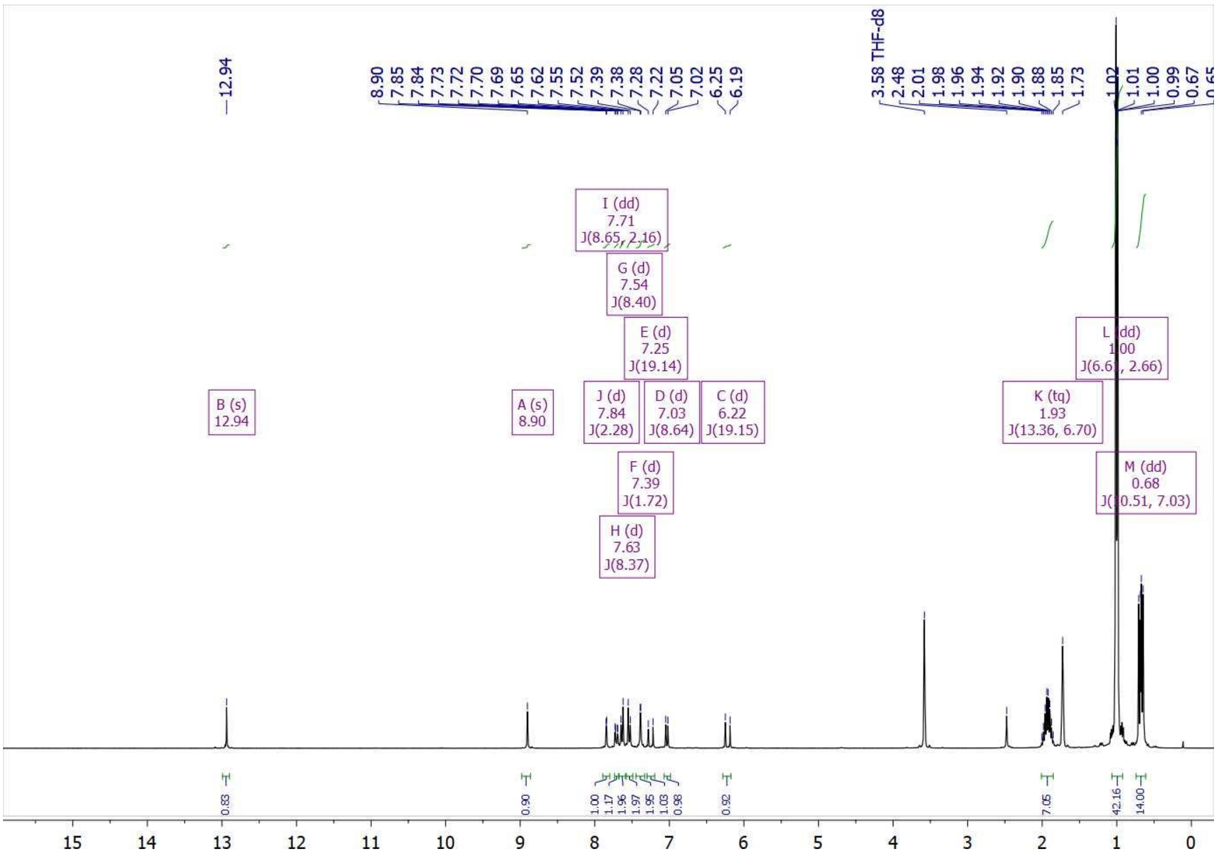

Figure SI-16. ^{1}H NMR spectrum of compound **H_2Sal-bisSQ-1**.

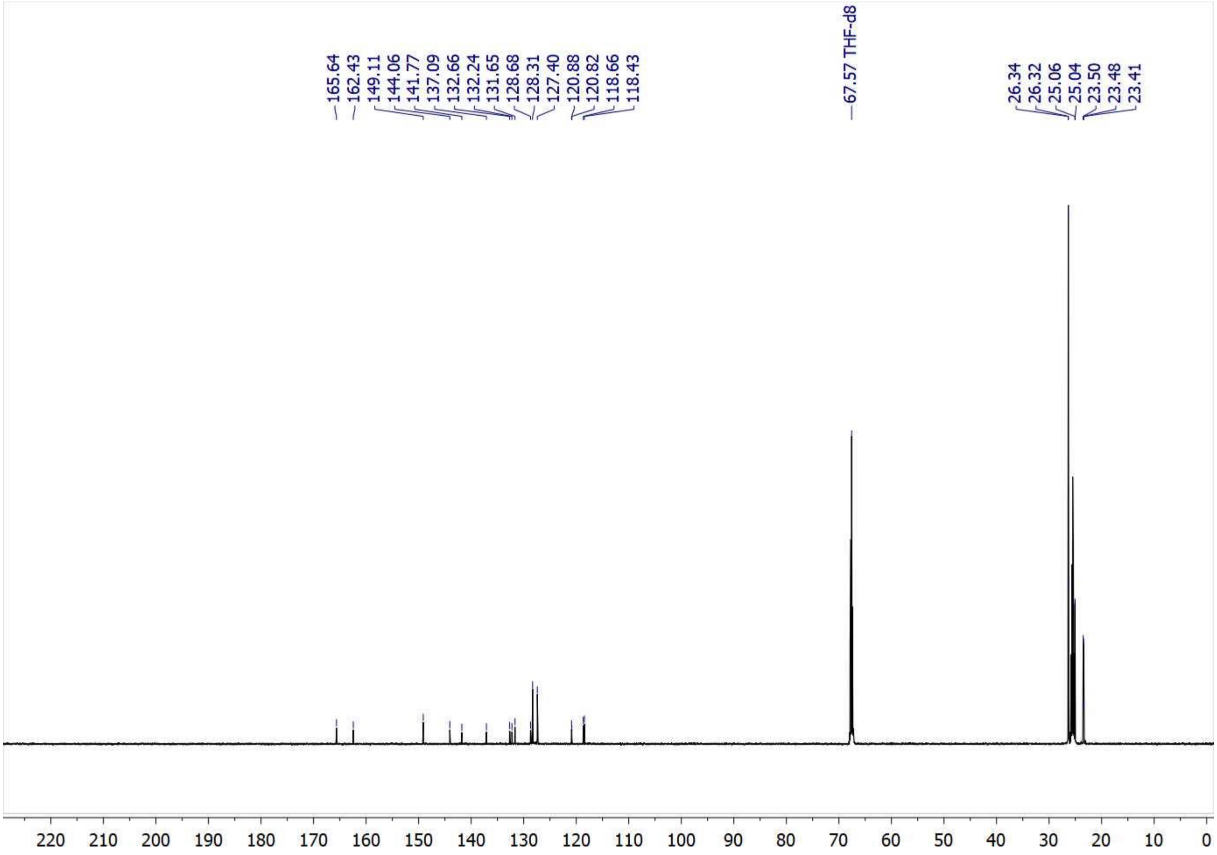

Figure SI-17. $^{13}C\{^{1}H\}$ NMR spectrum of compound **H_2Sal-bisSQ-1**.

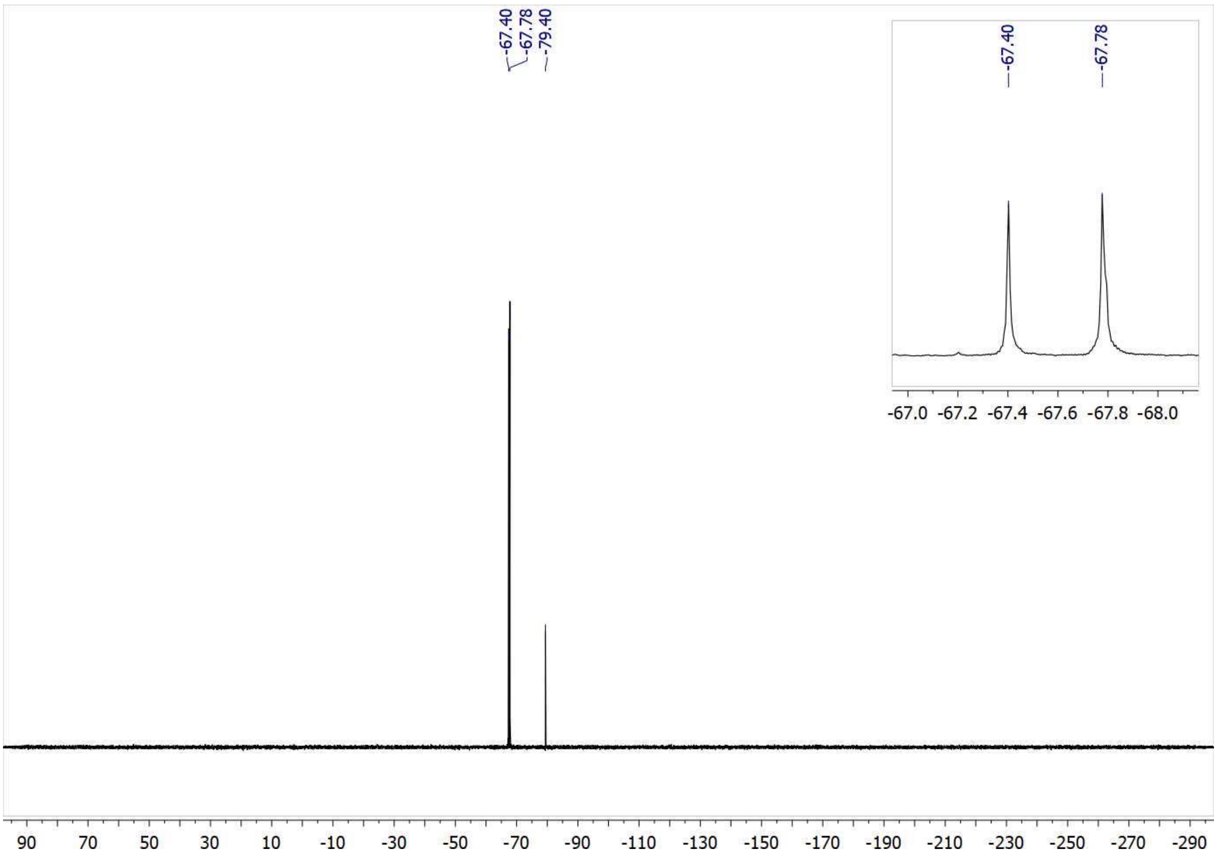

Figure SI-18. $^{29}Si\{^{1}H\}$ NMR spectrum of compound **H_2Sal-bisSQ-1.**

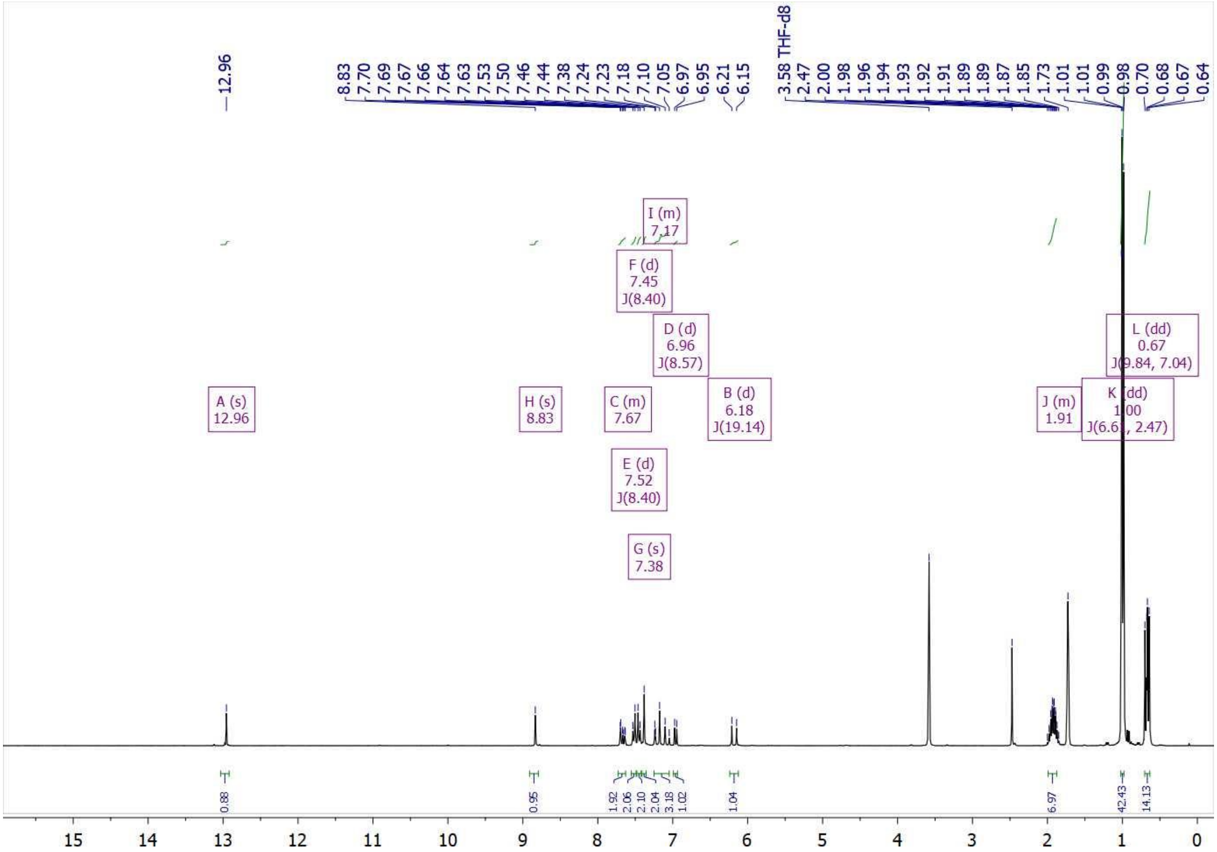

Figure SI-19. ^{1}H NMR spectrum of compound **H$_2$Sal-bisSQ-2**.

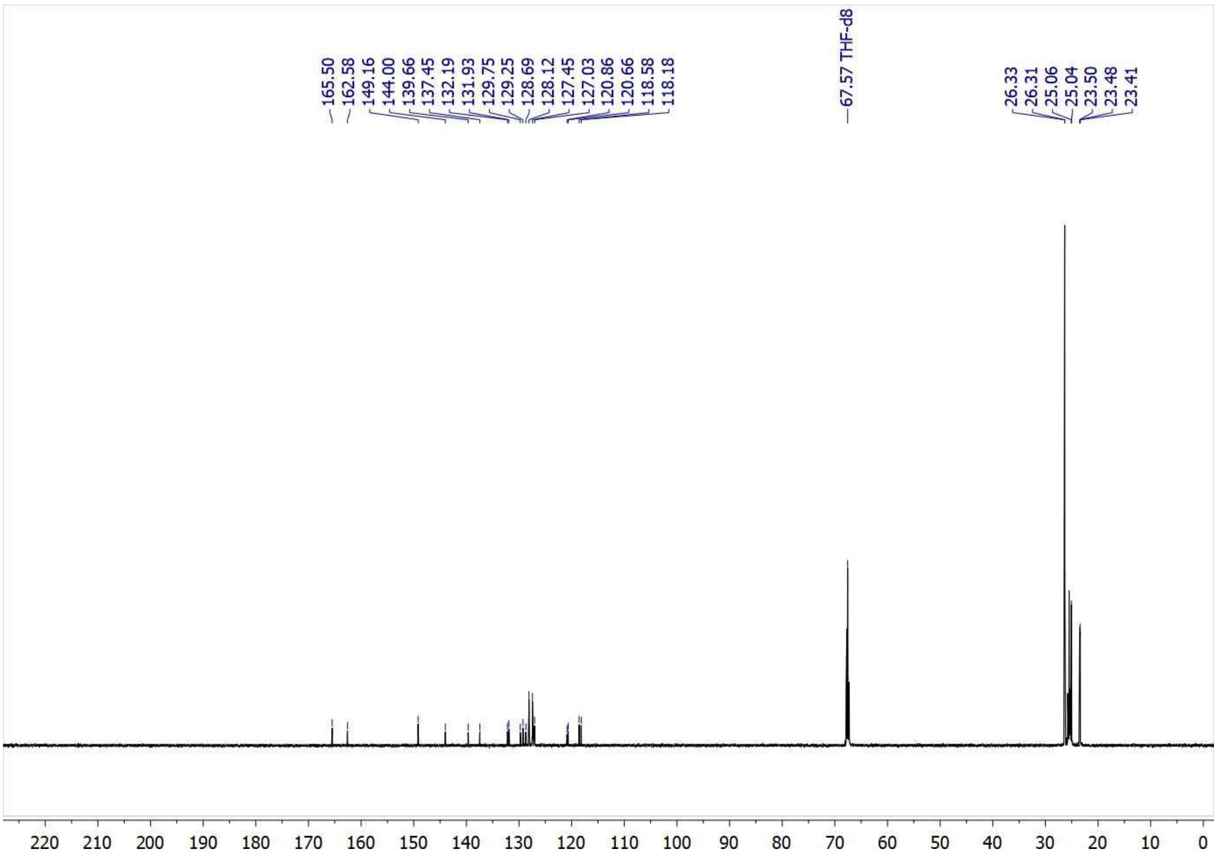

Figure SI-20. $^{13}C\{^{1}H\}$ NMR spectrum of compound **H$_2$Sal-bisSQ-2**.

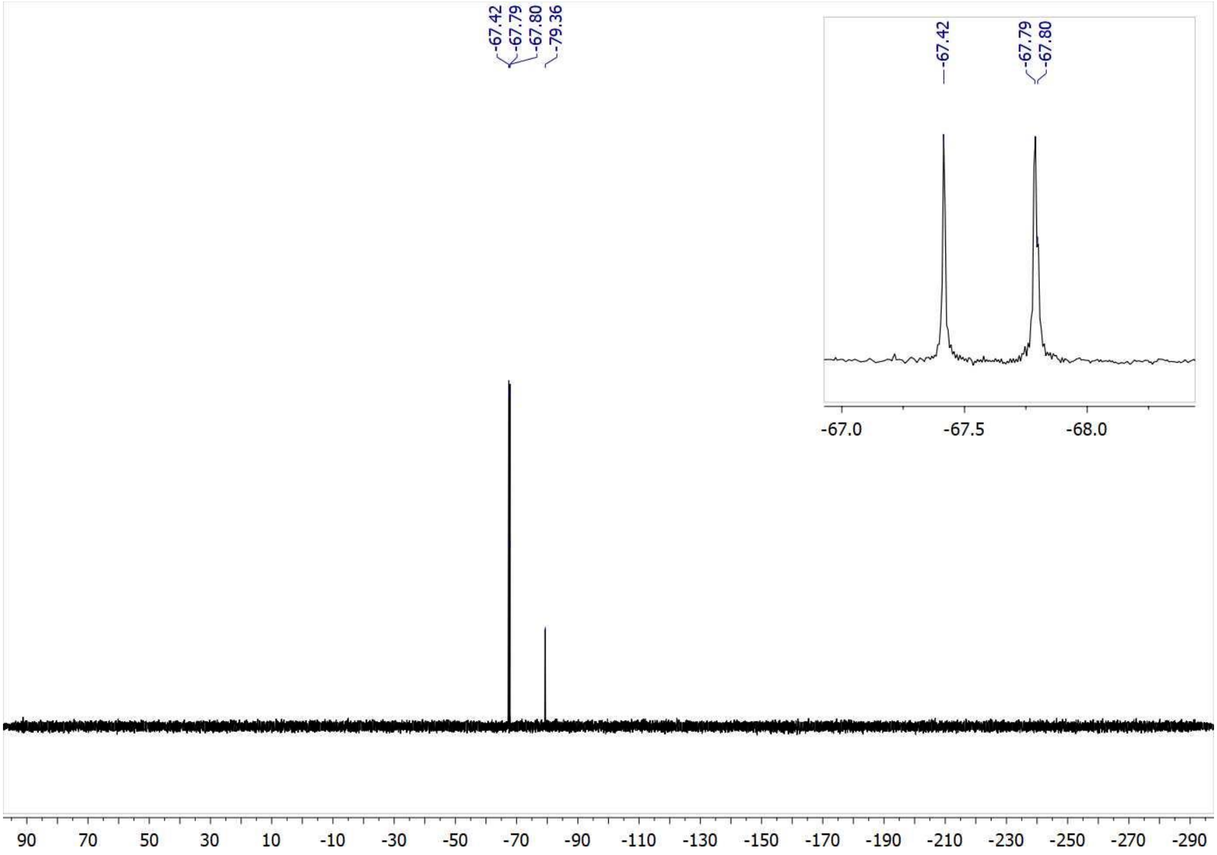

Figure SI-21. $^{29}Si\{^{1}H\}$ NMR spectrum of compound **H_2Sal-bisSQ-2**.

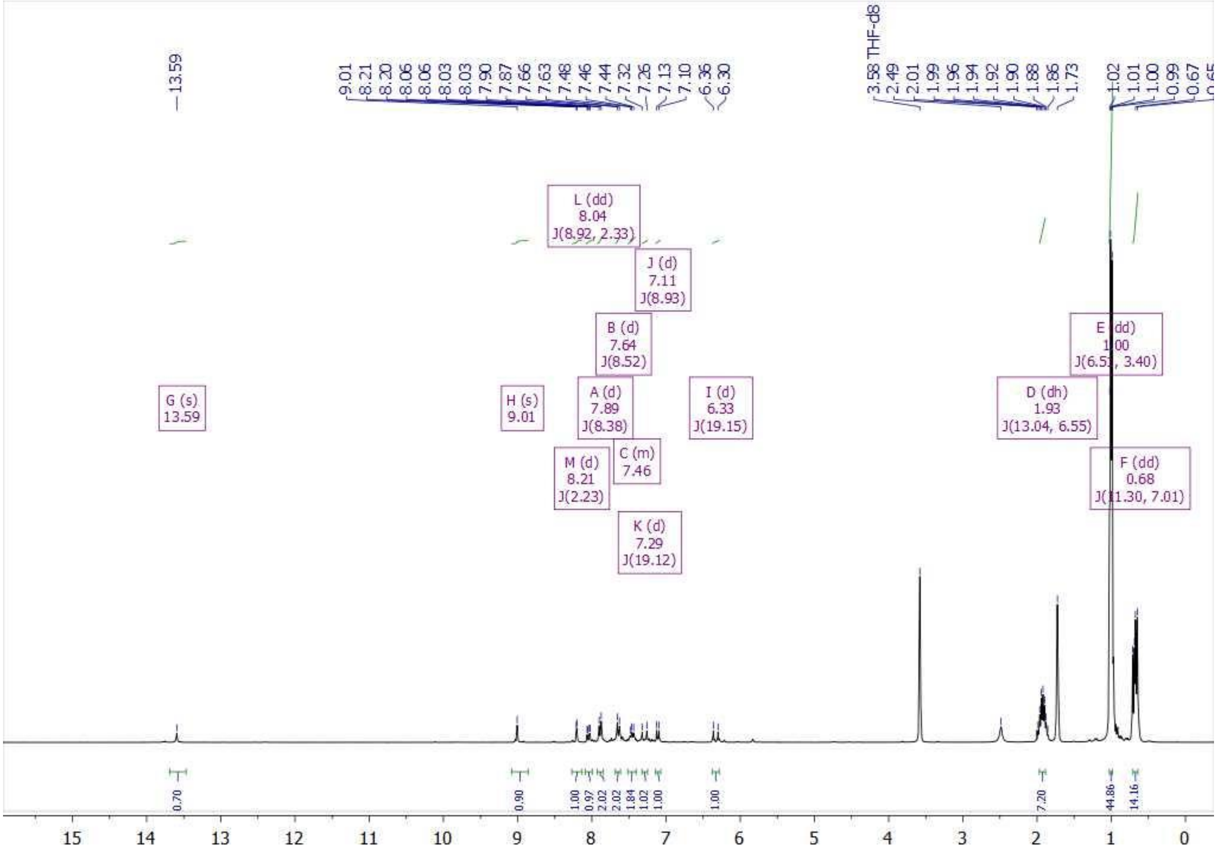

Figure SI-22. ^{1}H NMR spectrum of compound **H_2Sal-bisSQ-3**.

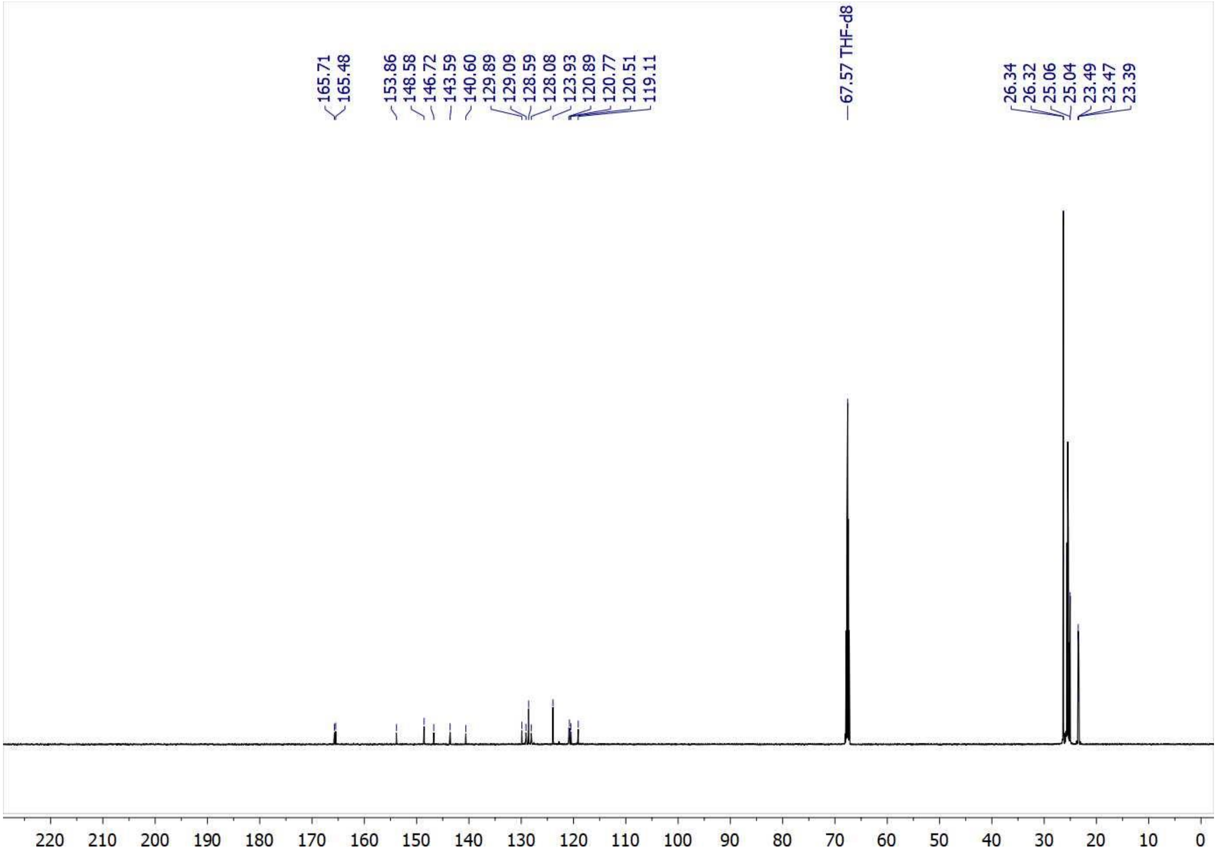

Figure SI-23. $^{13}C\{^{1}H\}$ NMR spectrum of compound **H_2Sal-bisSQ-3**.

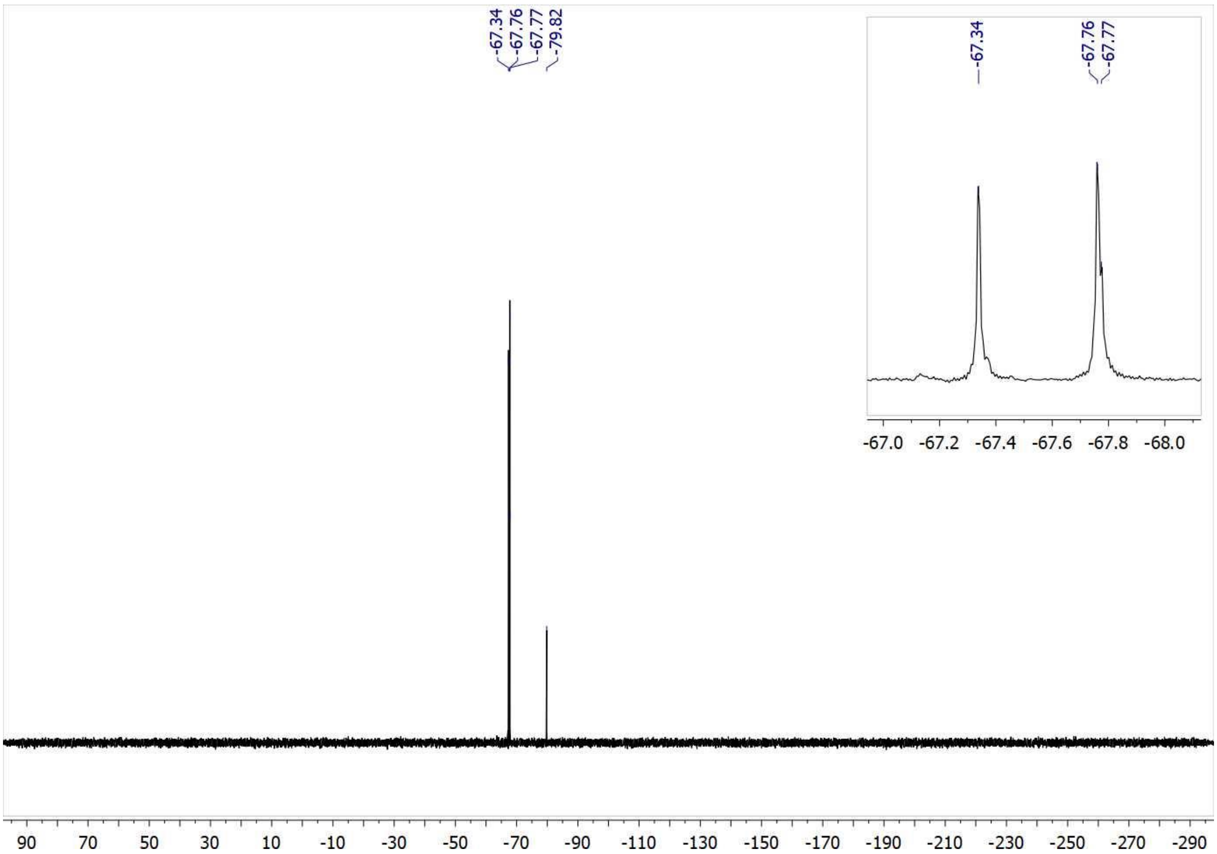

Figure SI-24. $^{29}Si\{^{1}H\}$ NMR spectrum of compound **H_2Sal-bisSQ-3**.

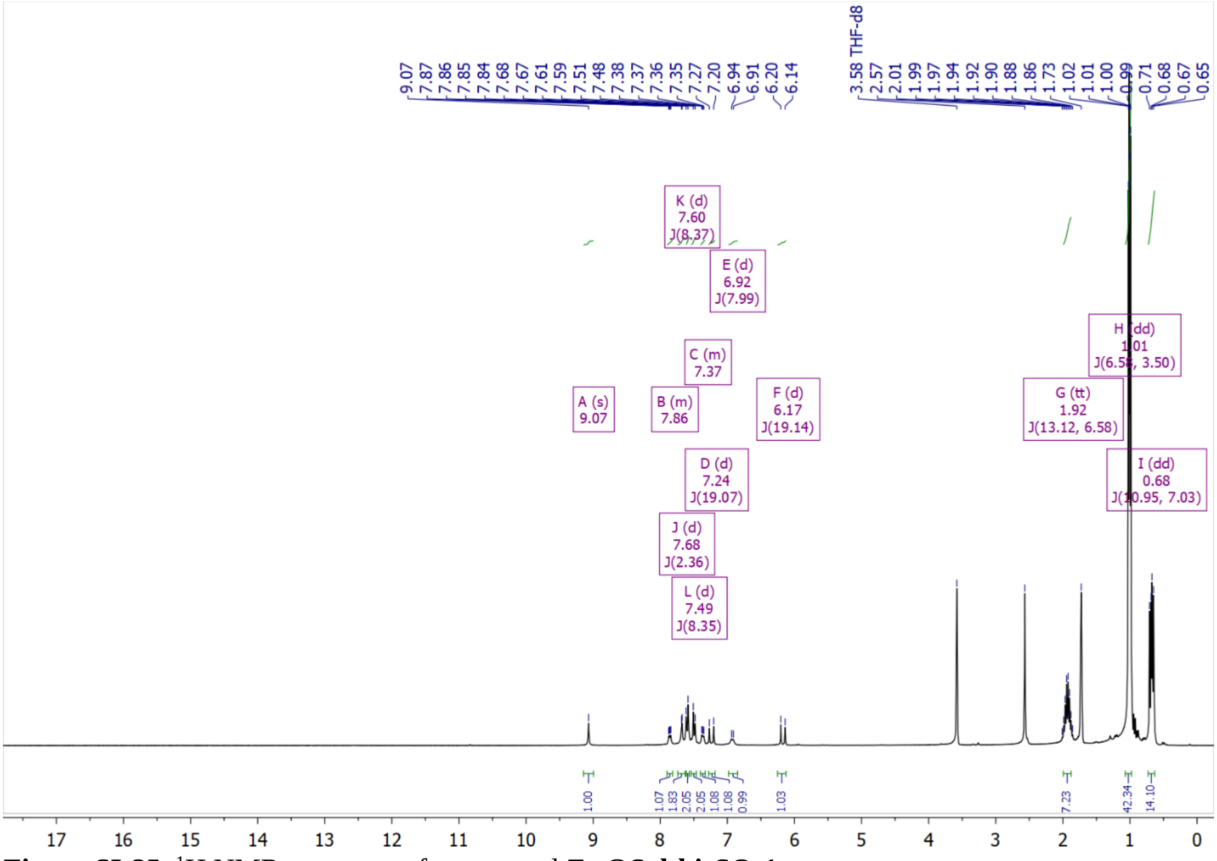

Figure SI-25. ^{1}H NMR spectrum of compound **Zn@Sal-bisSQ-1**.

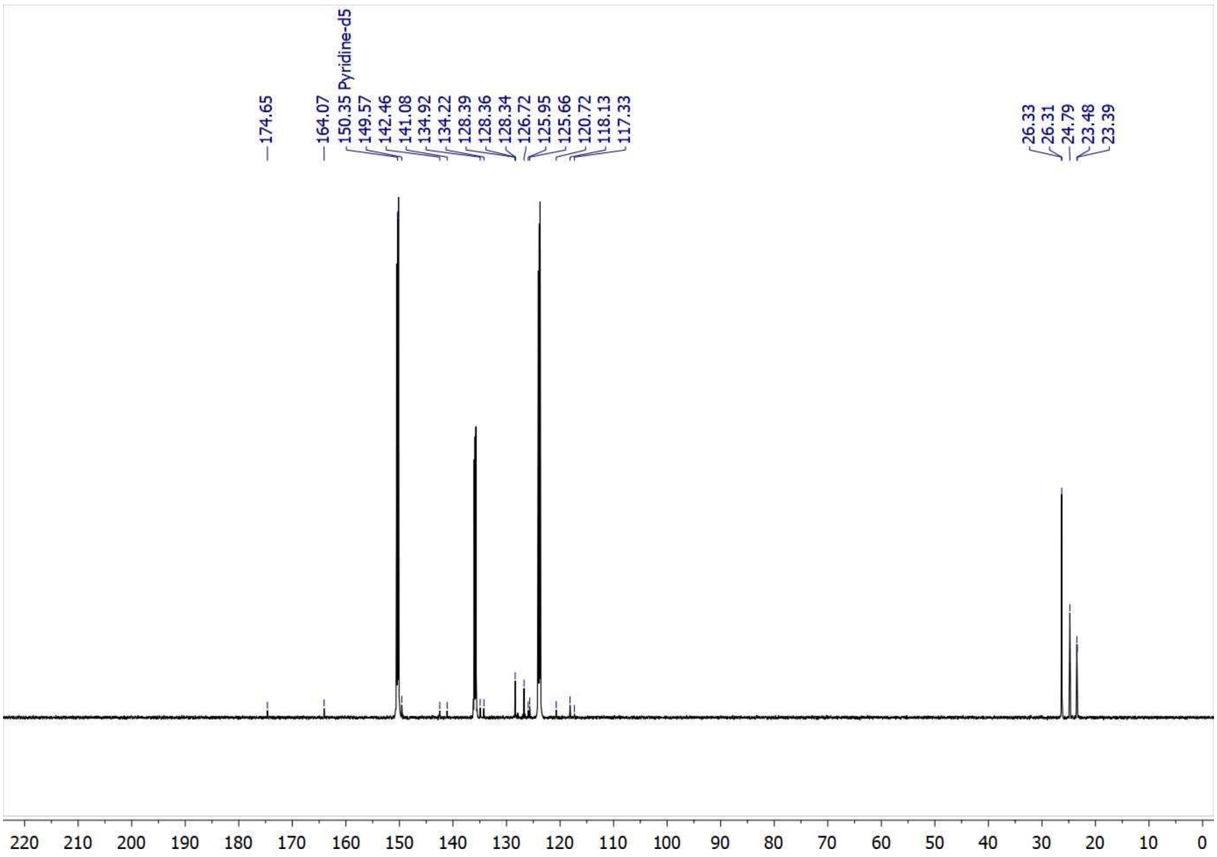

Figure SI-26. $^{13}C\{^{1}H\}$ NMR spectrum of compound **Zn@Sal-bisSQ-1**.

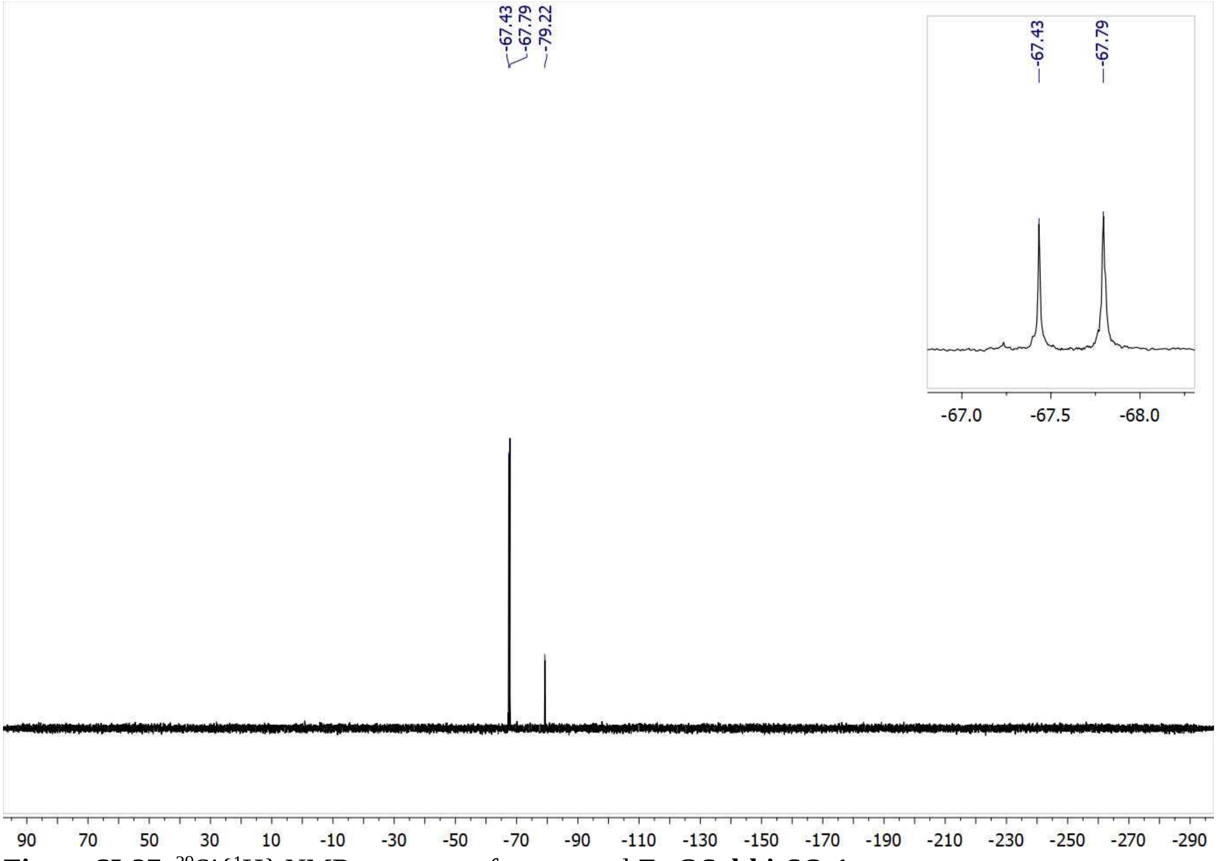

Figure SI-27. $^{29}Si\{^{1}H\}$ NMR spectrum of compound **Zn@Sal-bisSQ-1**.

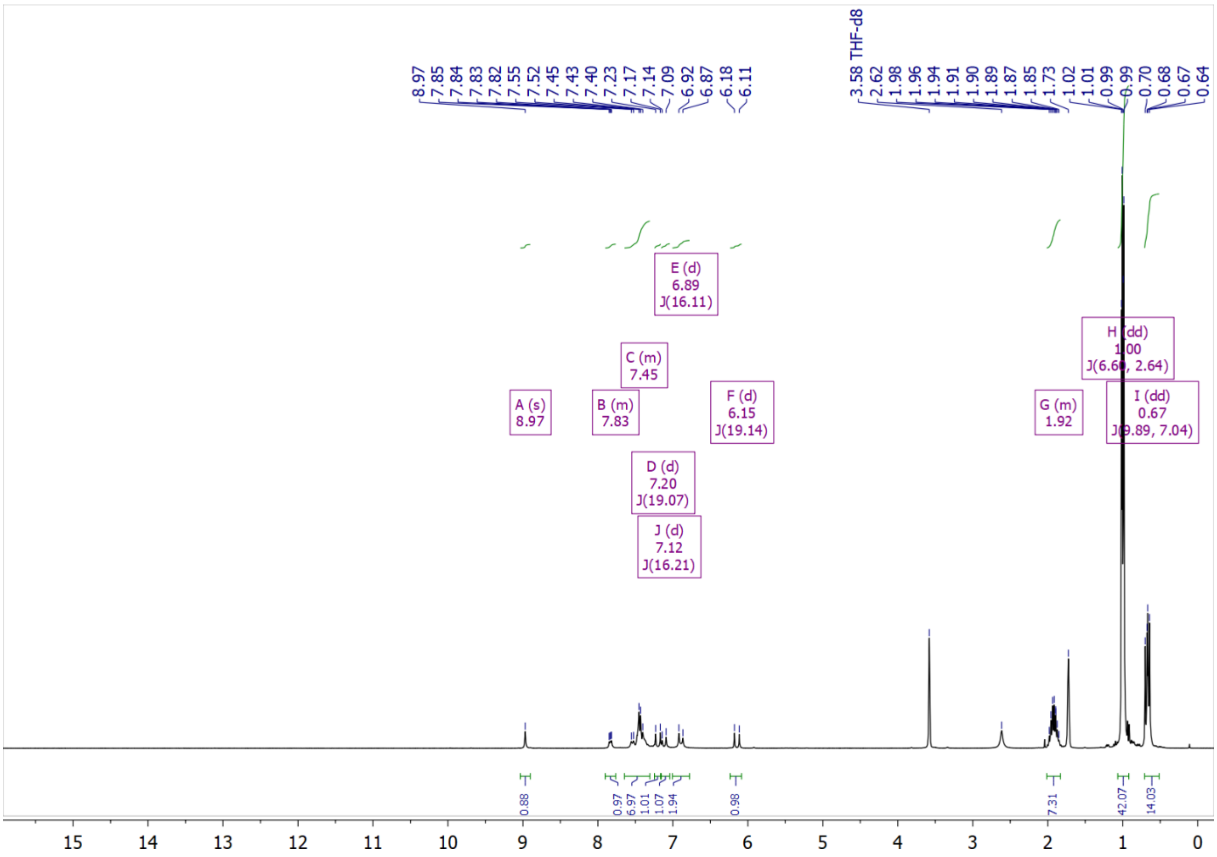

Figure SI-28 ^{1}H NMR spectrum of compound **Zn@Sal-bisSQ-2**.

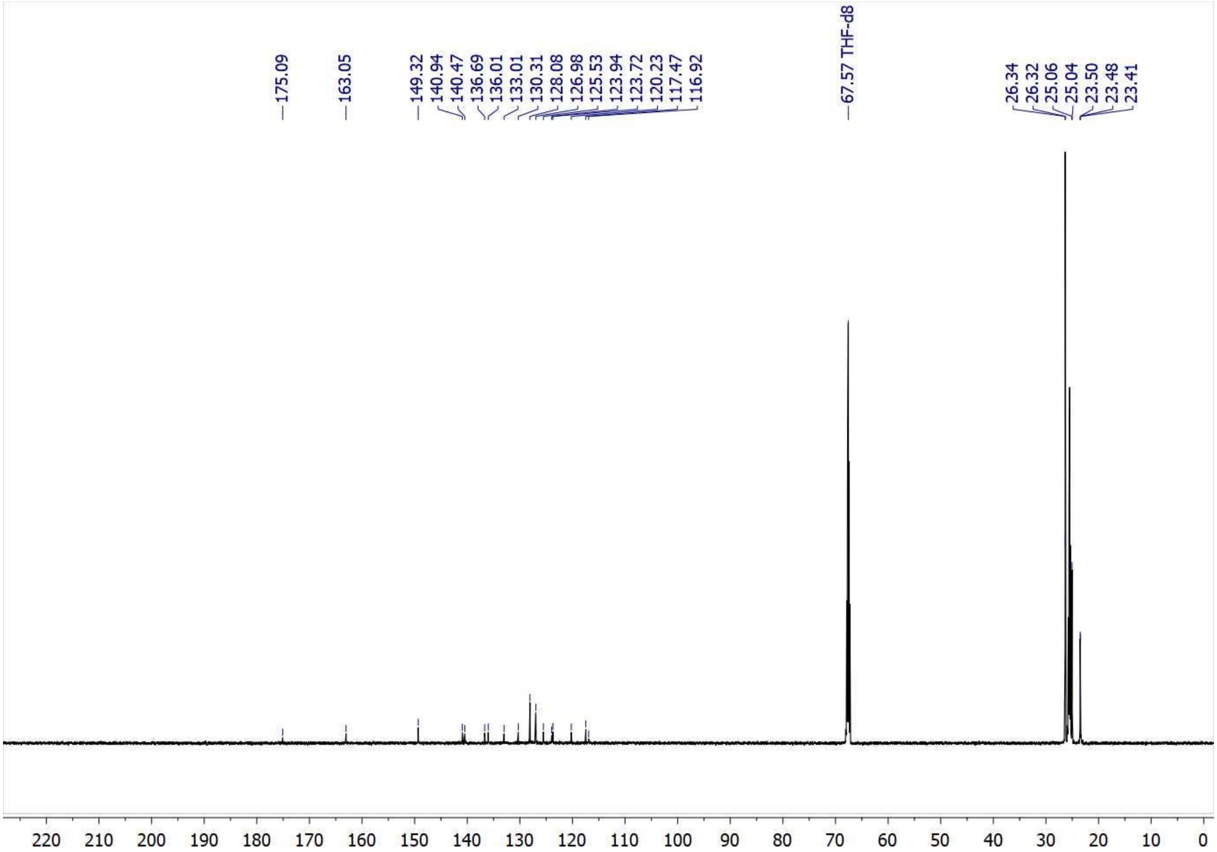

Figure SI-29. $^{13}C\{^{1}H\}$ NMR spectrum of compound **Zn@Sal-bisSQ-2**.

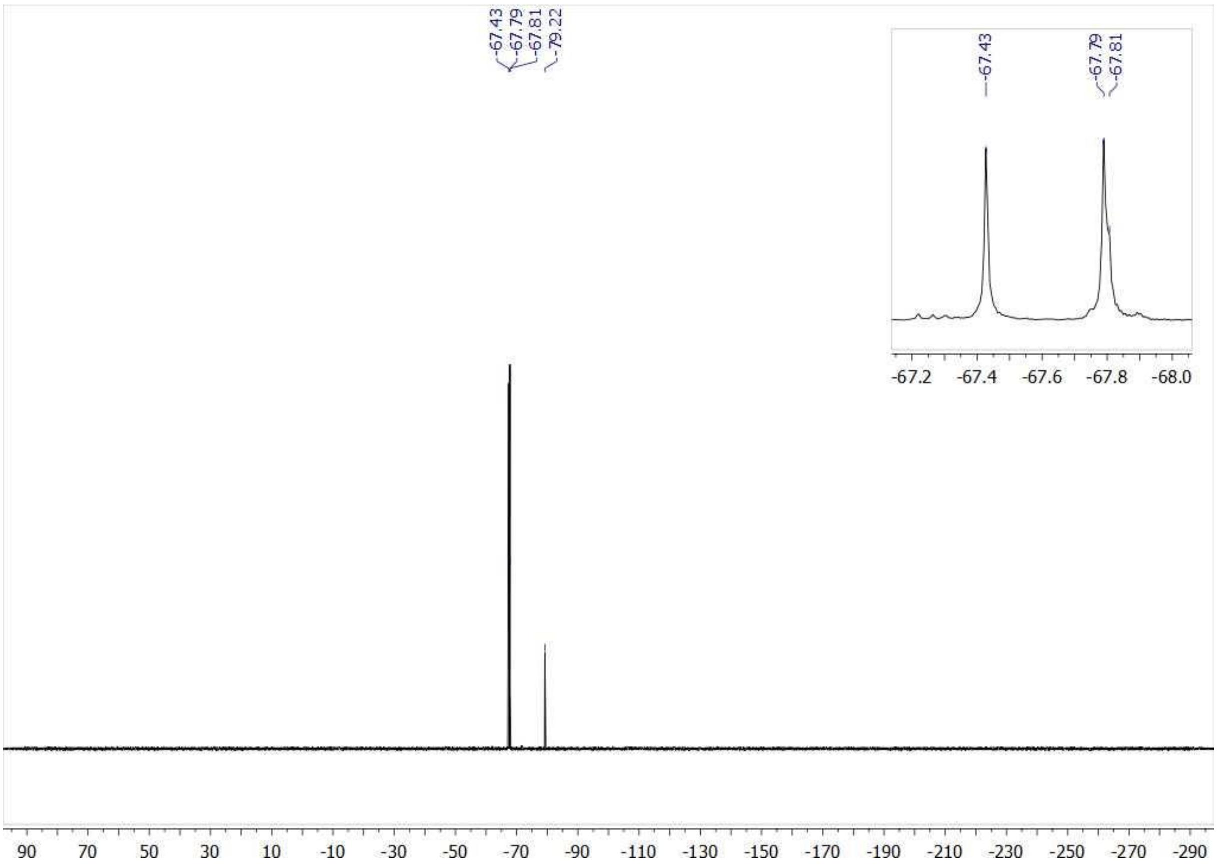

Figure SI-30. $^{29}Si\{^{1}H\}$ NMR spectrum of compound **Zn@Sal-bisSQ-2**.

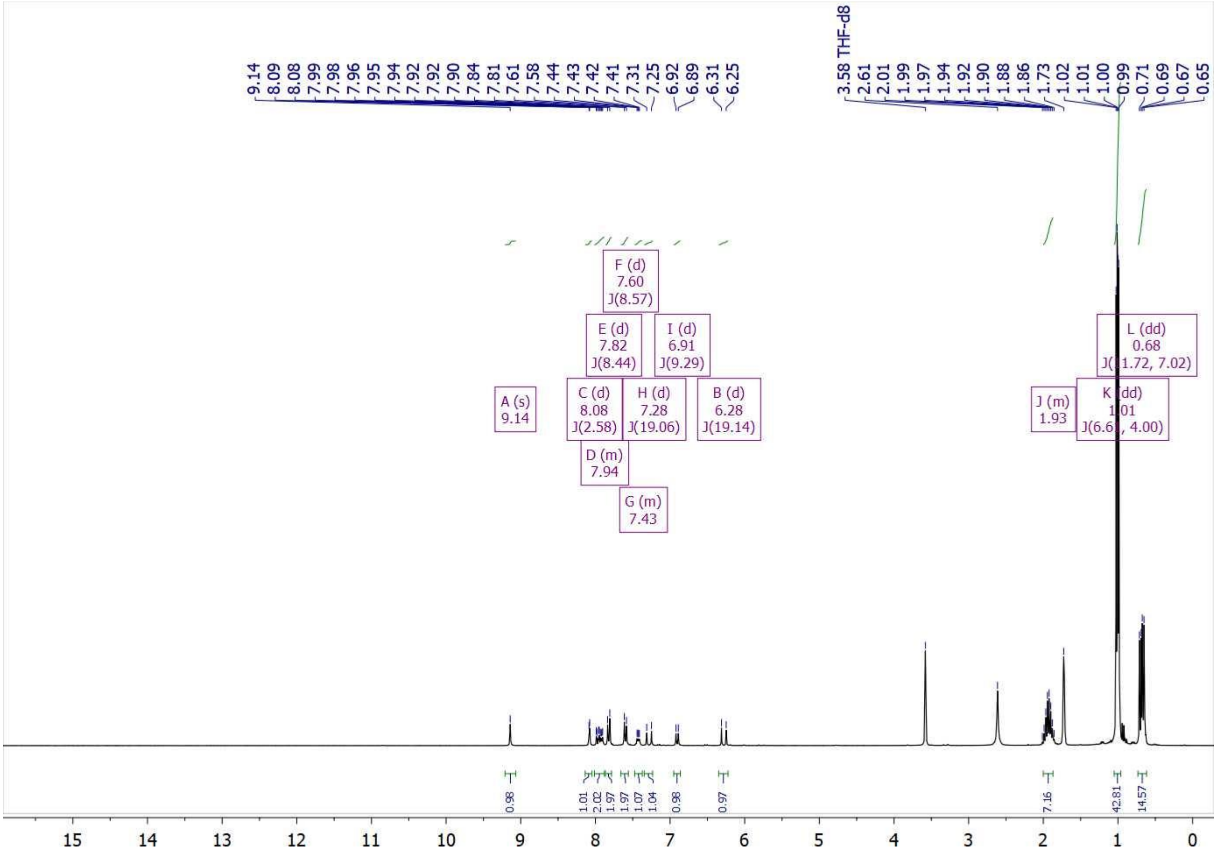

Figure SI-31. ^{1}H NMR spectrum of compound **Zn@Sal-bisSQ-3**.

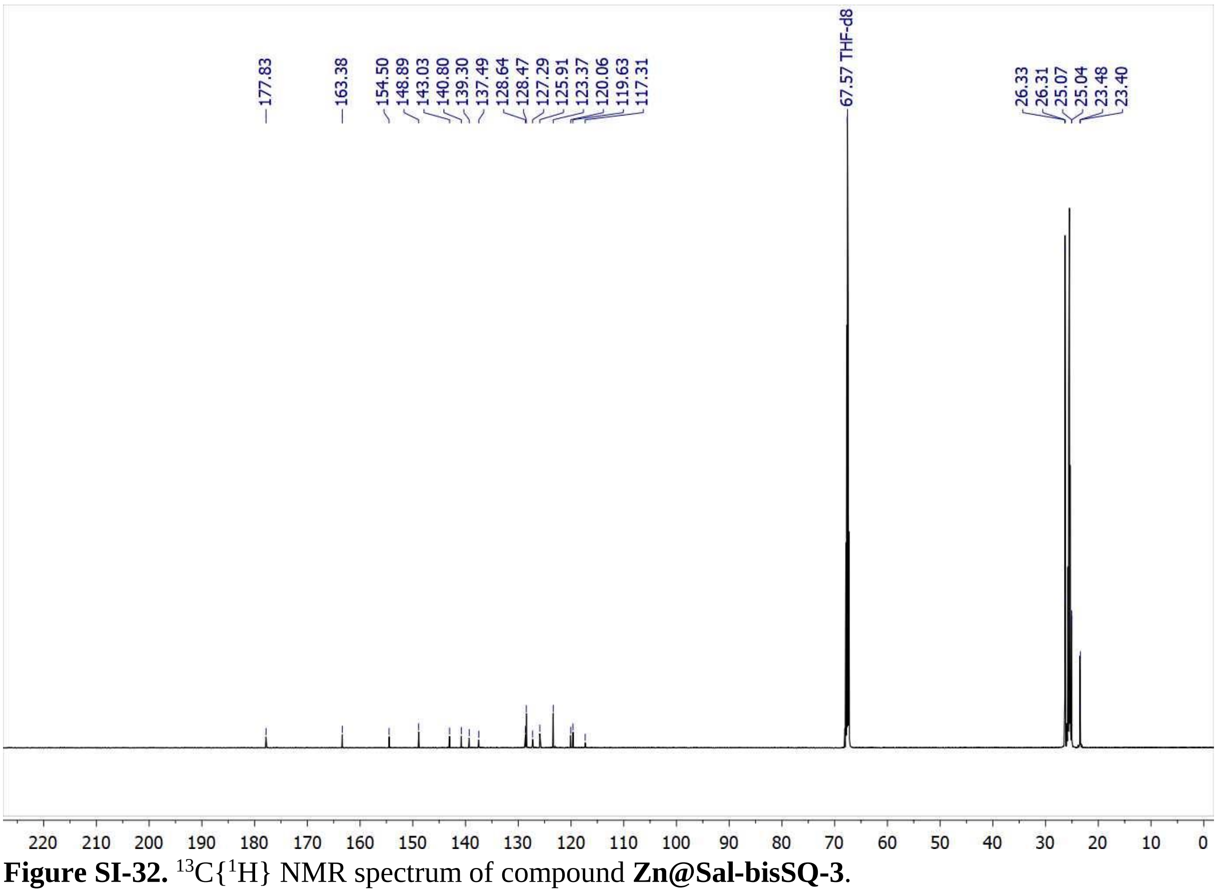

Figure SI-32. $^{13}C\{^{1}H\}$ NMR spectrum of compound **Zn@Sal-bisSQ-3**.

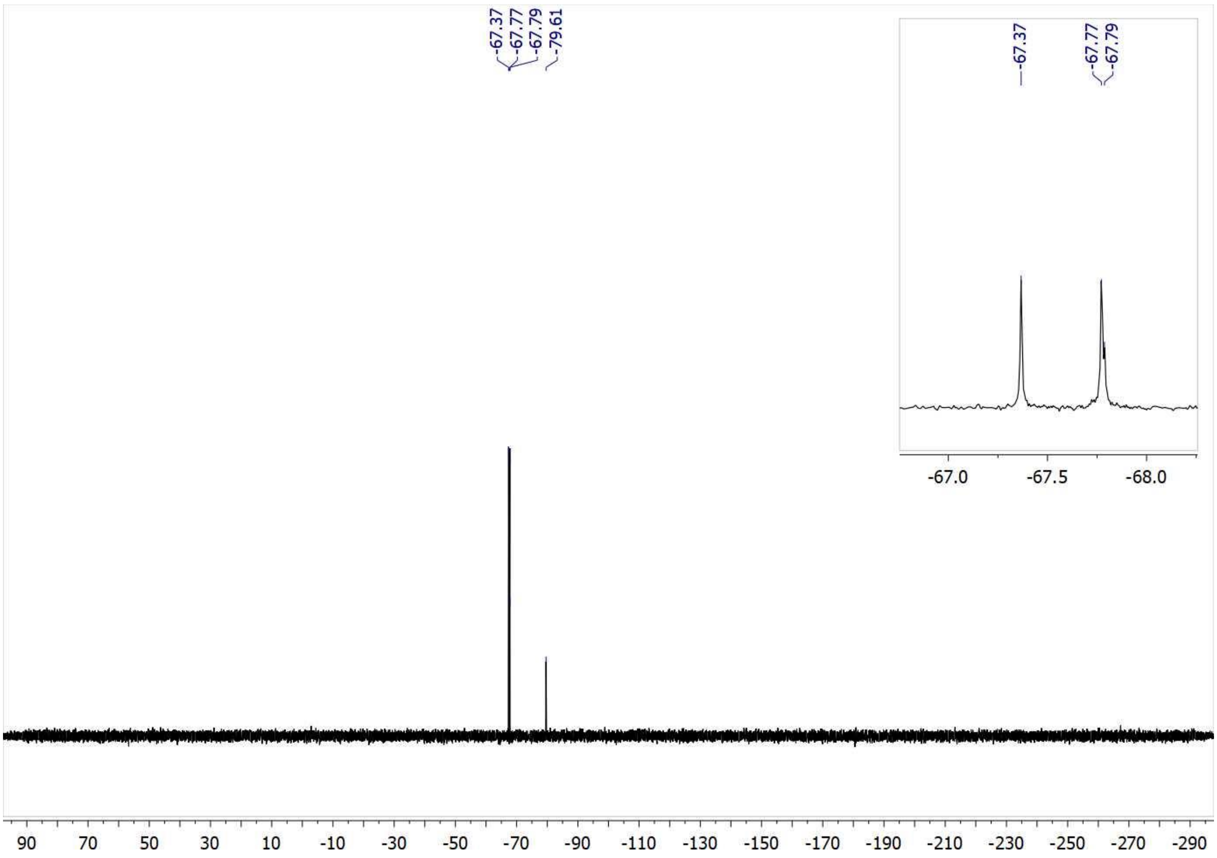

Figure SI-33. $^{29}Si\{^1H\}$ NMR spectrum of compound **Zn@Sal-bisSQ-3**.

4. Photochemistry data

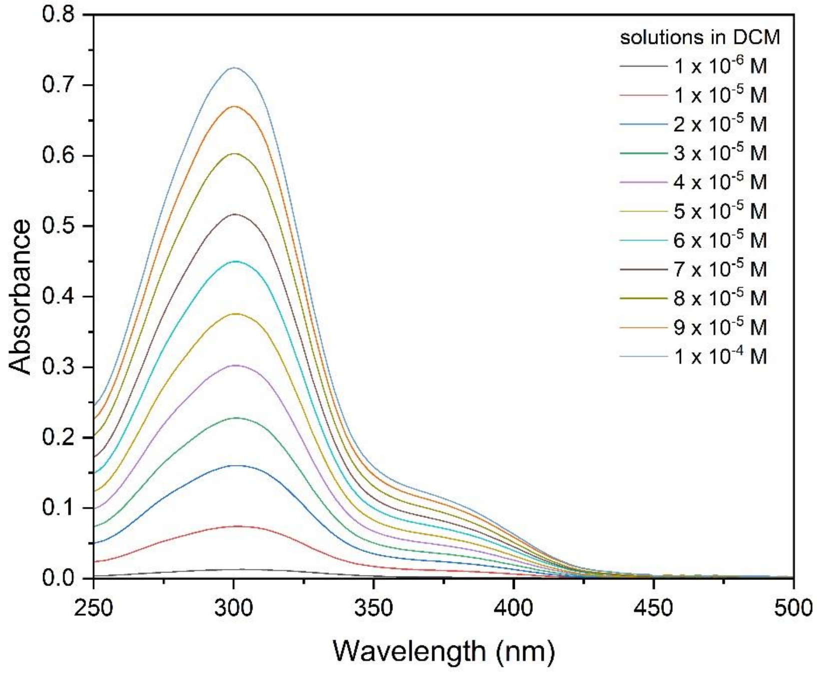

Figure SI-P1. Absorption spectra of **H_2Sal-bisSQ-1**.

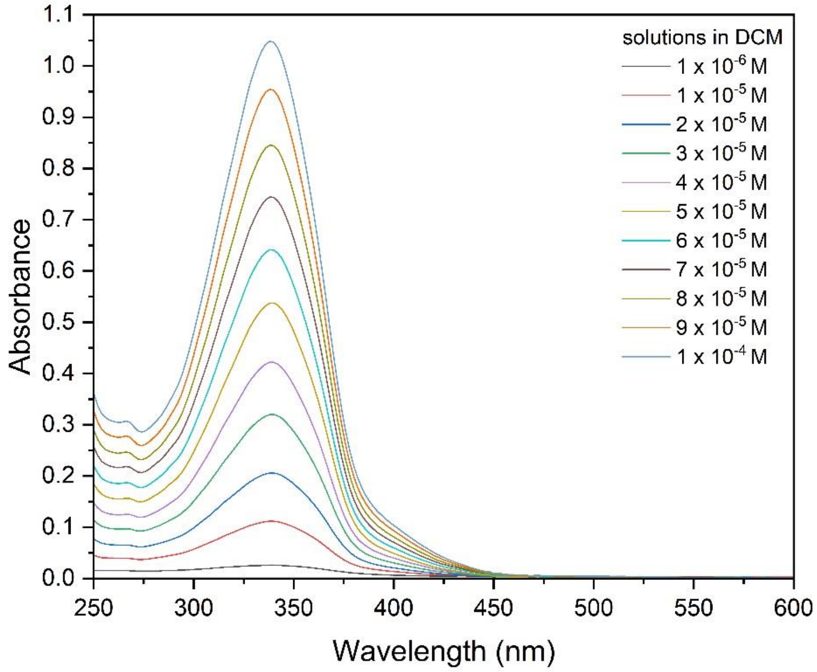

Figure SI-P2. Absorption spectra of **H_2Sal-bisSQ-2**.

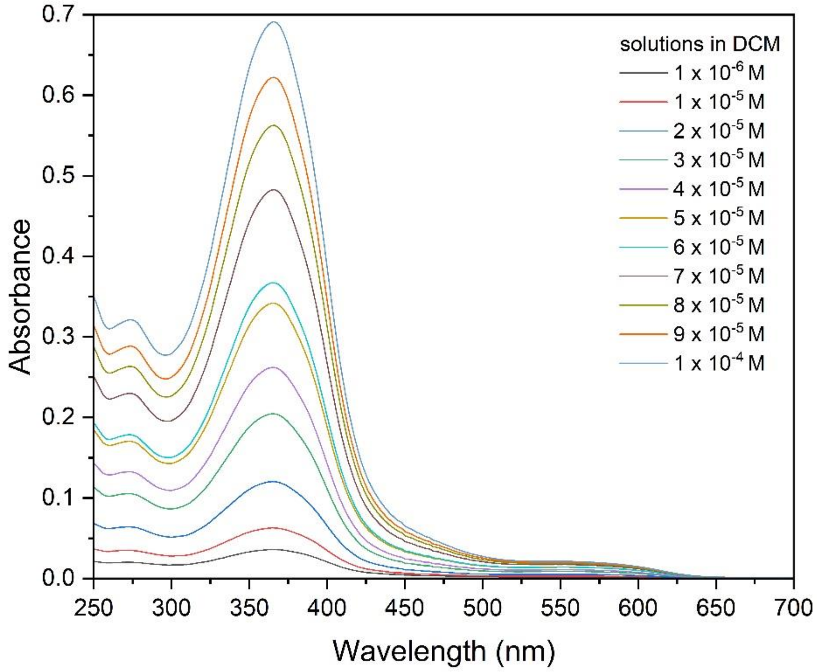

Figure SI-P3. Absorption spectra of **H_2Sal-bisSQ-3**.

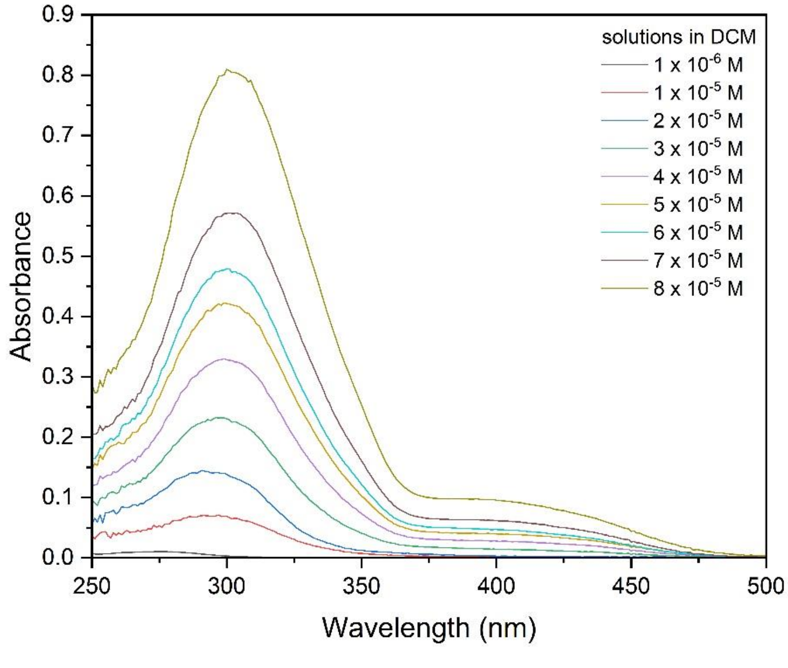

Figure SI-P4. Absorption spectra of **Zn@Sal-bisSQ-1** (solutions in DCM).

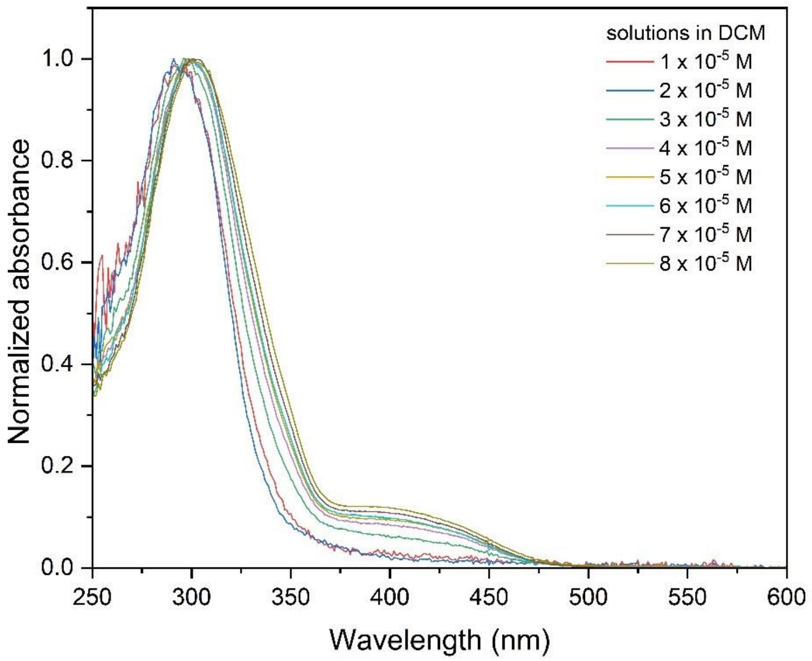

Figure SI-P5. Normalized absorption spectra of **Zn@Sal-bisSQ-1** (solutions in DCM).

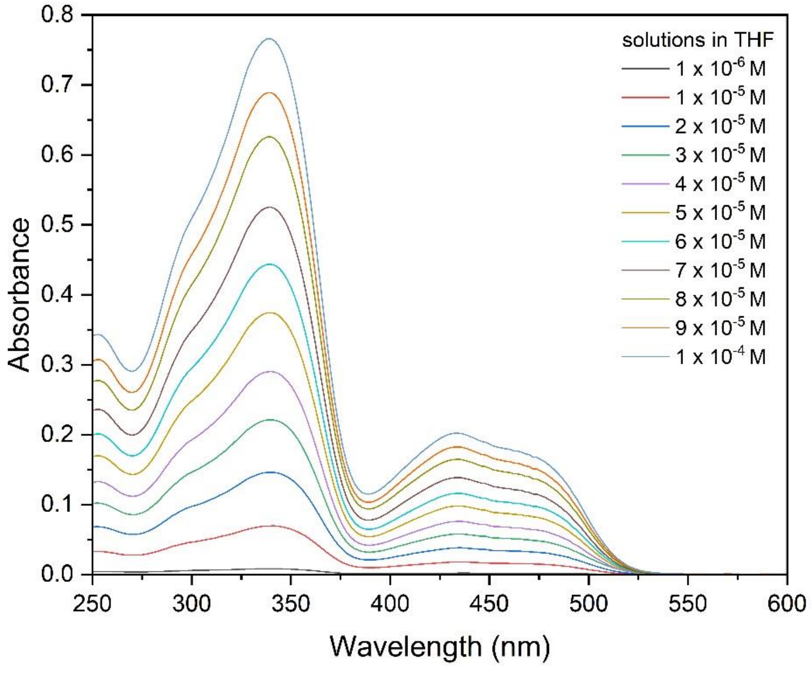

Figure SI-P6. Absorption spectra of **Zn@Sal-bisSQ-1** (solutions in THF).

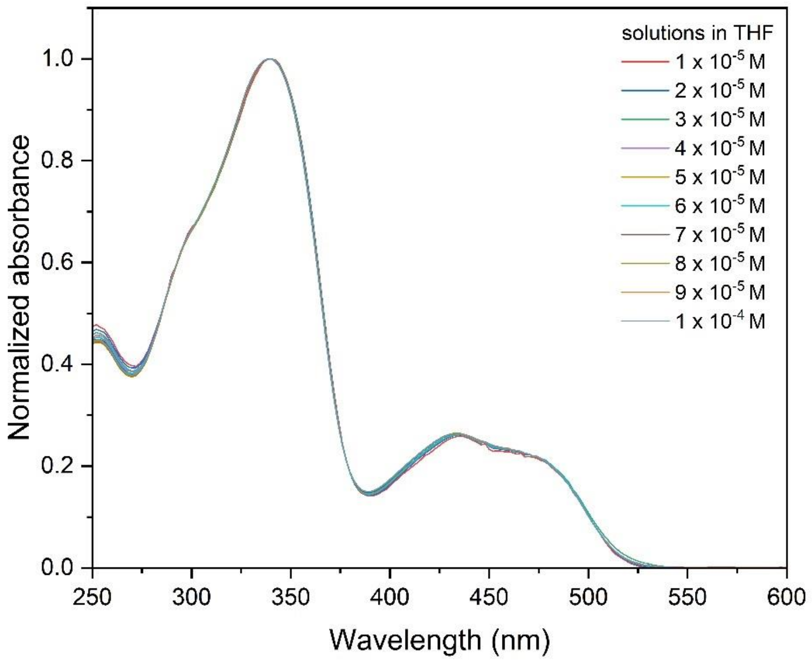

Figure SI-P7. Normalized absorption spectra of **Zn@Sal-bisSQ-1** (solutions in THF).

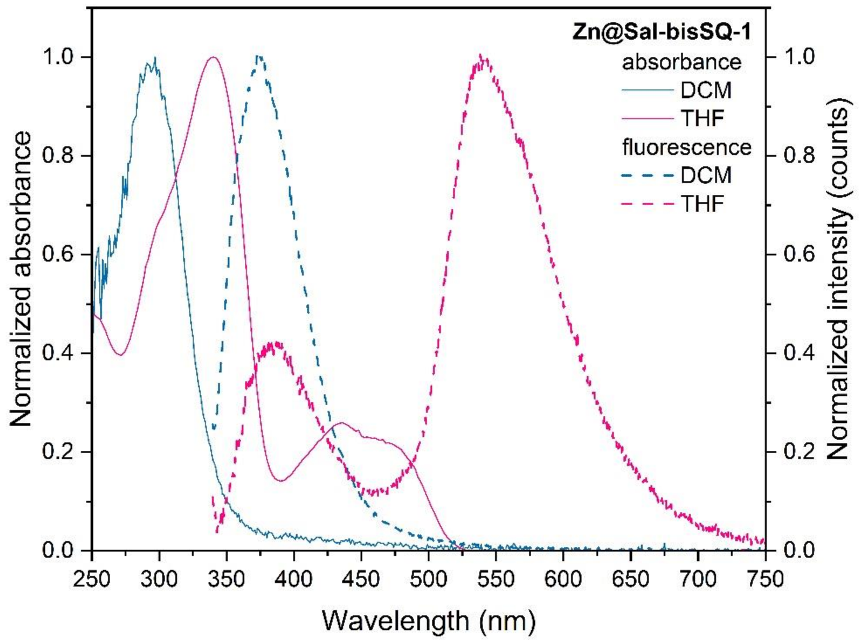

Figure SI-P8 Normalized absorption and fluorescence spectra of **Zn@Sal-bisSQ-1** in DCM and THF ($c = 1\times10^{-6}$ M, $\lambda_{exc} = 329$ nm).

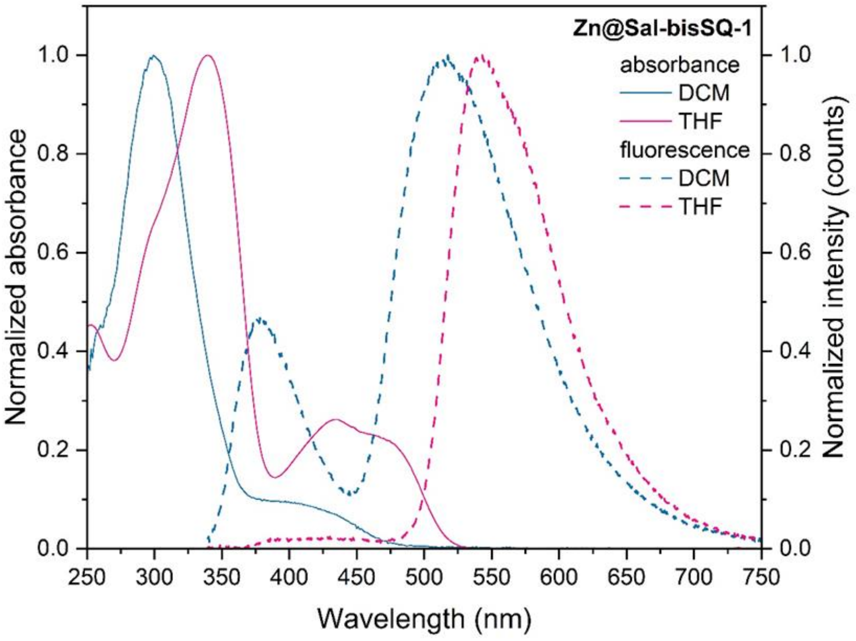

Figure SI-P9 Normalized absorption and fluorescence spectra of **Zn@Sal-bisSQ-1** in DCM and THF (c = 5×10^{-5} M, λ_{exc} = 329 nm).

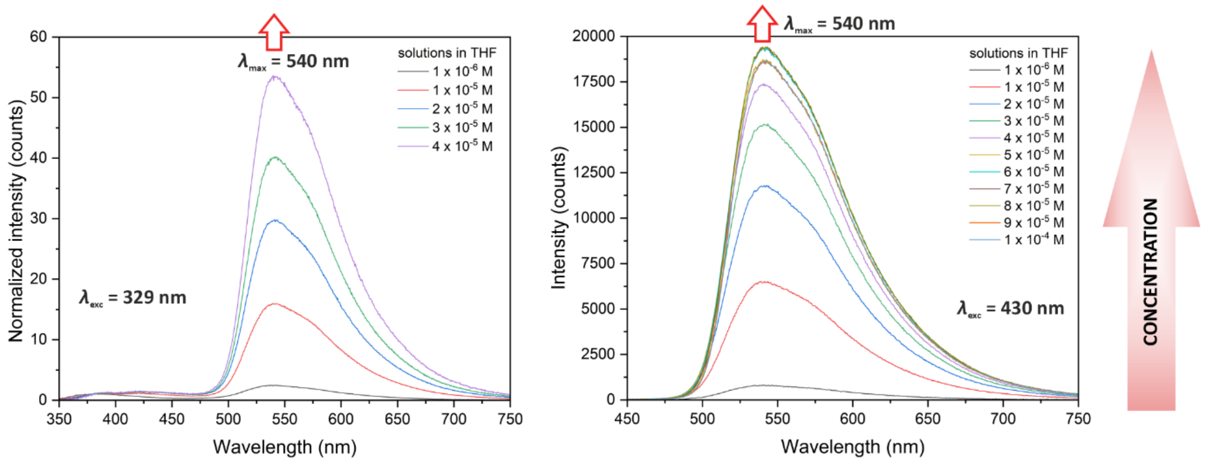

Figure SI-P10. Emission spectra of **Zn@Sal-bisSQ-1** (in THF); λ_{exc} = 329 nm and 430 nm.

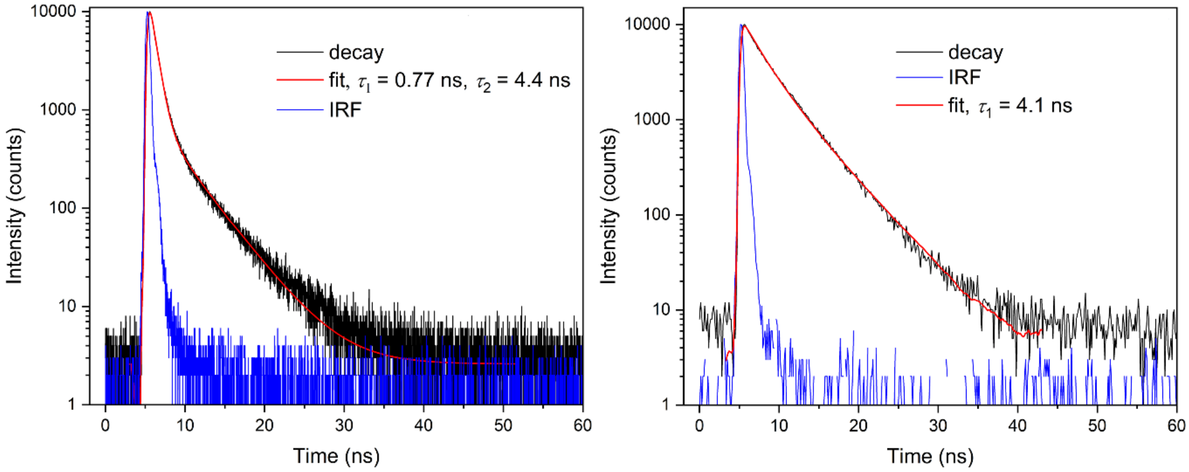

Figure SI-P11. Time-resolved fluorescence decays of **Zn@Sal-bisSQ-1** complex in DCM with excitation at 329 nm and 408 nm. Sample concentration: $1{\times}10^{-5}$ M.

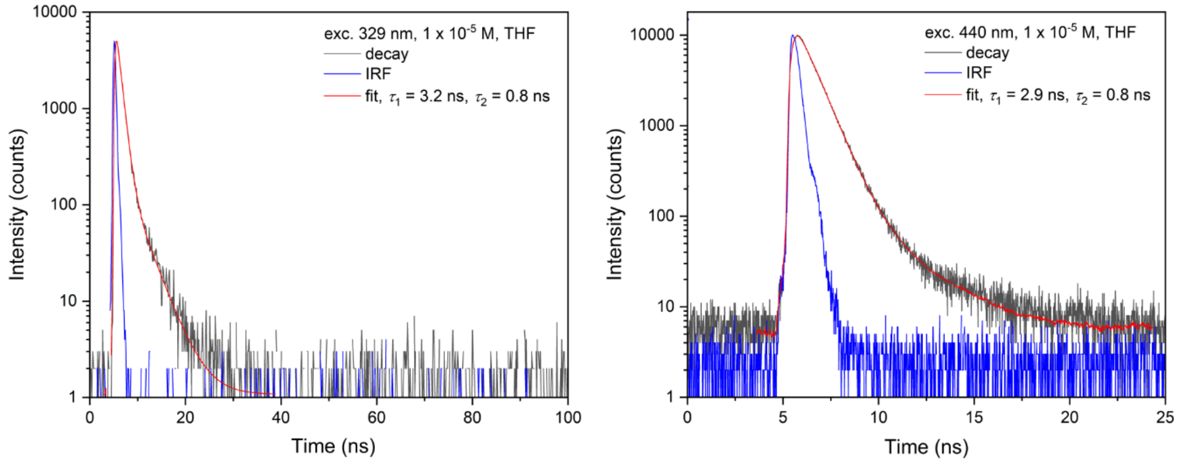

Figure SI-P12. Time-resolved fluorescence decays of **Zn@Sal-bisSQ-1** complex in THF with excitation at 329 nm and 440 nm. Sample concentration: $1{\times}10^{-5}$ M.

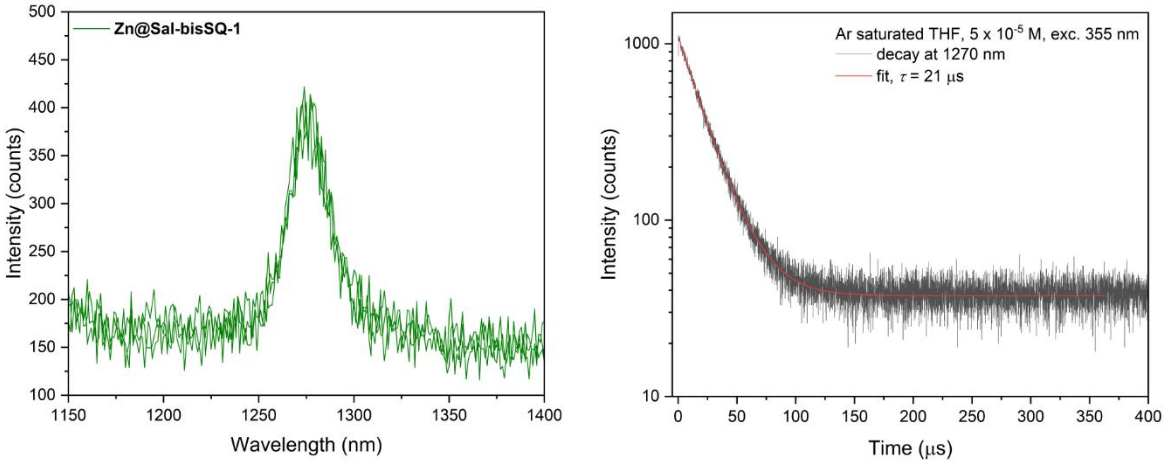

Figure SI-P13. Singlet oxygen phosphorescence spectra obtained for air-equilibrated THF solution of **Zn@Sal-bisSQ-1** and decay trace of **Zn@Sal-bisSQ-1** collected at λ_{max} = 1270 nm (λ_{exc} = 355 nm) in THF. Sample concentration: $5{\times}10^{-5}$ M.

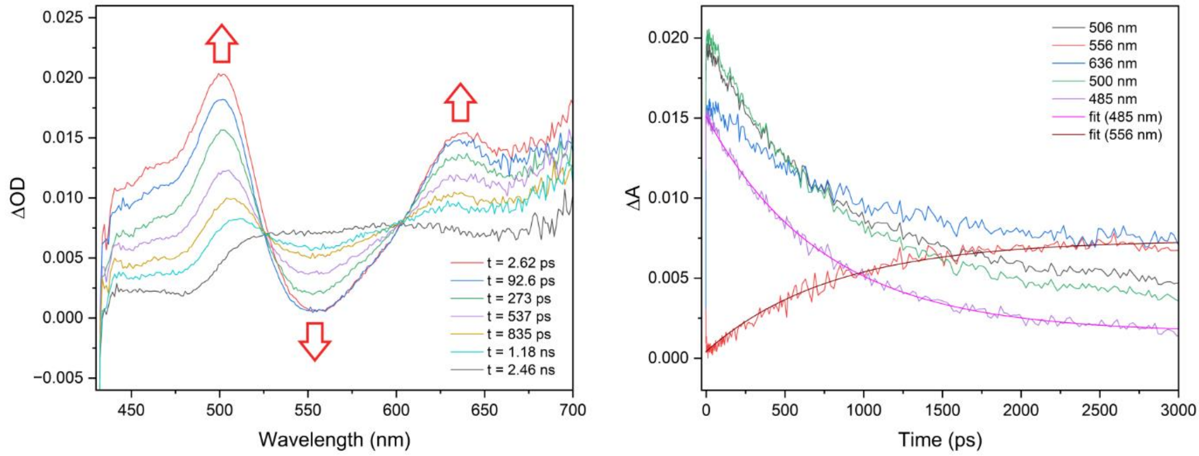

Figure SI-P14. Femtosecond transient absorption spectra registered at different time delays for THF solution of **Zn@Sal-bisSQ-1**.

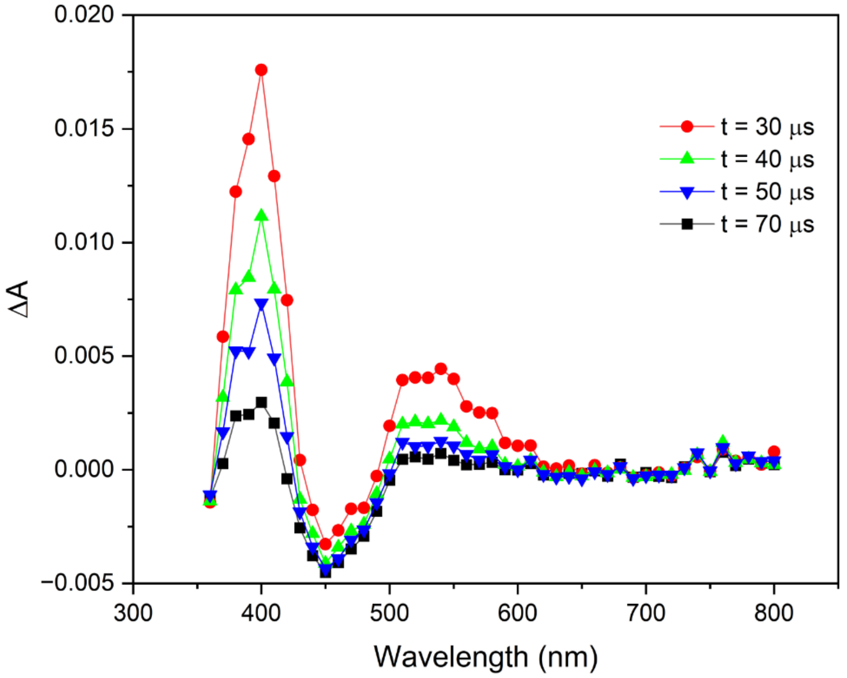

Figure SI-P15. Nanosecond Laser Flash Photolysis (λ_{exc} = 435 nm) of **Zn@Sal-bisSQ-1** in THF – transient absorption spectra after different time delays following the 435 nm laser pulse. Sample concentration: 3×10^{-5} M.

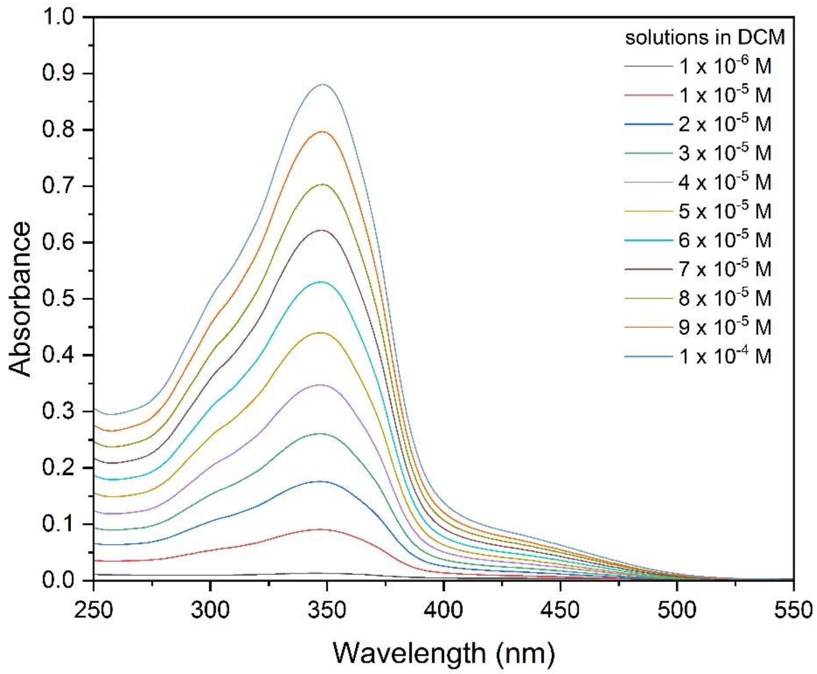

Figure SI-P16. Absorption spectra of **Zn@Sal-bisSQ-2**.

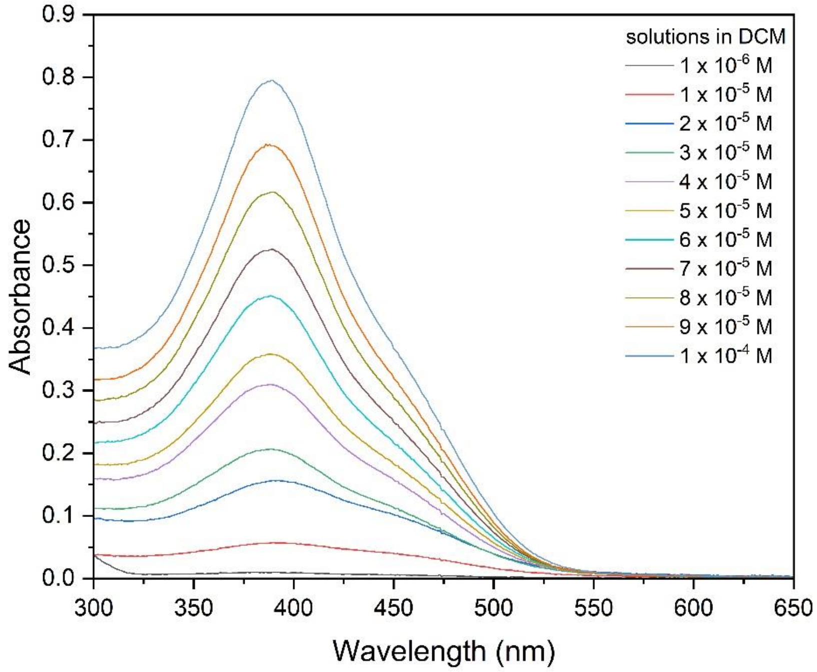

Figure SI-P17. Absorption spectra of **Zn@Sal-bisSQ-3**.

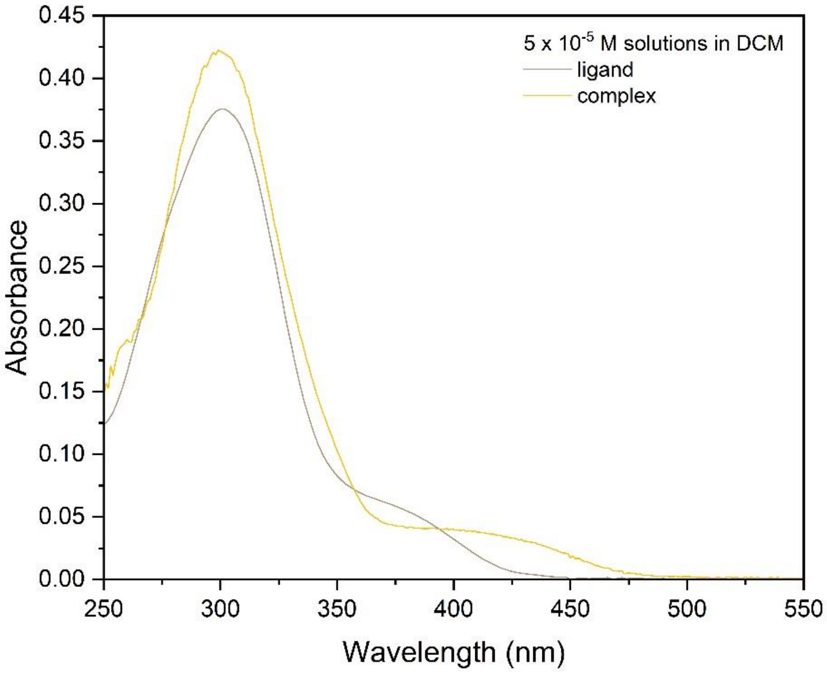

Figure SI-P18. Comparison of absorption spectra measured for ligand **H_2Sal-bisSQ-1** and complex **Zn@Sal-bisSQ-1**.

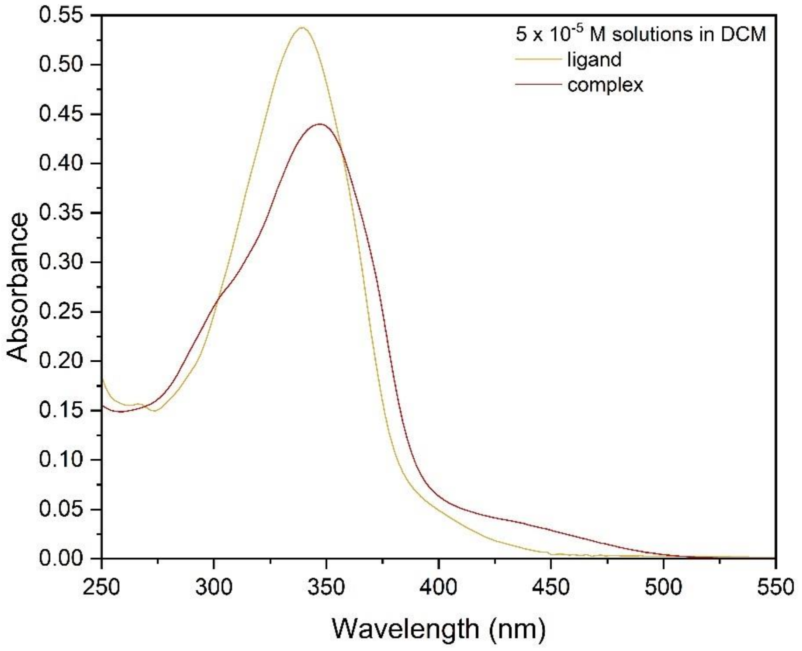

Figure SI-P19. Comparison of absorption spectra measured for ligand **H_2Sal-bisSQ-2** and complex **Zn@Sal-bisSQ-2**.

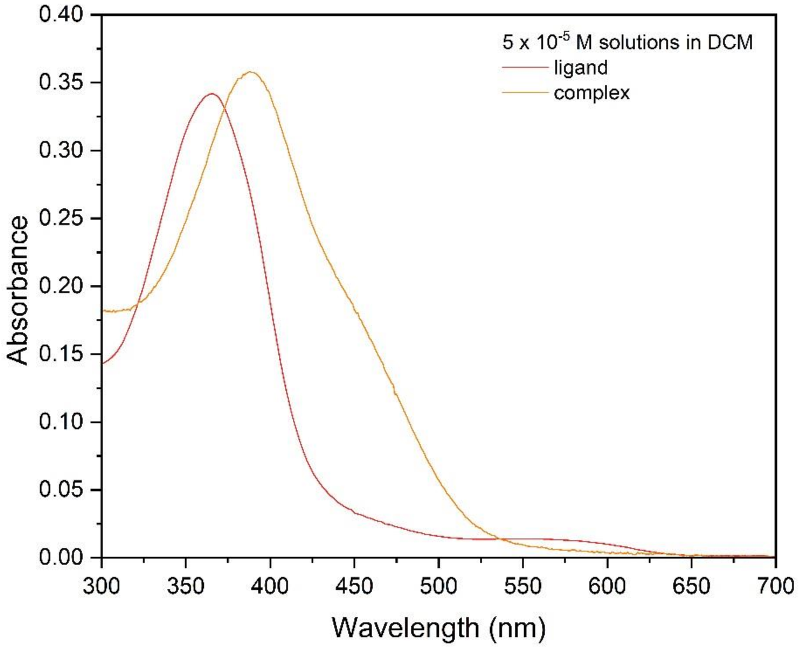

Figure SI-P20. Comparison of absorption spectra measured for ligand **H_2Sal-bisSQ-3** and complex **Zn@Sal-bisSQ-3**.

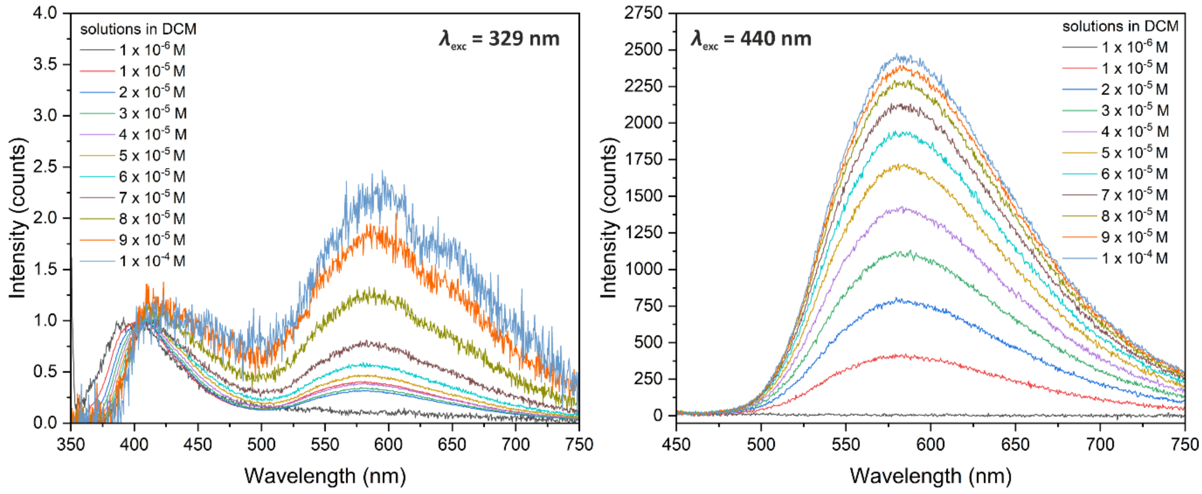

Figure SI-P21. Emission spectra of **Zn@Sal-bisSQ-2**; λ_{exc} = 329 nm and 440 nm.

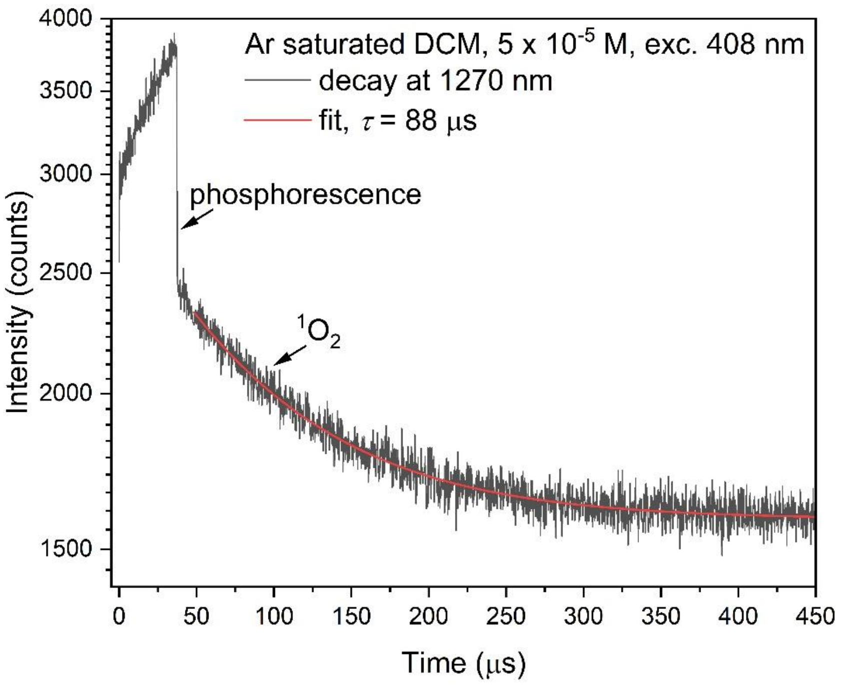

Figure SI-P22. Decay trace of **Zn@Sal-bisSQ-2** collected at λ_{max} = 1270 nm (λ_{exc} = 408 nm) in DCM (sample concentration: 5×10^{-5} M).
